# Phenotypic and Transcriptomic Analysis of Peripheral Blood Plasmacytoid and Conventional Dendritic Cells in Early Drug Naïve Rheumatoid Arthritis

**DOI:** 10.3389/fimmu.2018.00755

**Published:** 2018-05-09

**Authors:** Faye A. H. Cooles, Amy E. Anderson, Andrew Skelton, Arthur G. Pratt, Mariola S. Kurowska-Stolarska, Iain McInnes, Catharien M. U. Hilkens, John D. Isaacs

**Affiliations:** ^1^Institute of Cellular Medicine, Newcastle University and National Institute for Health Research Newcastle Biomedical Research Centre at Newcastle upon Tyne Hospitals NHS Foundation Trust and Newcastle University, Newcastle upon Tyne, United Kingdom; ^2^Arthritis Research UK Rheumatoid Arthritis Pathogenesis Centre of Excellence (RACE), Newcastle University, Newcastle upon Tyne, United Kingdom; ^3^Institute of Infection, Immunity and Inflammation, University of Glasgow, United Kingdom; ^4^Arthritis Research UK Rheumatoid Arthritis Pathogenesis Centre of Excellence (RACE), University of Glasgow, Glasgow, United Kingdom

**Keywords:** rheumatoid arthritis, early rheumatoid arthritis, plasmacytoid dendritic cells, conventional dendritic cells, tolerance

## Abstract

**Objective:**

Dendritic cells (DCs) are key orchestrators of immune function. To date, rheumatoid arthritis (RA) researchers have predominantly focused on a potential pathogenic role for CD1c+ DCs. In contrast, CD141+ DCs and plasmacytoid DCs (pDCs) have not been systematically examined, at least in early RA. In established RA, the role of pDCs is ambiguous and, since disease duration and treatment both impact RA pathophysiology, we examined pDCs, and CD1c+ and CD141+ conventional DCs (cDCs), in early, drug-naïve RA (eRA) patients.

**Methods:**

We analyzed the frequency and phenotype of pDCs, CD1c+, and CD141+ DCs from eRA patients and compared findings with healthy controls. In parallel, we performed transcriptional analysis of >600 immunology-related genes (Nanostring) from peripheral blood pDCs, CD1c+ DCs, B cells, T cells, and monocytes.

**Results:**

All DC subsets were reduced in eRA (*n* = 44) compared with healthy controls (*n* = 30) and, for pDCs, this was most marked in seropositive patients. CD141+ and CD1c+ DCs, but not pDCs, had a comparatively activated phenotype at baseline (increased CD86) and CD1c+ DC frequency inversely associated with disease activity. All DC frequencies remained static 12 months after initiation of immunomodulatory therapy despite a fall in activation markers (e.g., HLA-DR, CD40). There was no association between the whole blood interferon gene signature (IGS) and pDC or CD1c+ DC parameters but an inverse association between CD141+ DC frequency and IGS was noted. Furthermore, IFN-I and IFN-III mRNA transcripts were comparable between eRA pDC and other leukocyte subsets (B cells, CD4+, and CD8+ T cells and monocytes) with no obvious circulating cellular source of IFN-I or IFN-III. Transcriptomic analysis suggested increased pDC and CD1c+ DC proliferation in eRA; pDC differentially expressed genes also suggested enhanced tolerogenic function, whereas for CD1c+ DCs, pro-inflammatory transcripts were upregulated.

**Discussion:**

This is the first detailed examination of DC subsets in eRA peripheral blood. Compared with CD1c+ DCs, pDCs are less activated and may be skewed toward tolerogenic functions. CD141+ DCs may be implicated in RA pathophysiology. Our findings justify further investigation of early RA DC biology.

## Introduction

Dendritic cells (DCs) are professional antigen-presenting cells (APCs), which orchestrate immune responses. They provide a link between the innate and adaptive immune system by translating non-specific danger and damage signals into a targeted antipathogen response (1). DCs continuously sample their environment by micropinocytosis allowing uptake of both self and non-self proteins, which are subsequently trafficked to the cell membrane and displayed in an antigen–major histocompatibility complex (MHC) complex (2). DC receptors can sense danger, microbe, or cytokine signals, which, when triggered, drive DC maturation and activation. This promotes migration to lymph nodes, further proinflammatory cytokine release, increased stability of the antigen–MHC complex, and upregulation of co-stimulatory molecules, such as CD40 and CD86. Together, these support a targeted T cell response against the presented antigen. Thus, if activated/mature DCs present self-antigen tolerance may be breached highlighting the need for tight regulation of DC biology and their importance in autoimmunity (2, 3). Some DCs, while retaining APC capacity, are resistant to maturation-inducing signals and downregulate co-stimulatory molecule expression and pro-inflammatory cytokines while simultaneously upregulating the expression of inhibitory molecules and anti-inflammatory cytokines (4, 5). These so-called regulatory or tolerogenic DCs promote T cell anergy and regulatory T cell (Treg) generation and are being explored as a potential therapy in autoimmune disease (6–11).

Dendritic cells are a heterogeneous population and can be divided into conventional myeloid (cDCs) and plasmacytoid (pDCs) (12). cDCs express typical myeloid antigens but can be sub-divided by their expression of CD141 and CD1c, thereby generating two additional subsets; CD141+ DC (cDC1) and CD1c+ DC (cDC2), respectively. pDCs typically lack these markers, although can express low levels of CD141, and express instead CD123, CD303, and CD304. Typically both cDC and pDC subsets display APC capacity, but this function is emphasized in cDCs (13). CD141+ DC have been suggested to cross-present antigen and efficiently prime CD8+ T cells and CD1c+ DC are capable of priming CD4+ and CD8+ T cells (14). Conversely, pDCs have relatively reduced HLA-DR (MHC class II) expression (13) and are the primary type 1 interferon (IFN-I) producing cell subset, a key early cytokine in the immune defense against viral infection (15).

Dendritic cells are believed to be important in RA pathogenesis, indeed, HLA-DR variants are linked to RA susceptibility implicating DC activation of autoreactive lymphocytes in disease onset (16). Furthermore, another DC subset termed inflammatory DC, which are thought to be derived from monocytes, have been reported to be the main DC subset in the synovial fluid of RA patients and are involved in the induction and maintenance of Th17 cell responses (17–23). There is additional evidence that cDCs are involved in early breach of tolerance in animal RA models (24). However, previous studies have often not distinguished between CD141+ cDC1 and CD1c+ cDC2 subsets and referred to them collectively as myeloid cDCs or just examined CD1c+ DCs alone. Indeed, CD1c+ DCs have been found at high levels in synovial joint tissue and fluid from RA patients where they can promote pathological Th1 cytokines (25, 26). With regard to circulating CD1c+ DC in RA patients there are contrasting reports. Most studies have reported a decrease in their numbers with a comparatively immature phenotype (25, 27–31), whereas one reported an increase in number (32). Additionally, in established RA patients, CD1c+ DC levels inversely correlated with disease activity and low DC numbers were restored upon successful treatment and fall in disease burden (29). However, this was not a universal finding for all therapeutic regimens (30). Examination of CD141+ DCs specifically in RA has been limited to one published abstract where they are increased in established RA synovial fluid, displaying a relatively mature phenotype (33). Nonetheless, studies of the presumed murine equivalent demonstrate an acceleration in the onset of collagen-induced arthritis following their adoptive transfer with CD4+ T cells (34).

Conversely, while pDCs are also increased in the synovial compartment and reduced in the peripheral blood of established RA patients, the circulating pDCs are immature and numbers do not correlate with disease activity (25, 27, 29, 31, 35). Furthermore, when compared with CD1c+ DCs, synovial pDCs also have a more immature phenotype (25). Some, therefore, propose that, in RA, pDCs have an anti-inflammatory function in the context of breach of tolerance (35–37). However, this role has not been universally supported (38, 39) and the net role of pDCs in RA pathogenesis remains ambiguous. Their capacity to produce large amounts of IFN-I is likely to be important given the emerging association of the interferon gene signature (IGS) with autoimmunity. The IGS is a composite score of genes upregulated upon exposure to IFN-I [interferon response genes (IRGs)]. In both early and established RA, a subset of patients have a raised IGS, which impacts on the clinical response to certain therapies (40–45). Exposure to IFN-I is likely to be important as genetic variants that increase RA susceptibility are associated with the IFN-I pathway (46, 47) and upregulation of genes related to the IFN-I signaling pathway predicted progression to RA in seropositive arthralgia (48, 49). Furthermore, administration of IFN-α can promote an inflammatory arthritis phenotype in humans (50) and transfer of IFN-I producing DCs propagates a persistent inflammatory arthritis in mice (51). RA therapies can influence the IGS (44, 52) and, therefore, complicate study of IFN biology in RA.

Given the central role of DCs in all immune responses, and their proposed role in RA and other autoimmune inflammatory diseases, we examined CD1c+ DC, CD141+ DC, and pDCs in patients in the early stages of RA, before treatment with antirheumatic drugs.

## Materials and Methods

### Patient Cohorts

Glucocorticoid and disease-modifying antirheumatic drug (DMARD)-naïve patients were recruited from the Newcastle Early Arthritis Cohort, Newcastle upon Tyne Hospitals, UK at the point of their initial consultant rheumatologist diagnosis of RA (early RA) with reference to 2010 ACR/EULAR RA classification criteria. Clinical parameters including DAS-28-ESR, its components, inflammatory markers, disease duration, early morning stiffness, and serological status (rheumatoid factor, RF; anti-cyclic citrullinated peptide, anti-CCP) were recorded. Healthy controls with no history of autoimmunity were also recruited. Circulating serum cytokines IFN-γ, IL-6, IL-12 p70, TNF-α, IL-1β, IL-2, IL-13, IL-4, IL-10 were measured by MSD technology (Meso Scale Discovery, MD, USA) and B-Cell activating factor (BAFF) was measured using ELISA (R & D Systems GmbH, Germany) as per manufacturers instructions. This study obtained full ethical approval from the North East—Newcastle & North Tyneside 2 Research Ethics Committee (REC reference: 12/NE/0251).

### DC Phenotyping and Sorting

Peripheral blood mononuclear cells were isolated by density centrifugation (Lymphoprep, Axis-Shield Diagnostics Ltd., UK), washed, and re-suspended in staining buffer (phosphate-buffered saline supplemented with 1% fetal calf serum, 2 mM EDTA and 0.01% sodium azide) in the presence of IgG. Cell surface expression was assessed following 30 min incubation with the following antibodies: CD3-FITC (HIT3a), CD19-FITC (HIB19), CD20-FITC (2H7), CD203c-FITC (NP4D6), CD11c-PerCP-Cy5.5 (BU15), CD123-BV650 (6H6), HLA-DR-AF700 (L243), CD141-BV711 (1A4), CD14-BV510 (M5E2), CD1c-APC-Cy7 (L161) all from Biolegend, CA, USA; CD86-APC (FUN1) and CD40-PE (C40-1457) from BD Biosciences (Oxford, UK); CCR7-PE (150503) from RnD Systems, Abigndon, UK and live/dead *CyStain*^®^ from Partec Japan. Acquisition was on a BD LSR Fortessa™ and analyzed using FlowJo software (Treestar). pDCs, CD1c+ DCs, and CD141+ DCs were defined as CD19−CD20−CD3−CD203c−HLA-DR+CD14−CD1c−CD141− or ^dim^CD11c− CD123+, CD19−CD20−CD3−CD203c−HLA−DR+CD14−CD1c+CD141− or ^dim^CD11c+ and CD19−CD20−CD3−CD203c−HLA-DR+CD14−CD1c+CD141^high^CD11c−, respectively (gating strategy shown in Figure S1 in Supplementary Material). Equivalent pDC and CD1c+ DCs as well as B cells (CD19+, CD20+, CD14−, CD3−), CD4+ T cells (CD19−, CD20−, CD14−, CD3+, CD4+, CD8−, CD56−), CD8+ T cells (CD19− CD20−, CD14−, CD3+, CD4−, CD8+, CD56−), and monocytes (CD3−, CD14+) subsets from 4 healthy controls and 8 age- and sex-matched early RA patients were flow-sorted (BD FACSARIA II, Becton Dickinson, NJ, USA) following staining with the following antibodies: CD3-BV786 (UCHT1), CD4-PECy7 (RPA-T4), CD8-PE (HIT8a), CD19-APC (HIB19), CD20-APC (2H7) (all from Biolegend) with CD11c, CD1c, CD123, CD14, HLA-DR, and *CyStain*^®^ as previously listed. Florescence minus one was used for gating and median fluorescence intensity was determined to quantify cell surface expression.

### Transcriptional Analysis

Following homogenization (QIAshredder column, Qiagen, Germany), RNA was isolated from cell sorted pDC, CD1c+ DC, CD4+ T cells, CD8+ T cells, B cell, and monocyte lysates by Qiagen RNeasy Plus Micro Kit (Qiagen, Germany) as per manufacturer’s instructions. 50 ng of RNA from each was loaded onto a NanoString nCounter Human immunology V2 Panel chip (NanoString Technologies Inc., WA, USA) including 594 immunology-related genes. An additional 14 genes were included in a customized chip modification to allow for cell specific quantification of the IGS (Table S2 in Supplementary Material). These notably included *ISG15, IFI6, OAS1*, and *IFI44L*. The nCounter protocol was followed according to manufacturer’s instructions. *CD27* transcript expression was examined in the pDC subset and compared with the B cell compartment to exclude plasma cell contamination.

### Interferon Gene Signature

Whole blood RNA was isolated using the Tempus Spin RNA Isolation Kit (Tempus, ThermoFisher Scientific, MA, USA). RNA was reverse transcribed to cDNA using Superscript II (Thermo-Fisher Scientific, MA, USA). To quantify the expression of IRG *MxA, IFI6, OAS1, ISG15*, and *IFI44L*, gene specific primers were designed and Roche universal probe library used (Table S1 in Supplementary Material) to perform RT-PCR (Taqman gene expression master mix, ThermoFisher). The mean expression of these five genes was termed the IGS score. Patients were defined as exhibiting a positive IGS if their mean IRG expression was ≥2 SDs above the mean healthy control IRG expression (53).

### Statistical and Data Analysis

Univariate generalized linear models, Mann–Whitney *U* tests, one-way ANOVA (with Tukey’s *post hoc* analysis) and Wilcoxon-signed rank tests were performed using GraphPad Prism (ver. 5.0, San Diego, CA, USA), employing a significance threshold where α = 5%. Nanostring analysis was performed in R (v3.3.2), with packages from the Bioconductor repository. Differential expression analysis was performed with DESeq2, due to the data appearing to follow the negative-binomial distribution. Library scaling normalization was performed with DESeq2 prior to fitting the model, and differential expression was tested using the Wald-Test. Statistical significance was accepted where genes FDR corrected *p* values < 0.05 and fold change > 1.5. Ingenuity^®^ Pathway Analysis (IPA^®^) was performed on differentially expressed genes (DEGs).

## Results

### Patient Cohorts

Cohorts included 44 early RA patients and 30 healthy controls. Full demographical data are shown in Table 1A where there were significant differences in age and sex between the cohorts. Some early RA patients (*n* = 15) had DC parameters measured again at 1, 3, 6, and 12 months after initiation of DMARD therapy. Treatment included a single baseline intramuscular glucocor-ticoid injection (*n* = 13) and thereafter methotrexate monotherapy (*n* = 9), hydroxychloroquine, sulfasalazine, or leflunomide monotherapy (*n* = 1 for each) and methotrexate with hydroxychloroquine (*n* = 3). A further 8 early RA and 4 healthy controls (age and sex matched) were recruited for transcriptomic analysis (demographics, Table 1B).

**Table 1:** Patient demographics.

A												
Patient cohort	*N*	Age (years) median (range)	M:F ratio	DAS-28 median (range)	CRP mg/L median (range)	ESR mm/hmedian (range)	RF+ and/or anti-CCP+ (*n*, %)	TJC median (range)	SJC median (range)	Patient VAS median (range)	Symptom duration weeks median (range)	EMS mins median (range)
eRA	44	57 (33–84)	3:4	4.49 (1.4–76)	9 (4–114)	25 (5–76)	34 (77%)	5 (0–20)	3 (1–24)	52 (8–100)	14 (3–52)	60 (0–360)

HC	30	37 (23–62)	3:2	–	–	–	–	–	–	–	–	–

*p-*Value±	–	**0.002**	**0.01**	–	–	–	–	–	–	–	–	–

**B**												

	**eRA**			**HC**	**Difference between IGS+, IGS− eRA, and HC cohorts^a^**	**Difference between IGS+ IGS− eRA cohorts±**						
		
	**IGS+**	**IGS−**	**All eRA**									

Number (*n*)	4	4	8	4	–	–						

Age (years) median (range)	56(49–64)	54.5 (53–60)	55 (49–64)	53 (48–62)	*p* = 0.905	*p* = 0.771						

M:F ratio	3:1	3:1	3:1	1:1	*p* = 0.750	*p* = 0.847						

Median IGS Score	0.001156	0.000203	0.00071	0.000198	***p* = 0.0003**	***p* = 0.028**						

DAS-28 median (range)	3.4 (2.63–4.28)	4.38 (1.63–6.18)	3.71 (1.63–6.18)	–	–	*p* = 0.689						

CRP mg/L median (range)	6 (4–11)	7.5 (4–56)	7 (4–56)	–	–	*p* = 0.661						

ESR mm/h median (range)	17 (2–33)	26 (7–56)	22.5 (2–56)	–	–	*p* = 0.384						

*A. Early rheumatoid arthritis (RA) and healthy control cohorts’ demographics shown. Mann–Whitney *U* tests performed between the cohorts where applicable. B. Flow cytometry cell sorting was performed on 8 early RA patients and 4 healthy controls. The early RA patients were further split into 4 IGS+ and 4 IGS− patients. Respective demographics for each are shown*.

*^a^One-way ANOVA and ±Mann–Whitney U tests used*.

*Significant *p* values (<0.05) are shown in bold*.

*DAS-28, disease activity score-28 (ESR); EMS, early morning stiffness; eRA, early RA; HC, healthy controls; IGS, interferon gene signature; M:F ratio, male:female ratio; mins, minutes; SJC, swollen joint count; TJC, tender joint count; VAS, visual analog scale*.

### pDC, CD1c+, and CD141+ DC Peripheral Blood Frequency Is Reduced in Early RA, Which Is Sustained Into Established Disease

**Figure 1:**
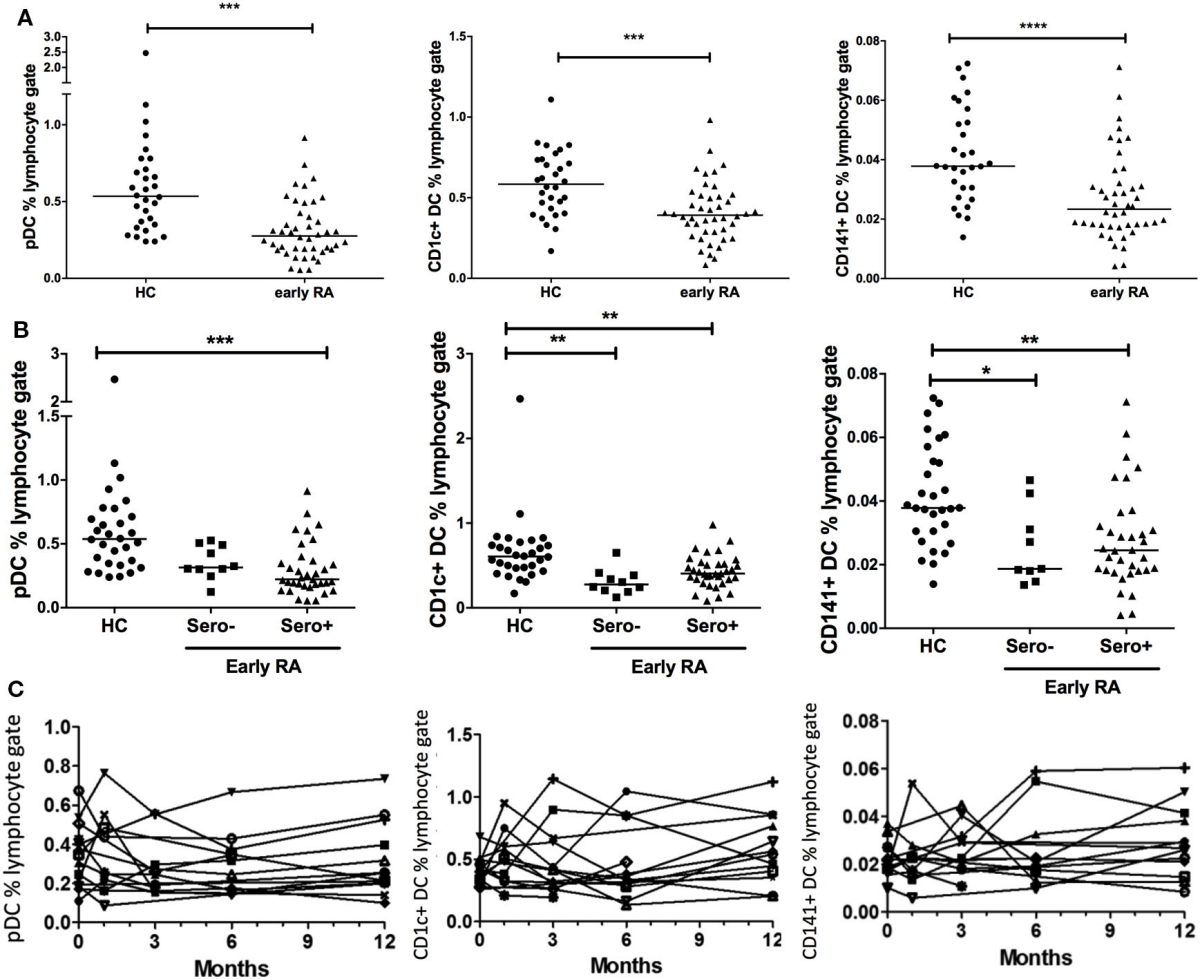
Peripheral blood DC frequency is reduced in early rheumatoid arthritis (RA). **(A)** Peripheral blood plasmacytoid DCs (pDCs), CD1c+, and CD141+ dendritic cells were identified by flow cytometry and recorded as a percentage of the circulating lymphocyte population in early RA patients (*n* = 44); and healthy controls (*n* = 30). Mann–Whitney *U* test. **(B)** The early RA cohort was further split into seropositive (RF+ and/or anti-CCP+) or seronegative (both RF+ and anti-CCP−). One way ANOVA with Tukey’s multiple comparison test. Horizontal lines depict median values. **(C)** pDC, CD1c+, and CD141+ DC frequencies were enumerated longitudinally in an early RA cohort (*n* = 15) at baseline and then 1, 3, 6, and 12 months after diagnosis. Each line represents an individual patient. Wilcoxon signed rank test. *p < 0.05, **p < 0.01, ***p < 0.001, ****p < 0.0001.

Plasmacytoid DCs, CD1c+, and CD141+ DC frequency was compared across disease cohorts. We also examined DC number in our early RA cohort in relation to the whole blood lymphocyte count (×10^9^/L). Equivalent data were not available for our healthy controls; however, there was a highly significant (*p* < 0.0001) positive association between DC frequency and DC number in early RA patients (Figure S2 in Supplementary Material). We, therefore, focused on DC frequency data, which were available for both cohorts. There was no effect of age or gender on DC subset frequencies (data not shown). All DC subsets had significantly reduced frequency in early RA compared with healthy controls (Figure 1A). When dividing the early RA cohort by serostatus, pDCs were significantly reduced in seropositive (either RF+ or anti-CCP+ or both) but not in seronegative (both RF− and anti-CCP−) early RA patients. CD1c+ and CD141+ DCs were significantly reduced in both seronegative and seropositive early RA patients compared with healthy controls (Figure 1B). There was no difference when examining RF or anti-CCP serostatus separately. Longitudinal DC frequency remained stable for all subsets from baseline during the 12 months after initiation of treatment (Figure 1C).

### CD1c+ DC but Not pDC or CD141+ DC Frequency Inversely Associates With Disease Activity; CD141+ DC Frequency Inversely Associates With the IGS

**Figure 2:**
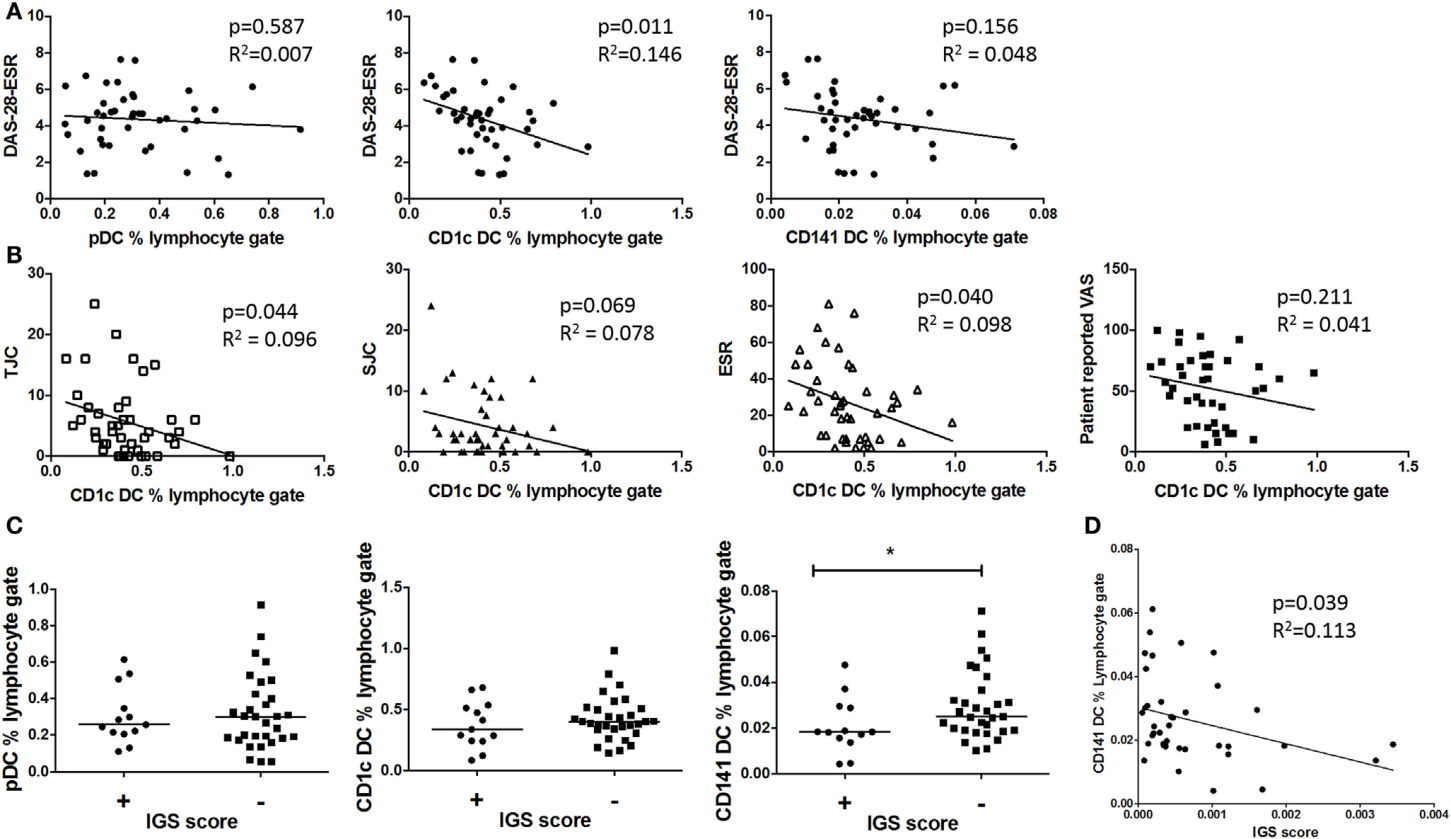
Only circulating CD1c+ DC frequencies inversely associate with disease activity and CD41+ DC but not pDC frequencies inversely associate with the interferon gene signature (IGS) **(A)** Plots depict linear regression of pDC, CD1c+ DC, and CD141+ DC frequency and disease activity (DAS-28-ESR) in early rheumatoid arthritis (RA) (*n* = 42). **(B)**: Individual DAS-28-ESR components are examined with relation to CD1c+ frequency (linear regression) **(C)** Early RA patients (*n* = 39) peripheral blood DC frequency divided by IGS (positive *n* = 13 and negative *n* = 26). Horizontal lines depict median values. Mann–Whitney *U* tests. **(D)** Linear regression of CD141+ DC frequency and IGS score. DAS-28, disease activity score 28; ESR, erythrocyte sedimentation rate; SJC, swollen joint count; TJC, tender joint count; VAS, visual analog scale.

Dendritic cell frequency was compared with early RA clinical phenotype. There was a significant inverse association between CD1c+ DC frequency and DAS-28-ESR, which was mainly driven by tender joint count and ESR; however, this was not seen with pDCs or CD141+ DCs (Figures 2A,B). Furthermore, there was no significant association between circulating cytokines IFN-γ, IL-6, IL-12 p70, TNF-α, IL-1β, IL-2, IL-13, IL-4, IL-10, and BAFF (data not shown) and DC frequency. This demonstrates that pDCs, CD1c+ DCs, and CD141+ DCs have a distinct relationship with disease activity, and this is independent of circulating pro-inflammatory cytokines. Due to pDCs’ marked IFN-I producing capacity and thus potential contribution to the IGS, we also compared DC frequency in IGS positive and negative early RA subtypes. There was no difference in either pDC or CD1c+ DC frequency between these early RA subtypes. However CD141+ DC frequency was significantly reduced in the IGS+ early RA cohort with a significant inverse association between CD141+ DC frequency and IGS score (Figures 2C,D).

### In Early RA cDC, but Not pDC, Have Increased Baseline CCR7 and CD86 Expression but for All DCs, Some Surface Markers of Cell Activation Fall With Disease Duration

**Figure 3:**
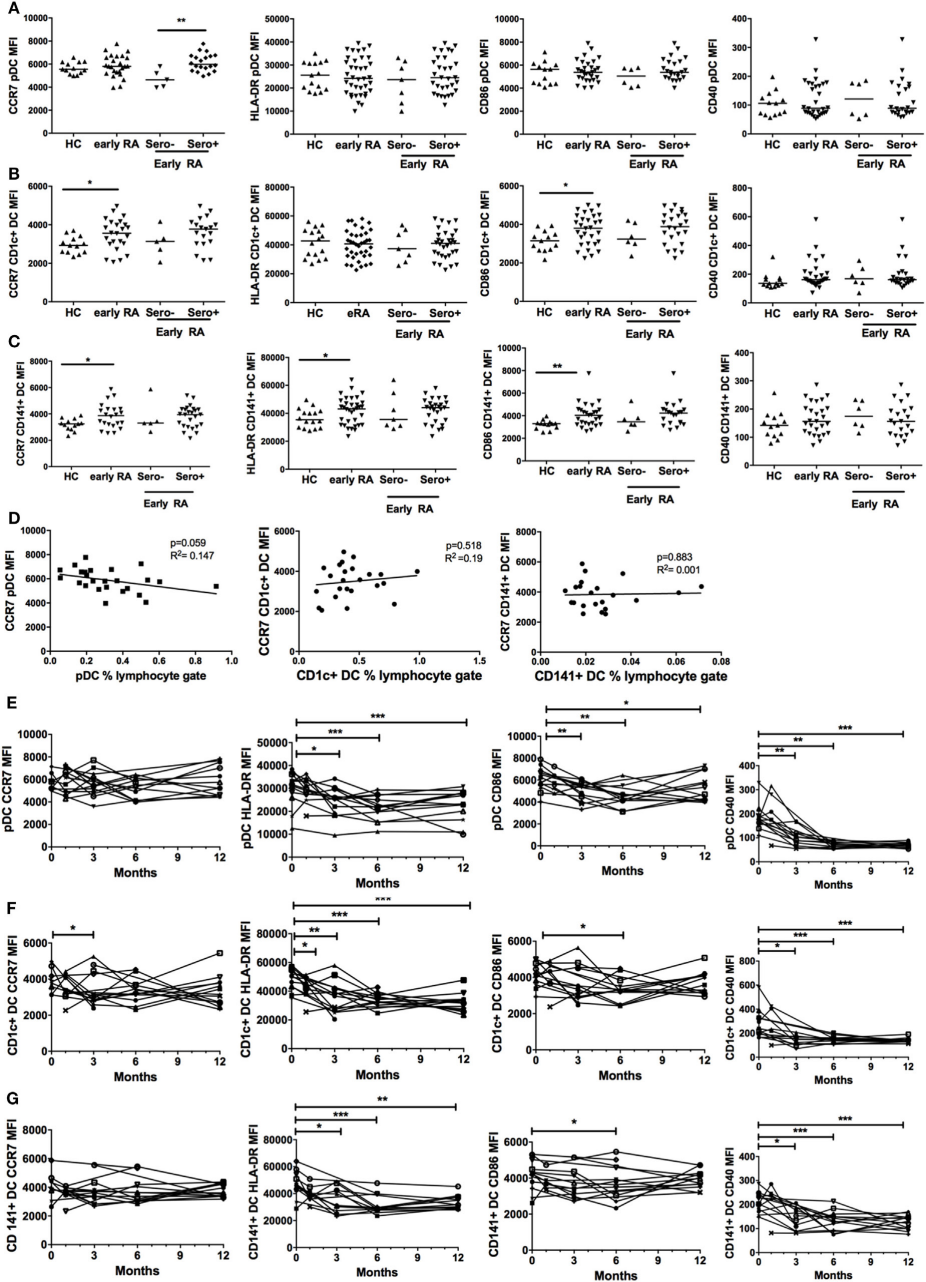
Plasmacytoid DCs (pDCs), CD1c+ DC, and CD141+ DC phenotype is different in early rheumatoid arthritis (RA) and changes with initiation of therapy. **(A)** pDC, **(B)** CD1c+ DC, and **(C)** CD141+ DC cell surface marker expression of CCR7, CD86, HLA-DR, and CD40 was quantified by flow cytometry [median fluorescence intensity (MFI)] in healthy controls (HC, *n* = 30) and early RA patients [*n* = 30–32; further split into seronegative (*n* = 5–7) and seropositive (*n* = 25) early RA]. Horizontal lines depict median values. Mann–Whitney *U* tests **(D)** pDC, CD1c+ DC, and CD141+ DC CCR7 MFI plotted (linear regression) against circulating DC frequency in all seropositive early RA patients (*n* = 25). **(E)** pDC, **(F)** CD1c+ DC cell, and **(G)** CD141+ DC surface marker expression was quantified in an early RA cohort (*n* = 15) at baseline and at 1, 3, 6, and 12 months after diagnosis. Each line represents an individual patient. Wilcoxon signed rank tests. *p < 0.05, **p < 0.01, ***p < 0.001.

We compared cell surface expression of CD40, CD86, HLA-DR, and CCR7 on DCs in early RA patients and healthy controls. These markers were chosen as they are implicated in DC maturation, such as antigen presentation and co-stimulation (CD40, HLA-DR, CD86) and DC migration (CCR7). There was no effect of age or gender on surface marker expression (data not shown). CD1c+ DCs and CD141+ DCs had significantly increased cell surface expression of CCR7 and CD86 in early RA compared with healthy controls and CD141+ DCs also had increased expression of HLA-DR but neither had any difference in CD40 expression. Serostatus did not appear to impact on surface marker expression (Figures 3B,C). pDC phenotype was comparable between disease and health (Figure 3A), but there was significantly increased CCR7 expression on seropositive compared with seronegative early RA pDCs. Given the association between CCR7 and lym-phocyte trafficking, we examined DC frequency and CCR7 expression in seropositive early RA patients. An inverse trend was seen for pDCs (*p* = 0.059) but not for cDCs (Figure 3D). Finally, there was no significant association between DC phenotype, disease activity, or the IGS (data not shown).

We subsequently studied pDC and cDC phenotypes in an early RA cohort longitudinally at baseline and at 1, 3, 6, and 12 months after diagnosis. There was a significant and sustained fall in pDC HLA-DR, CD40, and CD86 surface expression at 12 months but CCR7 expression remained static (Figure 3E). Both cDC subsets had significantly reduced HLA-DR and CD40 expression at 12 months but comparable CD86 and CCR7 expression (Figures 3F,G). Overall, these data suggest that, in early RA, both cDC subsets are relatively activated at baseline and for all DCs parameters of maturation fall as disease becomes established and treated.

### Healthy Control and Early RA Peripheral Blood pDCs Have Comparable IFN-α and IFN-λ Transcript Levels When Compared With Other Circulating Lymphocytes

**Figure 4:**
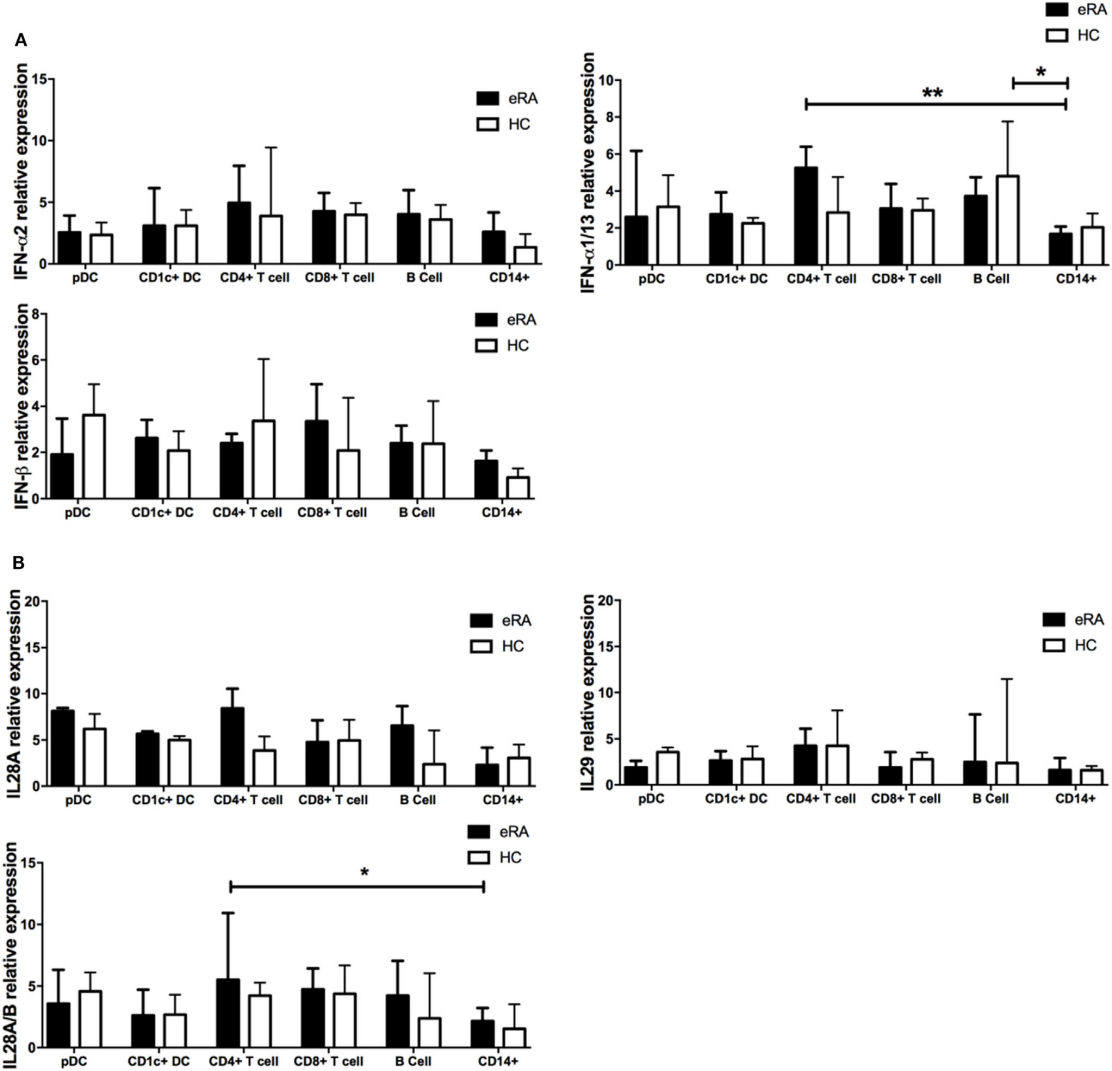
Type I, II, and III interferon mRNA transcripts in circulating lymphocyte subsets from early rheumatoid arthritis (RA) patients and healthy controls. **(A)** Type I IFN (IFN-α; *IFNA2, IFNA1/13, IFN*β) and **(B)** Type III IFN (*IFN-*λ*; IL18A, IL18A/B, IL29*) mRNA transcripts as determined by NanoString nCounter technologies was compared across flow cytometry cell sorted pDC, CD1c+ DC, CD8+ T cells, CD4+ T cells, B cells, and CD14+ monocytes from age and sex matched early RA patients (*n* = 8) and healthy controls (*n* = 4). One-way ANOVA with Tukey’s multiple comparison test. Horizontal lines depict medians with error bars of interquartile range. *p < 0.05, **p < 0.01.

Plasmacytoid DCs are the primary IFN-I producing cell subtype but the relative contribution of both IFN-I and pDCs to IGS generation in early RA is unclear. Since all cells can produce IFN-I and some suggest a role for both IFN-II and IFN-III in IGS generation (54, 55), we examined, in early RA and healthy control cohorts, type I, II, and III interferon transcript expression in pDCs as well as in CD1c+ DCs, B cells, CD14+ monocytes, and CD4+ and CD8+ T cells. We found that type I interferons *IFN-*α*2* and *IFN-*β*1* had comparable transcript expression between all the peripheral blood subsets. *IFN-*α*1/13* expression in CD14+ monocytes was significantly reduced when compared with B cells and CD4+ T cells, although expression between the other cell subsets was comparable (Figure 4A). Type III interferons (*IL-28, IL29*) again showed comparable expression across the cell subsets, although lower expression of *IL28A/B* was detected in monocytes when compared with CD4+ T cells (Figure 4B). These transcript levels were comparable to, or just above those seen for the negative controls on each nanostring chip emphasizing their negligible production. However, type II interferons (IFN-γ) were predictably and significantly raised in the T cell compartment with high transcript levels detected (median 23.86 transcript relative expression) but negligible expression in the other subsets (one-way ANOVA with Tukey’s *post hoc* analysis, data not shown). Given the role of IFN in generation of the IGS, we also compared the above transcripts after dividing the early RA co-hort by IGS (IGS+ vs IGS−). Unexpectedly IFN-I, IFN-II, and IFN-III transcript levels in all six lymphocyte subsets was comparable between IGS+ and IGS− early RA patients (one-way ANOVA with Tukey’s *post hoc* analysis, data not shown). Furthermore, for all lymphocyte subsets, linear regression did not demonstrate any significant association between the whole blood IGS score and IFN transcript level. Together, these data suggest that circulating pDCs do not account for IFN-I or IFN-III production in the circulation of early RA patients and thus may not underpin IGS generation. Finally, to compare cellular sensitivity to IFN and subsequent contribution to the IGS, the mean expression of five IRGs (*MxA, ISG15, OAS1, IFI6, IFI44L*) was examined in the above cell subsets in the IGS+ early RA cohort. There was no significant difference in expression between subsets (one-way ANOVA with Tukey’s *post hoc* analysis, data not shown).

### Differential Gene Expression Between RA Patients and Healthy Controls Observed for Both pDCs and CD1c+ DCs

To examine DCs in more depth, we examined the transcriptome of pDCs and CD1c+ DCs from early RA patients and compared these with age and sex-matched healthy controls (Full data set Table S3 in Supplementary Material). Data sets were interrogated for significantly DEGs between cohorts. We found that the transcriptome of DCs was significantly different in early RA with 12 pDC DEGs and 22 CD1c+ DC DEGs observed. Table 2 illustrates these DEGs.

**Table 2:** Differentially expressed genes between early rheumatoid arthritis (RA) and healthy control DCs.

Gene	Fold change	Adjusted p-value
**pDC: early RA vs healthy controls**		
CSF1R	2.55301	0.0187
PRDM1	2.51656	0.0229
IRF4	1.98403	0.0389
CD164	−1.52003	0.0111
IL6R	−1.60581	0.0251
MAPK14	−1.71980	0.0229
IFNAR1	−1.74364	0.0236
CD48	−1.81414	0.0024
IL6ST	−1.90833	0.0221
TNFSF4	−1.92215	0.0276
CCR5	−2.05663	0.0180
TNFRSF17	−2.14310	0.0024
**CD1c+ DC: early RA vs healthy controls**		
CDKN1A	2.56050	0.0040
IFITM1	2.54504	0.0411
NFIL3	2.24282	0.0046
BCL3	2.18094	0.0329
ICOSLG	2.17833	0.0329
MAPK14	−1.55133	0.0444
ALAS1	−1.56951	0.0448
IL6R	−1.57953	0.0081
CASP8	−1.59026	0.0323
TAGAP	−1.59159	0.0182
IKZF1	−1.59483	0.0046
IRF8	−1.59866	0.0042
TRAF5	−1.60970	0.0329
PSMB5	−1.66194	0.0291
ITGA5	−1.66465	0.0011
POLR1B	−1.73326	0.0329
FKBP5	−1.89383	0.0002
CASP3	−1.93814	0.0042
CCR2	−1.99854	0.0411
CCR6	−2.06347	0.0042
TNF	−2.07198	0.0081
BTLA	−2.24360	0.0042

*Plasmacytoid DCs (pDCs) and CD1c+ DCs were flow cytometry sorted from early RA patients (*n* = 8) and age- and sex-matched healthy controls (*n* = 4). pDC and CD1c+ DC RNA was isolated and approximately 600 immunology-related genes’ transcripts were analyzed using NanoString nCounter technology. Genes achieved differential expression significance when adjusted *p*-value < 0.05 (FDR corrected) and fold change >1.5*.

To seek differentially regulated specific pathways, we used *IPA*^®^, a powerful analysis tool for the integration and interpretation of transcriptomics data. This demonstrated increased involvement of pathways linked to proliferation/expansion in early RA pDCs (*p* = 1 × 10^−9^, *Z*-score 2.17, genes involved: CSF1R, IFNAR1, IL6R, IL6ST, MAPK14) and CD1c+ DCs (*p* = 1.77 × 10^−11^, *Z*-score 2.76, genes involved BCL3, BTLA, CCR6, ICOSLG, IKZF1, IL6R, IRF8, TNF), although this was more marked in CD1c+ DCs where they also showed reduced apoptosis (*p* = 2.09 × 10^−9^, *Z*-score 2.27, genes involved: BCL3, CASP3, CASP8, CDKN1A, ICOSLG, IL6R, IRF8, MAPK14, TNF).

When examining individual DEGs, pDCs in early RA had markedly upregulated *CSF1R* and *PRDM1* transcripts (fold changes > 2.5) and downregulated *TNFRSF17* (fold change > 2). Processes linked to inflammatory cytokine signaling were also differentially expressed, such as reduced *IFNAR1* and *IL6R*. CD1c+ DC DEGs also had reduced *IL6R* as well as highly increased *CDKN1A* transcript (fold change +2.56) whereas *BTLA*, B, and T lymphocyte attenuator, was the most reduced (fold change −2.24).

## Discussion

We report for the first time an extensive examination of peripheral blood pDCs, CD1c+ DCs, and CD141+ DCs in early, drug naïve RA patients including the transcriptomic analysis of 600+ immunology-related genes in pDCs and CD1c+ DCs. Our data suggest distinct roles for DC subsets in early RA pathogenesis, an understanding of which may have important therapeutic implications.

Most investigations of the pathogenic role of pDCs in autoimmunity are focused on their marked IFN-I-producing potential and its impact on B cell function. Indeed, sustained IFN-α production from RA pDCs was sufficient to induce autoantibodies *in vivo* (29), and there is a high IGS in established RA synovial fluid (38, 56). We previously showed an increased IGS in early RA (44) and, when examining pDC and CD1c+ DC transcriptomes, we found increased expression of IRG, such as *IFITMI* or *IRF4*, supporting this observation. IFN-α has been shown to reduce IL6R expression in some cell lines (57), which is in keeping with our observation of reduced *IL6R* in both pDC and CD1c+ DC subsets. Furthermore, pDCs had reduced *IFNAR1* expression, which is reduced upon ligation (58), again suggesting increased IFN-I signaling in the early RA cohort. However, when examining the relative contribution of each lymphocyte subset to the IGS as determined by five IRGs, there was no obvious subset where upregulation of these transcripts dominated. A previous study in RA examining three IRGs suggested that granulocytes may be the major contributor of the IGS although DCs were not examined in that study (59). There are thousands of potential IRGs, however (60), and their upregulation may be both ligand- and cell-dependent (61). Potentially, we did not examine the optimal IRGs to dissect a cell specific effect of IFN-I signaling or exposure.

Despite their IFN-I producing potential, we did not see association between peripheral blood pDC phenotype/frequency and the IGS in early RA. Type III interferons (IFN-III or IFN-λ) are also produced by pDCs and promote upregulation of genes normally associated with response to IFN-I (54, 62, 63). Furthermore, IFN-III are increased in RA and can associate with disease-specific antibodies (64, 65). However, pDCs had comparable IFN-I and IFN-III transcript levels to those in other circulating lymphocyte subsets, and these levels were independent of the background IGS. There are 13 IFN-α subtypes and expression of both IFN-α and IFN-λ subtypes are cell- and ligand-dependent (66), which may have contributed to these unexpected results, since we only examined two IFN-α transcripts (*IFN-*α*2* and *IFN-*α*1/13*). Nonetheless, these data suggest that in early RA, circulating pDCs are not uniquely responsible for whole blood IGS generation by either IFN-I or IFN-III. Generation of IFN-I/III by pDCs may instead be primarily tissue-based, thereby highlighting the potential importance of migration and subsequent microenvironment on DC function. This is supported by the literature where pDCs are present in affected tissue, such as lupus nephritis or salivary tissue in Sjogren’s syndrome, where they locally produce IFN-I (67).

Unexpectedly, there was an inverse association between CD141+ DC frequency and the IGS, which was independent of CD141+ DC phenotype and circulating cytokines. This is the first time that CD141+ DCs have been examined in the circulation of early RA patients, nonetheless, CD141+ DCs are not recognized for their IFN-I production. They do, however, produce IFN-II upon stimulation (68) as well as large amounts of interferon-λ (IFN-III) following stimulation with TLR3 ligands (69–71). In SLE, there has been a suggestion that there is a large IFN-II component to the IGS (55) and, as previously discussed, IFN-III can upregulate IRGs normally associated with IFN-I exposure (54). DC activation and IFN production may also upregulate chemokine receptors resulting in trafficking of the CD141+ DCs out of the peripheral circulation. This all raises the possibility that CD141+ DCs may play an important role in RA IGS generation.

Plasmacytoid DCs and CD1c+ DCs are known to be reduced in the peripheral circulation of established RA patients, particularly in those with active disease, where they migrate to the synovial compartment (25–27, 29, 31, 35, 38, 72). CD141+ DC circulating frequency in RA has not been previously compared with healthy controls, but we have demonstrated a reduced frequency of all DC subsets in the circulation of early, drug-naïve RA patients, which was sustained up to 12 months. This emphasizes early involvement of these DC subsets in disease pathogenesis in keeping with previous observations in animal arthritis models where lymphatic CD1c+ DCs are important in breaching tolerance (24). Indeed, both CD1c+ DC and pDC transcriptomic analysis suggested increased DC proliferation despite their reduced frequency in the blood, supporting the suggestion of DC migration. Moreover, there was a further reduced pDC frequency in seropositive RA patients and these pDCs also had higher CCR7 expression, a key chemokine in promoting pDC migration (73, 74), which inversely correlated with pDC frequency. While CCR7 expression classically causes lymph node migration, its ligand, CCL19/CCL21, is significantly increased in the synovial fluid of RA patients (75) supporting the suggestion of early synovial pDC migration sustained into established RA. In keeping with our observations, seropositive established RA patients have a higher frequency of synovial fluid pDCs than seronegative patients, which co-localize with B-cells in synovial tissue (26). Synovial pDCs produce IFN-I (26) and we, therefore, propose that CCR7-driven migration of pDC into the synovial compartment in seropositive RA patients precedes localized IFN-I production and, potentially, consequent autoantibody generation. This mirrors what is known about intra-articular CD1c+ DC function where the microenvironment modifies DCs and subsequent T cell activation, in a manner that is not necessarily observed in the periphery (17, 20–22).

There were distinct differences in how the DC subsets associated with disease activity. pDCs demonstrated a comparatively immature phenotype in early RA and their frequency did not associate with disease activity, supporting what has been reported in established RA (26, 29). In contrast, both CD1c+ DCs and CD141+ DCs had an activated phenotype although only CD1c+ DCs showed an association with disease activity, a relationship again replicated in established RA (29). These observations were further corroborated by the transcriptional data, which highlighted the potential tolerizing potential of pDCs that is being increasingly appreciated in other disease states (76). Early RA pDCs were characterized by striking downregulation of *TNFRSF17* with upregulation of *PRDM1* and *CSF1R. TNFRSF17 (BMCA)* is increased upon TLR engagement (77) and is expressed at high levels on pDCs from multiple myeloma patients, where there is pathogenic expansion of plasma cells (78). Reduced expression could, therefore, be predicted to have immunoregulatory consequences. *PRDM1 (BLIMP1)* is increased in human pDCs in response to IFN-α and has been proposed as a mechanism to negatively control the production of effector cytokines, thereby skewing toward pDC tolerance (79). Notably, *CD27* expression was minimal in the pDC cohort and markedly reduced compared with B cells, excluding plasma cell contamination. *CSF1R* is a tyrosine kinase receptor that causes pDC differentiation and proliferation (80–82). This pathway is also believed to be important in inflammatory macrophage differentiation. Blockers of CSF1R/CSF1 have been trialed as a potential therapy for RA (83) although the only completed phase 2 clinical trial reported little clinical benefit (84). Potentially, these therapies could also affect pDC proliferation and thus abrogate any tolerogenic function, thereby compromising clinical outcomes. In contrast, when compared with healthy controls, the most differentially increased genes in early RA CD1c+ DCs were upregulated *CKDN1a* and downregulated *BTLA*. C*KDN1a* is associated with an inflammatory and potentially pathogenic phenotype in mouse DCs (18, 85). *BTLA* is important in DC skewing of T cells toward Tregs (86) and reduced expression has been linked to an inflammatory, potentially pathogenic, monocyte-derived DC subset in RA (21, 22).

These findings are in agreement with current literature where CD1c+ DCs are likely to play a pathogenic role in RA (19, 20, 23); however, the role of pDCs in RA to date has been frequently contradictory. On the one hand, pDCs from RA synovial fluid activate T cells and trigger the production of pro-inflammatory cytokines (38), and intra-articular transfer of activated pDCs propagated an inflammatory arthritis phenotype in mice (51). On the contrary, mature peripheral blood pDCs from RA patients promoted differentiation of naive T cells into IL-10-secreting Tregs (35). Furthermore, depletion of pDCs in a RA model resulted in breach of tolerance with development of autoantibodies (36) and in various inflammatory arthritis models enhanced pDC recruitment and activation to the arthritic joints significantly eased arthritis, consistent with an anti-inflammatory or tolerogenic role (37). Our data go some way to resolving these apparently conflicting views, emphasizing the importance of anatomical microenvironment and disease stage on pDC function. Indeed, pathological processes in early RA are known to be distinct from those that dominate later (87, 88), and we observed a significant change in pDC, CD1c+, and CD141+ DC phenotype after 12 months of disease. This is in keeping with published data where established RA patients (on immunomodulatory therapy) had reduced pDC maturation compared with healthy controls (28, 31).

Finally, the understanding of the role of CD141+ DCs in RA has been mainly derived from mouse equivalent DCs (CD8+ CD103+ CD11b− DCs) (89, 90), which accelerated the onset of CIA when adoptively transferred with CD4+ T cells (34). They have been examined once in established RA where numbers were enriched in the synovial compartment and demonstrated an activated phenotype (33). This supports our observation of reduced CD141+ DC frequency in blood, with increased HLA-DR and CD80 expression. This suggests that CD141+ DCs, despite their relatively small numbers when compared with other cellular subsets, may contribute to disease pathogenesis.

In conclusion, we simultaneously examined, for the first time in early RA, pDCs, CD1c+ DCs, and CD141+ DCs. While all DC subsets are reduced, CD141 +DC, but not pDC, frequency inversely correlated with the IGS and may be a hitherto unappreciated source of IFN-I. Furthermore, pDCs have similar levels of IFN-I and IFN-III mRNA transcripts as other major leukocyte subsets. Additional marked differences exist between the DC subsets with regards to activation and relationship with disease activity. These hint that pDCs may have, in the peripheral circulation at least, a more tolerogenic role when compared with CD1c+ DCs in early disease. Future work is, therefore, justified to compare synovial pDCs and cDCs with those in the circulation to further elucidate the effect of microenvironment and disease duration on DC function and, potentially, to expose novel therapeutic avenues and targets.

## Ethics Statement

This study was carried out in accordance with the recommendations of North East—Newcastle & North Tyneside 2 Research Ethics Committee with written informed consent from all subjects. All subjects gave written informed consent in accordance with the Declaration of Helsinki. The protocol was approved by the North East—Newcastle & North Tyneside 2 Research Ethics Committee (REC reference: 12/NE/0251).

## Author Contributions

FC performed all experiments, was involved in project development, and wrote the first draft of the manuscript. AS performed bioinformatics analysis. AA and CH assisted in experimental design. AP assisted with patient recruitment. IM and MK-S advised on manuscript structure and DC biology. JI was integral in project development and writing the manuscript. All authors reviewed manuscript content prior to submission.

## Conflict of Interest Statement

There are no conflicts of interest. The submitted work was performed without of any personal, professional, or financial relationships that could potentially be construed as a conflict of interest.

## Supplementary Material

Figure S1 Plasmacytoid DCs (pDCs), CD1c+ dendritic cells (DCs), and CD141+ DCs flow cytometry gating strategy. Gating strategy is shown to identify DCs from isolated PBMCs. pDCs, CD1c+ DCs, and CD141+ DCs were defined as CD19−CD20−CD3−CD203c−HLA-DR+CD14−CD1c−CD141− or ^dim^CD11c− CD123, CD19−CD20−CD3−CD203c−HLA-DR+CD14−CD1c+CD141− or ^dim^CD11c+ and CD19−CD20−CD3−CD203c−HLA-DR+CD14−CD1c+CD141^high^CD11c−, respectively.

Figure S2 Plasmacytoid DC (pDC), CD1c+ DC, and CD141+ DC number and frequency in early RA whole blood. Plots depict early RA DC number (×10^6^/L) and frequency (%) in the whole blood for **(A)** pDCs, **(B)** CD1c+ DCs, and **(C)** CD141+ DCs. Linear regression.
