# Supplementary material for: Phenotypic and Transcriptomic Analysis of Peripheral Blood Plasmacytoid and Conventional Dendritic Cells in Early Drug Naïve Rheumatoid Arthritis
Source: Front Immunol. 2018 May 9;9:755. doi: 10.3389/fimmu.2018.00755 (PMC5968398; doi:10.3389/fimmu.2018.00755)
Supplement: Supplementary file 1 [file Table_1.DOCX]

| **Gene** | **Primer** | | **Roche Universal Probe Library Number** |
| --- | --- | --- | --- |
|  | **Forward** | **Reverse** |  |
| ***MxA*** | GGAGAACATGGTGTGATAATCCT | CACCGTGACACTGGGATTC | 83 |
| ***ISG15*** | GCGAACTCATCTTTGCCAGTA | CCAGCATCTTCACCGTCAG | 23 |
| ***OAS1*** | CATCCGCCTAGTCAAGCACT | CAGGAGCTCCAGGGCATAC | 87 |
| ***IFI6*** | CGGGCTGAAGATTGCTTCT | AAAGCGATACCGCCTTCTG | 25 |
| ***IFI44L*** | TGACACTATGGGGCTAGATGG | GAATGCTCAGGTGTAATTGGTTT | 15 |

**Supplementary table S1: RT-PCT**

Forward and reverse gene specific primer sequences used for RT-PCR and probe number from Roche universal probe library.
