# Supplementary material for: Phenotypic and Transcriptomic Analysis of Peripheral Blood Plasmacytoid and Conventional Dendritic Cells in Early Drug Naïve Rheumatoid Arthritis
Source: Front Immunol. 2018 May 9;9:755. doi: 10.3389/fimmu.2018.00755 (PMC5968398; doi:10.3389/fimmu.2018.00755)
Supplement: Supplementary file 2 [file Table_2.docx]

| *ABCB1* | *C6* | *CD164* | *CD96* | *CXCL11* |
| --- | --- | --- | --- | --- |
| *ABL1* | *C7* | *CD19* | *CD97* | *CXCL12* |
| *ADA* | *C8A* | *CD1A* | *CD99* | *CXCL13* |
| *AHR* | *C8B* | *CD1D* | *CDH5* | *CXCL2* |
| *AICDA* | *C8G* | *CD2* | *CDKN1A* | *CYBB* |
| *AIRE* | *C9* | *CD209* | *CEACAM1* | *DEFB1* |
| *APP* | *CAMP* | *CD22* | *CEACAM6* | *DEFB103A* |
| *ARG1* | *CARD9* | *CD24* | *CEACAM8* | *DEFB103B* |
| *ARG2* | *CASP1* | *CD244* | *CEBPB* | *DEFB4A* |
| *ARHGDIB* | *CASP10* | *CD247* | *CFB* | *DPP4* |
| *ATG10* | *CASP2* | *CD27* | *CFD* | *DUSP4* |
| *ATG12* | *CASP3* | *CD274* | *CFH* | *EBI3* |
| *ATG16L1* | *CASP8* | *CD276* | *CFI* | *EDNRB* |
| *ATG5* | *CCBP2* | *CD28* | *CFP* | *EGR1* |
| *ATG7* | *CCL11* | *CD34* | *CHUK* | *EGR2* |
| *ATM* | *CCL13* | *CD36* | *CIITA* | *ENTPD1* |
| *B2M* | *CCL15* | *CD3D* | *CISH* | *EOMES* |
| *B3GAT1* | *CCL16* | *CD3E* | *CLEC4A* | *ETS1* |
| *BATF* | *CCL18* | *CD3EAP* | *CLEC4E* | *FADD* |
| *BATF3* | *CCL19* | *CD4* | *CLEC5A* | *FAS* |
| *BAX* | *CCL2* | *CD40* | *CLEC6A* | *FCAR* |
| *BCAP31* | *CCL20* | *CD40LG* | *CLEC7A* | *FCER1A* |
| *BCL10* | *CCL22* | *CD44* | *CLU* | *FCER1G* |
| *BCL2* | *CCL23* | *CD46* | *CMKLR1* | *FCGR1A/B* |
| *BCL2L11* | *CCL24* | *CD48* | *CR1* | *FCGR2A* |
| *BCL3* | *CCL26* | *CD5* | *CR2* | *FCGR2A/C* |
| *BCL6* | *CCL3* | *CD53* | *CRADD* | *FCGR2B* |
| *BID* | *CCL4* | *CD55* | *CSF1* | *FCGR3A/B* |
| *BLNK* | *CCL5* | *CD58* | *CSF1R* | *FCGRT* |
| *BST1* | *CCL7* | *CD59* | *CSF2* | *FKBP5* |
| *BST2* | *CCL8* | *CD6* | *CSF2RB* | *FN1* |
| *BTK* | *CCND3* | *CD7* | *CSF3R* | *FOXP3* |
| *BTLA* | *CCR1* | *CD70* | *CTLA4_all (common probe)* | *FYN* |
| *C14orf166* | *CCR10* | *CD74* | *CTLA4-TM (membrane-bound form)* | *GATA3* |
| *C1QA* | *CCR2* | *CD79A* | *sCTLA4 (soluble form)* | *GBP1* |
| *C1QB* | *CCR5* | *CD79B* | *CTNNB1* | *GBP5* |
| *C1QBP* | *CCR6* | *CD80* | *CTSC* | *GFI1* |
| *C1R* | *CCR7* | *CD81* | *CTSG* | *GNLY* |
| *C1S* | *CCR8* | *CD82* | *CTSS* | *GP1BB* |
| *C2* | *CCRL1* | *CD83* | *CUL9* | *GPI* |
| *C3* | *CCRL2* | *CD86* | *CX3CL1* | *GPR183* |
| *C4A/B* | *CD14* | *CD8A* | *CX3CR1* | *GZMA* |
| *C4BPA* | *CD160* | *CD8B* | *CXCL1* | *GZMB* |
| *C5* | *CD163* | *CD9* | *CXCL10* | *GZMK* |

| *HAMP* | *IKZF3* | *IL4* | *KIR3DL3* | *MAP4K4* |
| --- | --- | --- | --- | --- |
| *HAVCR2* | *IL10* | *IL4R* | *KIT* | *MAPK1* |
| *HFE* | *IL10RA* | *IL5* | *KLRAP1* | *MAPK11* |
| *HLA-A* | *IL11RA* | *IL6* | *KLRB1* | *MAPK14* |
| *HLA-B* | *IL12A* | *IL6R* | *KLRC1* | *MAPKAPK2* |
| *HLA-C* | *IL12B* | *IL6ST* | *KLRC2* | *MARCO* |
| *HLA-DMA* | *IL12RB1* | *IL7* | *KLRC3* | *MASP1* |
| *HLA-DMB* | *IL13* | *IL7R* | *KLRC4* | *MASP2* |
| *HLA-DOB* | *IL13RA1* | *IL8* | *KLRD1* | *MBL2* |
| *HLA-DPA1* | *IL15* | *IL9* | *KLRF1* | *MBP* |
| *HLA-DPB1* | *IL16* | *ILF3* | *KLRF2* | *MCL1* |
| *HLA-DQA1* | *IL17A* | *IRAK1* | *KLRG1* | *MIF* |
| *HLA-DQB1* | *IL17B* | *IRAK2* | *KLRG2* | *MME* |
| *HLA-DRA* | *IL17F* | *IRAK3* | *KLRK1* | *MR1* |
| *HLA-DRB1* | *IL18* | *IRAK4* | *LAG3* | *MRC1* |
| *HLA-DRB3* | *IL18R1* | *IRF1* | *LAIR1* | *MS4A1* |
| *HRAS* | *IL18RAP* | *IRF3* | *LAMP3* | *MSR1* |
| *ICAM1* | *IL19* | *IRF4* | *LCK* | *MUC1* |
| *ICAM2* | *IL1A* | *IRF5* | *LCP2* | *MX1* |
| *ICAM3* | *IL1B* | *IRF7* | *LEF1* | *MYD88* |
| *ICAM4* | *IL1R1* | *IRF8* | *LGALS3* | *NCAM1* |
| *ICAM5* | *IL1R2* | *IRGM* | *LIF* | *NCF4* |
| *ICOS* | *IL1RAP* | *ITGA2B* | *LILRA1* | *NCR1* |
| *ICOSLG* | *IL1RL1* | *ITGA4* | *LILRA2* | *NFATC1* |
| *IDO1* | *IL1RL2* | *ITGA5* | *LILRA3* | *NFATC2* |
| *IFI16* | *IL1RN* | *ITGA6* | *LILRA4* | *NFATC3* |
| *IFI35* | *IL2* | *ITGAE* | *LILRA5* | *NFIL3* |
| *IFIH1* | *IL20* | *ITGAL* | *LILRA6* | *NFKB1* |
| *IFIT2* | *IL21* | *ITGAM* | *LILRB1* | *NFKB2* |
| *IFITM1* | *IL21R* | *ITGAX* | *LILRB2* | *NFKBIA* |
| *IFNA1/13* | *IL22* | *ITGB1* | *LILRB3* | *NFKBIZ* |
| *IFNA2* | *IL22RA2* | *ITGB2* | *LILRB4* | *NLRP3* |
| *IFNAR1* | *IL23A* | *ITLN1* | *LILRB5* | *NOD1* |
| *IFNAR2* | *IL23R* | *ITLN2* | *LITAF* | *NOD2* |
| *IFNB1* | *IL26* | *JAK1* | *LTA* | *NOS2* |
| *IFNG* | *IL27* | *JAK2* | *LTB4R* | *NOTCH1* |
| *IFNGR1* | *IL28A* | *JAK3* | *LTB4R2* | *NOTCH2* |
| *IGF2R* | *IL28A/B* | *KCNJ2* | *LTBR* | *NT5E* |
| *IKBKAP* | *IL29* | *KIR_Activating_ Subgroup_1* | *LTF* | *PAX5* |
| *IKBKB* | *IL2RA* | *KIR_Activating_ Subgroup_2* | *LY96* | *PDCD1* |
| *IKBKE* | *IL2RB* | *KIR_Inhibiting_ Subgroup_1* | *MAF* | *PDCD1LG2* |
| *IKBKG* | *IL2RG* | *KIR_Inhibiting_ Subgroup_2* | *MALT1* | *PDCD2* |
| *IKZF1* | *IL3* | *KIR3DL1* | *MAP4K1* | *PDGFB* |
| *IKZF2* | *IL32* | *KIR3DL2* | *MAP4K2* | *PDGFRB* |

| *PECAM1* | *SELL* | *TLR3* | *ZEB1* |
| --- | --- | --- | --- |
| *PIGR* | *SELPLG* | *TLR4* |  |
| *PLA2G2A* | *SERPING1* | *TLR5* | ***Customised additional genes:*** |
| *PLA2G2E* | *SH2D1A* | *TLR7* | *ISG15* |
| *PLAU* | *SIGIRR* | *TLR8* | *DDX58 (RIG-1)* |
| *PLAUR* | *SKI* | *TLR9* | *IDO2* |
| *PML* | *SLAMF1* | *TMEM173* | *STING* |
| *POU2F2* | *SLAMF6* | *TNF* | *IRF9* |
| *PPARG* | *SLAMF7* | *TNFAIP3* | *cGAS* |
| *PPBP* | *SLC2A1* | *TNFAIP6* | *MAVS* |
| *PRDM1* | *SMAD3* | *TNFRSF10C* | *CCR9* |
| *PRF1* | *SMAD5* | *TNFRSF11A* | *IFI6* |
| *PRKCD* | *SOCS1* | *TNFRSF13B* | *IFI44L* |
| *PSMB10* | *SOCS3* | *TNFRSF13C* | *OAS1* |
| *PSMB5* | *SPP1* | *TNFRSF14* | *TREX1* |
| *PSMB7* | *SRC* | *TNFRSF17* | *RNaseH2* |
| *PSMB8* | *STAT1* | *TNFRSF1B* | *SAMHD1* |
| *PSMB9* | *STAT2* | *TNFRSF4* |  |
| *PSMC2* | *STAT3* | *TNFRSF8* |  |
| *PSMD7* | *STAT4* | *TNFRSF9* |  |
| *PTAFR* | *STAT5A* | *TNFSF10* |  |
| *PTGER4* | *STAT5B* | *TNFSF11* |  |
| *PTGS2* | *STAT6* | *TNFSF12* |  |
| *PTK2* | *SYK* | *TNFSF13B* |  |
| *PTPN2* | *TAGAP* | *TNFSF15* |  |
| *PTPN22* | *TAL1* | *TNFSF4* |  |
| *PTPN6* | *TAP1* | *TNFSF8* |  |
| *PTPRC_all (common probe)* | *TAP2* | *TOLLIP* |  |
| *CD45R0* | *TAPBP* | *TP53* |  |
| *CD45RA* | *TBK1* | *TRAF1* |  |
| *CD45RB* | *TBX21* | *TRAF2* |  |
| *PYCARD* | *TCF4* | *TRAF3* |  |
| *RAF1* | *TCF7* | *TRAF4* |  |
| *RAG1* | *TFRC* | *TRAF5* |  |
| *RAG2* | *TGFB1* | *TRAF6* |  |
| *RARRES3* | *TGFBI* | *TYK2* |  |
| *RELA* | *TGFBR1* | *UBE2L3* |  |
| *RELB* | *TGFBR2* | *VCAM1* |  |
| *RORC* | *THY1* | *VTN* |  |
| *RUNX1* | *TICAM1* | *XBP1* |  |
| *S100A8* | *TIGIT* | *XCL1* |  |
| *S100A9* | *TIRAP* | *XCR1* |  |
| *S1PR1* | *TLR1* | *ZAP70* |  |
| *SELE* | *TLR2* | *ZBTB16* |  |

**Supplementary table S3:** Full list of genes included on NanoString nCounter Human Immunology V2 Panel and additional 14 customised genes examined.
