# Supplementary material for: Phenotypic and Transcriptomic Analysis of Peripheral Blood Plasmacytoid and Conventional Dendritic Cells in Early Drug Naïve Rheumatoid Arthritis
Source: Front Immunol. 2018 May 9;9:755. doi: 10.3389/fimmu.2018.00755 (PMC5968398; doi:10.3389/fimmu.2018.00755)
Supplement: Supplementary file 3 [file Table_3.docx]

|  | **Plasmacytoid dendritic cells** | | | | | | | | | | | | **CD1c+ dendritic cells** | | | | | | | | | | | |
| --- | --- | --- | --- | --- | --- | --- | --- | --- | --- | --- | --- | --- | --- | --- | --- | --- | --- | --- | --- | --- | --- | --- | --- | --- |
| **Gene** | **Early RA patients** | | | | | | | | **Healthy Controls** | | | | **Early RA patients** | | | | | | | | **Healthy controls** | | | |
| **ABCB1** | 5.303533 | 5.122719 | 14.07933 | 2.399412 | 10.50385 | 7.122289 | 6.175141 | 12.25383 | 7.650661 | 8.154897 | 5.970447 | 7.325656 | 3.831916 | 2.983631 | 3.151463 | 7.825493 | 14.8448 | 8.974105 | 3.993646 | 4.976272 | 4.015721 | 6.69361 | 3.517743 | 4.400786 |
| **ABL1** | 24.39625 | 33.80995 | 36.95825 | 44.38913 | 24.15885 | 24.92801 | 45.28436 | 33.08533 | 35.47124 | 25.9474 | 37.52852 | 28.48866 | 19.15958 | 23.27232 | 22.06024 | 26.60668 | 19.48381 | 23.75498 | 27.95553 | 20.80986 | 21.5845 | 22.6553 | 30.34053 | 29.20521 |
| **ADA** | 221.6877 | 103.4789 | 142.5533 | 190.7533 | 163.86 | 234.1453 | 312.8738 | 162.9759 | 201.6992 | 262.4394 | 265.2584 | 327.2126 | 35.1259 | 26.85268 | 27.5753 | 23.99818 | 28.76181 | 30.08965 | 41.53392 | 28.04808 | 35.63953 | 32.43827 | 29.4611 | 29.20521 |
| **AHR** | 112.4349 | 117.8225 | 89.75575 | 88.77826 | 115.5423 | 108.6149 | 169.8164 | 138.4682 | 125.1926 | 134.9265 | 120.2619 | 143.2573 | 823.2232 | 486.9285 | 602.7174 | 801.8522 | 684.7166 | 536.3348 | 996.8142 | 837.8232 | 941.6867 | 944.8289 | 1181.962 | 1359.443 |
| **AICDA** | 4.242826 | 5.122719 | 7.039667 | 5.998531 | 6.30231 | 3.561145 | 3.08757 | 2.450765 | 4.868602 | 3.706772 | 4.264605 | 5.697732 | 3.831916 | 2.983631 | 5.515061 | 1.565099 | 6.494602 | 4.223108 | 1.597459 | 7.238213 | 2.007861 | 4.119145 | 3.517743 | 2.000357 |
| **AIRE** | 4.242826 | 11.26998 | 14.07933 | 7.198237 | 11.55423 | 8.012575 | 10.2919 | 9.803062 | 7.650661 | 5.930835 | 6.823368 | 12.20943 | 7.025179 | 8.354166 | 11.81799 | 6.260394 | 7.422402 | 5.278885 | 8.786022 | 6.785825 | 9.035373 | 8.753183 | 5.276615 | 8.4015 |
| **APP** | 2273.094 | 1945.609 | 1520.568 | 2837.305 | 1435.876 | 1963.971 | 2590.471 | 2244.901 | 2101.15 | 1813.353 | 2148.508 | 2282.349 | 165.411 | 128.8928 | 107.1498 | 154.4231 | 79.79082 | 145.1694 | 125.7999 | 151.0977 | 132.5188 | 127.6935 | 163.575 | 168.03 |
| **ARG1** | 6.364239 | 3.073631 | 3.519833 | 1.199706 | 3.151155 | 1.780572 | 2.05838 | 3.676148 | 0.695515 | 2.965417 | 0.852921 | 4.069809 | 3.193263 | 2.386905 | 2.363597 | 3.130197 | 0.9278 | 2.111554 | 1.198094 | 2.261942 | 4.015721 | 2.059572 | 0.439718 | 0.800143 |
| **ARG2** | 6.364239 | 3.073631 | 3.519833 | 1.199706 | 4.20154 | 2.670858 | 2.05838 | 2.450765 | 4.868602 | 2.965417 | 0.852921 | 0.813962 | 1.277305 | 3.580357 | 2.363597 | 3.130197 | 2.783401 | 4.223108 | 1.198094 | 4.523883 | 2.509826 | 3.089359 | 0.439718 | 3.200571 |
| **ARHGDIB** | 522.9283 | 449.7747 | 395.9813 | 519.4728 | 364.4836 | 453.1557 | 594.8719 | 553.873 | 447.2159 | 419.6065 | 561.222 | 491.6329 | 1012.903 | 781.1145 | 766.5935 | 861.8476 | 692.139 | 913.7751 | 1015.584 | 986.6589 | 902.5334 | 868.1098 | 964.741 | 1007.38 |
| **ATG10** | 54.09603 | 39.95721 | 47.51775 | 63.58443 | 49.36809 | 61.42975 | 60.72221 | 67.39605 | 73.02903 | 42.99855 | 62.26323 | 64.30298 | 53.00817 | 35.80357 | 43.33262 | 46.43126 | 29.68961 | 48.03786 | 47.92376 | 54.2866 | 61.23975 | 52.5191 | 55.84417 | 55.20985 |
| **ATG12** | 35.00331 | 31.76086 | 22.87892 | 27.59324 | 25.20924 | 19.5863 | 26.75894 | 29.40919 | 28.5161 | 24.46469 | 40.94021 | 38.2562 | 14.68901 | 17.30506 | 18.12091 | 9.390592 | 10.2058 | 12.14144 | 11.18221 | 9.952543 | 10.0393 | 16.47658 | 17.149 | 14.40257 |
| **ATG16L1** | 13.78918 | 14.34361 | 1.759917 | 16.79589 | 16.80616 | 17.80572 | 24.70056 | 15.92998 | 16.69235 | 34.84365 | 15.35258 | 18.72112 | 17.24362 | 12.53125 | 10.24226 | 8.347192 | 10.2058 | 14.78088 | 14.77649 | 12.66687 | 14.55699 | 9.268076 | 17.149 | 14.40257 |
| **ATG5** | 67.88522 | 46.10447 | 45.75783 | 70.78266 | 64.07348 | 60.53946 | 69.98493 | 72.29758 | 77.20212 | 69.68731 | 75.05704 | 79.76825 | 54.92413 | 45.94791 | 44.12049 | 52.16995 | 38.96761 | 58.06774 | 53.1155 | 54.2866 | 41.66311 | 50.97442 | 54.96473 | 60.81085 |
| **ATG7** | 19.09272 | 15.36816 | 15.83925 | 25.19383 | 18.90693 | 21.36687 | 38.08003 | 12.25383 | 20.86544 | 27.43011 | 22.17594 | 21.97697 | 35.1259 | 35.20684 | 48.84768 | 52.69165 | 29.68961 | 60.17929 | 65.4958 | 36.19107 | 54.21224 | 55.09356 | 56.72361 | 54.00964 |
| **ATM** | 10.60707 | 11.26998 | 10.5595 | 7.198237 | 6.30231 | 1.780572 | 10.2919 | 6.126914 | 6.259631 | 8.154897 | 8.529209 | 4.883771 | 3.831916 | 9.547618 | 5.515061 | 3.651897 | 7.422402 | 2.639443 | 5.19174 | 2.261942 | 1.00393 | 3.089359 | 4.397179 | 5.601 |
| **B2M** | 17017.98 | 19973.48 | 12678.44 | 20723.72 | 12760.08 | 20621.7 | 17075.29 | 18932.16 | 17553.4 | 14299.24 | 16306.14 | 16237.72 | 12632.55 | 14131.67 | 8702.766 | 13047.71 | 7080.971 | 15450.24 | 12862.34 | 12170.6 | 13142.95 | 9730.45 | 11430.47 | 12373.81 |
| **B3GAT1** | 2.121413 | 8.19635 | 5.27975 | 2.399412 | 4.20154 | 3.561145 | 3.08757 | 2.450765 | 6.259631 | 2.224063 | 3.411684 | 1.627924 | 3.193263 | 1.790178 | 3.151463 | 4.173596 | 3.711201 | 0.527889 | 3.194917 | 4.071495 | 4.015721 | 3.089359 | 4.397179 | 2.000357 |
| **BATF** | 1.060707 | 6.147263 | 1.759917 | 1.199706 | 3.151155 | 6.232003 | 3.08757 | 4.901531 | 4.173088 | 1.482709 | 4.264605 | 1.627924 | 11.49575 | 9.547618 | 7.878658 | 13.04249 | 12.0614 | 8.446217 | 9.185387 | 9.047766 | 12.04716 | 15.44679 | 12.3121 | 17.60314 |
| **BATF3** | 1.060707 | 6.147263 | 5.27975 | 5.998531 | 8.403079 | 3.561145 | 11.32109 | 7.352296 | 4.173088 | 5.18948 | 0.852921 | 5.697732 | 90.05002 | 59.67261 | 70.12006 | 42.25766 | 28.76181 | 70.20918 | 100.2405 | 61.52481 | 73.78888 | 83.41268 | 124.0004 | 62.41114 |
| **BAX** | 246.0839 | 218.2278 | 181.2714 | 232.743 | 183.8174 | 190.5212 | 255.2391 | 257.3304 | 167.619 | 219.4409 | 195.3189 | 212.444 | 171.1589 | 166.4866 | 121.3313 | 167.4655 | 112.2638 | 161.5339 | 161.3433 | 166.0265 | 145.5699 | 151.8935 | 174.1283 | 185.6331 |
| **BCAP31** | 220.627 | 191.5897 | 163.6723 | 293.928 | 186.9685 | 259.0733 | 291.2608 | 287.9649 | 217.0006 | 192.7521 | 263.5526 | 258.0259 | 129.6465 | 132.4732 | 90.60457 | 124.6862 | 89.99662 | 108.745 | 134.1865 | 143.4071 | 105.9147 | 114.3063 | 138.0714 | 128.4229 |
| **BCL10** | 141.074 | 184.4179 | 110.8748 | 190.7533 | 102.9377 | 125.5303 | 183.1958 | 191.1597 | 170.4011 | 149.0122 | 192.7601 | 166.0482 | 132.2011 | 194.5327 | 116.6041 | 201.8977 | 121.5418 | 110.8566 | 147.7649 | 161.955 | 146.0719 | 133.8722 | 165.3339 | 174.8312 |
| **BCL2** | 8.485652 | 2.049088 | 8.799583 | 7.198237 | 5.251925 | 8.902862 | 7.204331 | 8.577679 | 6.955146 | 8.154897 | 5.117526 | 11.39546 | 23.63015 | 14.91815 | 24.42384 | 26.60668 | 14.8448 | 14.78088 | 19.1695 | 19.4527 | 21.08254 | 24.19998 | 23.30505 | 26.80478 |
| **BCL2L11** | 13.78918 | 11.26998 | 7.039667 | 33.59177 | 14.70539 | 22.25715 | 8.233521 | 22.05689 | 9.737204 | 11.12031 | 11.08797 | 16.27924 | 10.21844 | 10.14434 | 7.878658 | 13.04249 | 12.0614 | 10.55777 | 9.584751 | 14.02404 | 6.023582 | 7.208504 | 13.19154 | 9.201642 |
| **BCL3** | 37.12473 | 79.91442 | 35.19833 | 85.17914 | 75.62772 | 37.39202 | 45.28436 | 100.4814 | 31.29816 | 31.13688 | 36.6756 | 34.18639 | 31.29398 | 134.8601 | 80.36231 | 95.99271 | 86.28542 | 54.90041 | 41.53392 | 96.35871 | 34.6356 | 26.77444 | 48.80868 | 31.60564 |
| **BCL6** | 15.9106 | 15.36816 | 5.27975 | 19.1953 | 22.05808 | 14.24458 | 16.46704 | 75.97373 | 13.91029 | 9.637606 | 12.79381 | 13.02339 | 150.722 | 153.9553 | 148.9066 | 150.7712 | 94.63562 | 102.4104 | 114.2183 | 177.3362 | 83.82818 | 97.82969 | 141.5892 | 139.6249 |
| **BID** | 2.121413 | 7.171807 | 3.519833 | 3.599119 | 8.403079 | 8.012575 | 12.35028 | 7.352296 | 9.737204 | 7.413543 | 5.970447 | 7.325656 | 7.025179 | 5.370535 | 7.090792 | 12.52079 | 12.0614 | 7.918328 | 8.386658 | 9.952543 | 11.04323 | 10.29786 | 6.595768 | 8.4015 |
| **BLNK** | 196.2307 | 154.7061 | 175.9917 | 309.5242 | 148.1043 | 245.719 | 327.2824 | 204.6389 | 280.2924 | 417.3825 | 281.4639 | 341.8639 | 21.71419 | 19.09524 | 33.09036 | 13.04249 | 12.9892 | 24.28287 | 22.76378 | 22.16703 | 20.58057 | 29.8638 | 24.18448 | 30.00536 |
| **BST1** | 37.12473 | 39.95721 | 17.59917 | 47.98825 | 31.51155 | 62.32003 | 57.63464 | 35.5361 | 36.16676 | 40.77449 | 41.79313 | 36.62828 | 40.87377 | 41.1741 | 40.18116 | 40.69256 | 37.11201 | 64.93029 | 50.31995 | 53.83421 | 61.74172 | 49.94463 | 67.27684 | 63.61135 |
| **BST2** | 57.27815 | 91.1844 | 52.7975 | 71.98237 | 42.0154 | 57.8686 | 54.54707 | 51.46607 | 54.25014 | 54.86022 | 49.46941 | 105.0011 | 47.2603 | 66.83333 | 29.9389 | 49.03976 | 35.25641 | 64.93029 | 46.72566 | 41.61973 | 35.63953 | 40.67656 | 45.73066 | 48.80871 |
| **BTK** | 114.5563 | 93.23349 | 91.51567 | 148.7636 | 112.3912 | 125.5303 | 188.3418 | 131.116 | 150.9267 | 165.322 | 153.5258 | 157.0946 | 134.1171 | 95.47618 | 96.9075 | 118.4258 | 82.57422 | 123.5259 | 142.5732 | 127.5735 | 123.9854 | 135.9318 | 135.8728 | 164.4293 |
| **BTLA** | 222.7484 | 138.3134 | 158.3925 | 219.5462 | 167.0112 | 262.6344 | 406.5301 | 172.779 | 379.751 | 297.2831 | 336.9038 | 347.5617 | 24.90745 | 4.177083 | 9.45439 | 11.99909 | 12.9892 | 14.78088 | 28.75425 | 15.3812 | 39.15328 | 19.05105 | 36.05687 | 45.20807 |
| **C14orf166** | 612.0277 | 610.6281 | 499.8163 | 742.6181 | 505.2352 | 642.7866 | 821.2937 | 618.8183 | 635.7003 | 673.1497 | 723.277 | 692.6815 | 580.5352 | 564.5029 | 486.9011 | 565.5223 | 366.4811 | 628.1874 | 662.1466 | 563.2235 | 606.8759 | 568.9569 | 651.6619 | 672.52 |
| **C1QA** | 10.60707 | 12.29453 | 15.83925 | 3.599119 | 12.60462 | 9.793148 | 4.11676 | 4.901531 | 11.12823 | 8.896252 | 12.79381 | 3.255847 | 8.302484 | 10.14434 | 8.666524 | 7.825493 | 8.350202 | 20.05976 | 7.188564 | 5.881048 | 8.533408 | 8.753183 | 6.595768 | 3.200571 |
| **C1QB** | 5.303533 | 5.122719 | 8.799583 | 2.399412 | 4.20154 | 2.670858 | 5.14595 | 6.126914 | 2.782058 | 3.706772 | 1.705842 | 6.511694 | 8.941137 | 16.70833 | 7.878658 | 6.260394 | 24.12281 | 49.09364 | 6.789199 | 9.952543 | 4.015721 | 4.119145 | 4.836897 | 6.801214 |
| **C1QBP** | 353.2153 | 291.995 | 263.9875 | 347.9148 | 245.7901 | 313.3807 | 341.6911 | 377.4179 | 268.4686 | 308.4034 | 342.0213 | 311.7474 | 319.965 | 271.5104 | 249.7535 | 301.0206 | 192.0547 | 290.8666 | 352.2396 | 359.6487 | 303.6889 | 299.6678 | 319.6749 | 348.8623 |
| **C1R** | 2.121413 | 5.122719 | 3.519833 | 1.199706 | 6.30231 | 0.890286 | 3.08757 | 1.225383 | 2.086544 | 3.706772 | 0.852921 | 1.627924 | 7.663832 | 3.580357 | 0.787866 | 5.216995 | 3.711201 | 3.69522 | 1.198094 | 2.71433 | 2.007861 | 2.574466 | 3.078025 | 3.200571 |
| **C1S** | 2.121413 | 5.122719 | 7.039667 | 10.79736 | 4.20154 | 2.670858 | 4.11676 | 8.577679 | 9.04169 | 6.672189 | 4.264605 | 5.697732 | 6.386526 | 7.160714 | 10.24226 | 5.216995 | 3.711201 | 8.974105 | 2.396188 | 6.333436 | 4.517687 | 3.089359 | 4.397179 | 4.000714 |
| **C2** | 2.121413 | 4.098175 | 15.83925 | 1.199706 | 2.10077 | 8.012575 | 2.05838 | 8.577679 | 9.737204 | 2.965417 | 5.970447 | 0.813962 | 3.193263 | 6.563988 | 7.090792 | 2.086798 | 5.566801 | 7.39044 | 3.594282 | 4.071495 | 4.015721 | 2.574466 | 2.638307 | 4.400786 |
| **C3** | 6.364239 | 9.220894 | 3.519833 | 5.998531 | 5.251925 | 1.780572 | 1.02919 | 7.352296 | 3.477573 | 3.706772 | 1.705842 | 2.441885 | 3.831916 | 4.177083 | 6.302927 | 5.216995 | 7.422402 | 4.750997 | 1.198094 | 4.071495 | 4.517687 | 2.059572 | 3.078025 | 3.600643 |
| **C4A/B** | 8.485652 | 4.098175 | 14.07933 | 1.199706 | 11.55423 | 5.341717 | 2.05838 | 4.901531 | 14.60581 | 2.965417 | 3.411684 | 4.883771 | 2.554611 | 3.580357 | 5.515061 | 3.651897 | 13.917 | 4.223108 | 5.591105 | 5.42866 | 8.031443 | 3.089359 | 1.758872 | 2.8005 |
| **C4BPA** | 30.76049 | 44.05538 | 28.15867 | 40.79001 | 33.61232 | 34.72116 | 34.99246 | 34.31072 | 50.77257 | 25.9474 | 26.44055 | 34.18639 | 34.48724 | 36.40029 | 34.6661 | 35.99727 | 18.556 | 38.00798 | 36.74155 | 40.71495 | 44.17294 | 33.98295 | 38.25546 | 38.40686 |
| **C5** | 8.485652 | 7.171807 | 14.07933 | 2.399412 | 12.60462 | 8.902862 | 12.35028 | 4.901531 | 8.346175 | 7.413543 | 7.676288 | 5.697732 | 15.32766 | 12.53125 | 8.666524 | 11.99909 | 6.494602 | 7.918328 | 11.18221 | 9.500155 | 9.537338 | 10.29786 | 12.3121 | 12.80229 |
| **C6** | 14.84989 | 11.26998 | 12.31942 | 8.397943 | 13.655 | 11.57372 | 8.233521 | 12.25383 | 7.650661 | 9.637606 | 9.38213 | 4.883771 | 7.025179 | 10.74107 | 6.302927 | 6.782094 | 13.917 | 11.61355 | 8.386658 | 9.047766 | 5.019652 | 6.178717 | 7.475204 | 8.4015 |
| **C7** | 4.242826 | 1.024544 | 5.27975 | 1.199706 | 7.352695 | 5.341717 | 3.08757 | 3.676148 | 2.782058 | 5.930835 | 4.264605 | 2.441885 | 3.193263 | 4.773809 | 2.363597 | 4.173596 | 3.711201 | 1.583666 | 2.396188 | 2.71433 | 3.011791 | 3.089359 | 0.879436 | 2.8005 |
| **C8A** | 2.121413 | 6.147263 | 1.759917 | 2.399412 | 7.352695 | 3.561145 | 2.05838 | 4.901531 | 3.477573 | 3.706772 | 3.411684 | 0.813962 | 2.554611 | 2.386905 | 4.727195 | 3.130197 | 2.783401 | 1.583666 | 1.597459 | 1.809553 | 2.509826 | 2.574466 | 3.078025 | 0.800143 |
| **C8B** | 2.121413 | 2.049088 | 5.27975 | 2.399412 | 5.251925 | 4.451431 | 1.02919 | 3.676148 | 2.086544 | 1.482709 | 1.705842 | 1.627924 | 4.470568 | 1.790178 | 1.575732 | 3.130197 | 3.711201 | 2.639443 | 2.396188 | 2.71433 | 2.007861 | 3.089359 | 2.198589 | 3.200571 |
| **C8G** | 1.060707 | 2.049088 | 1.759917 | 4.798825 | 1.050385 | 1.780572 | 1.02919 | 1.225383 | 2.086544 | 8.154897 | 5.117526 | 2.441885 | 1.915958 | 1.790178 | 3.939329 | 1.043399 | 3.711201 | 2.639443 | 1.198094 | 2.71433 | 2.509826 | 4.119145 | 2.198589 | 2.000357 |
| **C9** | 1.060707 | 4.098175 | 3.519833 | 2.399412 | 1.050385 | 1.780572 | 1.02919 | 2.450765 | 0.695515 | 4.448126 | 2.558763 | 3.255847 | 1.277305 | 1.193452 | 2.363597 | 1.565099 | 3.711201 | 2.111554 | 1.996823 | 3.166718 | 2.509826 | 2.059572 | 0.439718 | 1.200214 |
| **CAMP** | 4.242826 | 2.049088 | 3.519833 | 3.599119 | 1.050385 | 3.561145 | 3.08757 | 3.676148 | 2.782058 | 2.965417 | 5.117526 | 4.883771 | 1.915958 | 5.370535 | 7.090792 | 5.216995 | 3.711201 | 0.527889 | 1.996823 | 3.619107 | 2.509826 | 4.119145 | 2.638307 | 2.8005 |
| **CARD9** | 2.121413 | 5.122719 | 3.519833 | 4.798825 | 4.20154 | 4.451431 | 1.02919 | 3.676148 | 3.477573 | 1.482709 | 4.264605 | 0.813962 | 17.88227 | 13.12798 | 20.48451 | 14.60759 | 23.19501 | 22.17132 | 20.3676 | 13.57165 | 17.56878 | 23.17019 | 24.18448 | 22.80407 |
| **CASP1** | 8.485652 | 8.19635 | 17.59917 | 3.599119 | 10.50385 | 27.59887 | 11.32109 | 4.901531 | 7.650661 | 14.08573 | 8.529209 | 11.39546 | 561.3757 | 606.2738 | 491.6283 | 554.0449 | 358.1309 | 720.5679 | 575.4845 | 611.629 | 526.5615 | 558.1441 | 495.562 | 620.9108 |
| **CASP10** | 3.18212 | 3.073631 | 7.039667 | 2.399412 | 2.10077 | 5.341717 | 4.11676 | 2.450765 | 4.173088 | 4.448126 | 1.705842 | 5.697732 | 3.831916 | 2.983631 | 5.515061 | 5.738695 | 3.711201 | 2.639443 | 5.591105 | 2.71433 | 6.023582 | 3.604252 | 3.078025 | 6.001071 |
| **CASP2** | 45.61038 | 14.34361 | 24.63883 | 41.98972 | 32.56193 | 37.39202 | 58.66383 | 41.66301 | 43.81742 | 45.96397 | 57.99862 | 58.60525 | 31.93263 | 21.48214 | 25.99957 | 36.51897 | 29.68961 | 22.69921 | 45.92693 | 26.23852 | 36.14149 | 43.25102 | 48.36897 | 44.40793 |
| **CASP3** | 26.51766 | 48.15356 | 17.59917 | 29.99265 | 36.76347 | 37.39202 | 58.66383 | 23.28227 | 57.0322 | 59.30835 | 54.58694 | 49.65167 | 15.96632 | 23.86905 | 18.90878 | 16.69438 | 21.33941 | 18.4761 | 38.33901 | 14.47643 | 36.64346 | 37.5872 | 47.48953 | 42.80764 |
| **CASP8** | 85.91723 | 73.76715 | 54.55742 | 73.18208 | 57.77117 | 84.57719 | 138.9407 | 60.04375 | 88.33035 | 106.755 | 93.8213 | 110.6988 | 107.2936 | 66.2366 | 148.1188 | 121.0343 | 69.58502 | 90.26894 | 180.5128 | 84.14423 | 150.5896 | 170.9445 | 173.2488 | 197.2352 |
| **CCBP2** | 6.364239 | 1.024544 | 3.519833 | 4.798825 | 3.151155 | 3.561145 | 3.08757 | 3.676148 | 1.391029 | 2.965417 | 4.264605 | 4.069809 | 4.470568 | 1.790178 | 5.515061 | 4.173596 | 2.783401 | 4.750997 | 5.591105 | 3.166718 | 5.019652 | 3.604252 | 5.276615 | 4.800857 |
| **CCL11** | 7.424946 | 2.049088 | 7.039667 | 2.399412 | 1.050385 | 4.451431 | 5.14595 | 1.225383 | 10.43272 | 3.706772 | 6.823368 | 4.069809 | 4.470568 | 4.177083 | 3.151463 | 1.565099 | 10.2058 | 2.639443 | 4.792376 | 4.976272 | 5.019652 | 3.089359 | 3.078025 | 3.600643 |
| **CCL13** | 7.424946 | 2.049088 | 1.759917 | 1.199706 | 2.10077 | 3.561145 | 2.05838 | 8.577679 | 7.650661 | 5.930835 | 5.117526 | 2.441885 | 1.915958 | 3.580357 | 8.666524 | 4.695296 | 12.0614 | 3.69522 | 5.19174 | 5.42866 | 5.019652 | 1.029786 | 3.517743 | 3.200571 |
| **CCL15** | 9.546359 | 6.147263 | 8.799583 | 3.599119 | 9.453464 | 8.012575 | 3.08757 | 1.225383 | 4.173088 | 4.448126 | 4.264605 | 7.325656 | 4.470568 | 8.354166 | 7.090792 | 7.303793 | 11.1336 | 3.167331 | 4.393011 | 3.166718 | 3.011791 | 4.634038 | 4.397179 | 2.000357 |
| **CCL16** | 4.242826 | 6.147263 | 5.27975 | 3.599119 | 2.10077 | 3.561145 | 1.02919 | 3.676148 | 4.868602 | 6.672189 | 2.558763 | 4.069809 | 3.831916 | 1.193452 | 8.666524 | 2.086798 | 4.639001 | 2.639443 | 3.194917 | 2.261942 | 3.011791 | 2.059572 | 3.078025 | 4.800857 |
| **CCL18** | 12.72848 | 2.049088 | 12.31942 | 1.199706 | 1.050385 | 4.451431 | 1.02919 | 1.225383 | 2.782058 | 2.965417 | 3.411684 | 4.069809 | 4.470568 | 5.370535 | 3.151463 | 3.651897 | 5.566801 | 3.167331 | 3.194917 | 2.71433 | 4.015721 | 3.604252 | 2.638307 | 2.400428 |
| **CCL19** | 6.364239 | 7.171807 | 8.799583 | 1.199706 | 5.251925 | 3.561145 | 5.14595 | 2.450765 | 4.173088 | 7.413543 | 7.676288 | 5.697732 | 8.302484 | 8.354166 | 3.151463 | 3.651897 | 8.350202 | 5.278885 | 2.396188 | 4.071495 | 3.513756 | 4.119145 | 2.638307 | 3.600643 |
| **CCL2** | 1.060707 | 2.049088 | 1.759917 | 1.199706 | 3.151155 | 1.780572 | 4.11676 | 1.225383 | 3.477573 | 1.482709 | 0.852921 | 1.627924 | 0.638653 | 2.983631 | 0.787866 | 2.086798 | 1.8556 | 2.111554 | 0.798729 | 2.71433 | 2.509826 | 1.544679 | 0.879436 | 4.800857 |
| **CCL20** | 3.18212 | 4.098175 | 5.27975 | 2.399412 | 4.20154 | 0.890286 | 2.05838 | 2.450765 | 4.173088 | 4.448126 | 5.970447 | 4.069809 | 0.638653 | 5.370535 | 3.151463 | 3.130197 | 4.639001 | 4.223108 | 4.393011 | 0.904777 | 0.501965 | 4.634038 | 2.638307 | 2.400428 |
| **CCL22** | 7.424946 | 6.147263 | 3.519833 | 5.998531 | 7.352695 | 4.451431 | 5.14595 | 4.901531 | 4.173088 | 8.154897 | 7.676288 | 2.441885 | 4.470568 | 2.983631 | 3.939329 | 4.173596 | 8.350202 | 3.69522 | 4.393011 | 4.071495 | 4.517687 | 9.268076 | 5.276615 | 6.001071 |
| **CCL23** | 4.242826 | 1.024544 | 5.27975 | 5.998531 | 1.050385 | 2.670858 | 1.02919 | 3.676148 | 4.173088 | 2.224063 | 3.411684 | 2.441885 | 3.193263 | 1.193452 | 3.151463 | 2.608498 | 4.639001 | 3.69522 | 4.792376 | 3.166718 | 2.509826 | 2.059572 | 2.638307 | 2.400428 |
| **CCL24** | 6.364239 | 6.147263 | 5.27975 | 7.198237 | 17.85654 | 4.451431 | 4.11676 | 8.577679 | 6.955146 | 4.448126 | 4.264605 | 5.697732 | 4.470568 | 5.370535 | 9.45439 | 4.173596 | 10.2058 | 4.750997 | 3.194917 | 6.333436 | 7.027513 | 5.663824 | 4.397179 | 3.200571 |
| **CCL26** | 3.18212 | 5.122719 | 8.799583 | 1.199706 | 2.10077 | 2.670858 | 2.05838 | 3.676148 | 1.391029 | 4.448126 | 4.264605 | 3.255847 | 1.915958 | 2.386905 | 3.151463 | 1.565099 | 2.783401 | 3.167331 | 2.396188 | 4.071495 | 3.513756 | 3.604252 | 4.397179 | 4.000714 |
| **CCL3** | 5.303533 | 8.19635 | 12.31942 | 5.998531 | 4.20154 | 3.561145 | 2.05838 | 4.901531 | 1.391029 | 4.448126 | 5.970447 | 4.069809 | 11.49575 | 12.53125 | 11.81799 | 7.303793 | 17.6282 | 6.334663 | 23.56251 | 16.28598 | 12.04716 | 41.19145 | 27.70223 | 16.803 |
| **CCL4** | 5.303533 | 7.171807 | 10.5595 | 9.597649 | 5.251925 | 6.232003 | 7.204331 | 4.901531 | 6.259631 | 6.672189 | 5.970447 | 6.511694 | 7.663832 | 8.950892 | 11.81799 | 9.390592 | 9.278002 | 4.750997 | 7.188564 | 7.690601 | 9.537338 | 16.47658 | 12.75182 | 9.601714 |
| **CCL5** | 10.60707 | 9.220894 | 12.31942 | 11.99706 | 9.453464 | 9.793148 | 12.35028 | 9.803062 | 6.259631 | 5.930835 | 8.529209 | 6.511694 | 8.302484 | 16.70833 | 11.03012 | 15.65099 | 12.9892 | 17.42032 | 14.77649 | 19.90509 | 18.07075 | 32.43827 | 15.39013 | 16.40293 |
| **CCL7** | 2.121413 | 1.024544 | 3.519833 | 1.199706 | 1.050385 | 0.890286 | 3.08757 | 1.225383 | 4.868602 | 0.741354 | 2.558763 | 2.441885 | 1.277305 | 2.386905 | 3.151463 | 2.086798 | 1.8556 | 1.055777 | 1.996823 | 2.261942 | 0.501965 | 0.514893 | 0.439718 | 1.200214 |
| **CCL8** | 2.121413 | 5.122719 | 1.759917 | 5.998531 | 8.403079 | 0.890286 | 6.175141 | 1.225383 | 4.173088 | 5.930835 | 4.264605 | 4.883771 | 7.025179 | 4.773809 | 7.878658 | 3.651897 | 2.783401 | 3.69522 | 2.795553 | 6.785825 | 5.521617 | 3.604252 | 3.517743 | 3.600643 |
| **CCND3** | 183.5022 | 150.6079 | 119.6743 | 152.3627 | 155.457 | 195.863 | 182.1666 | 235.2735 | 206.5678 | 126.7716 | 161.2021 | 178.2576 | 159.6632 | 170.6637 | 133.9372 | 155.9882 | 181.8488 | 157.8387 | 158.9471 | 175.9791 | 200.7861 | 204.4126 | 193.9156 | 190.8341 |
| **CCR1** | 39.24614 | 46.10447 | 70.39667 | 31.19236 | 52.51925 | 89.02862 | 143.0574 | 49.01531 | 57.0322 | 75.61814 | 93.8213 | 127.792 | 31.93263 | 189.1622 | 112.6648 | 161.7269 | 216.1775 | 78.12751 | 122.2056 | 172.36 | 77.30264 | 71.57014 | 100.6954 | 137.2245 |
| **CCR10** | 5.303533 | 4.098175 | 5.27975 | 1.199706 | 3.151155 | 3.561145 | 4.11676 | 3.676148 | 2.782058 | 0.741354 | 1.705842 | 0.813962 | 5.109221 | 4.773809 | 7.878658 | 3.130197 | 3.711201 | 3.69522 | 1.597459 | 2.71433 | 1.505896 | 2.059572 | 3.078025 | 4.400786 |
| **CCR2** | 277.9051 | 167.0006 | 211.19 | 358.7121 | 277.3016 | 427.3374 | 681.3238 | 219.3435 | 452.0845 | 462.6051 | 585.1038 | 660.123 | 75.99966 | 50.125 | 74.05939 | 117.9041 | 77.00742 | 161.5339 | 222.8455 | 46.14361 | 197.2723 | 193.0849 | 161.8162 | 282.4504 |
| **CCR5** | 117.7384 | 59.42354 | 45.75783 | 91.17767 | 73.52695 | 112.1761 | 161.5828 | 60.04375 | 181.5293 | 201.6484 | 165.4667 | 202.6765 | 48.5376 | 81.75148 | 55.93847 | 79.29833 | 77.00742 | 68.09762 | 125.4005 | 68.31064 | 112.9422 | 100.4042 | 80.46837 | 124.4222 |
| **CCR6** | 12.72848 | 16.3927 | 10.5595 | 23.99412 | 8.403079 | 9.793148 | 8.233521 | 12.25383 | 19.47441 | 12.60302 | 26.44055 | 13.83735 | 35.76455 | 47.14136 | 39.39329 | 85.55872 | 45.46221 | 30.61754 | 27.55616 | 52.92943 | 76.80067 | 64.87653 | 116.0855 | 120.0214 |
| **CCR7** | 6.364239 | 3.073631 | 7.039667 | 4.798825 | 8.403079 | 8.902862 | 13.37947 | 4.901531 | 10.43272 | 11.86167 | 11.08797 | 15.46527 | 1.915958 | 1.790178 | 4.727195 | 0.5217 | 7.422402 | 2.111554 | 4.393011 | 2.71433 | 3.513756 | 3.604252 | 1.319154 | 2.000357 |
| **CCR8** | 4.242826 | 4.098175 | 1.759917 | 4.798825 | 7.352695 | 3.561145 | 3.08757 | 1.225383 | 3.477573 | 2.224063 | 3.411684 | 1.627924 | 1.277305 | 3.580357 | 8.666524 | 1.043399 | 3.711201 | 2.639443 | 0.399365 | 2.71433 | 2.007861 | 1.029786 | 0.439718 | 0.800143 |
| **CCR9** | 7.424946 | 11.26998 | 8.799583 | 3.599119 | 4.20154 | 4.451431 | 4.11676 | 3.676148 | 3.477573 | 2.965417 | 5.970447 | 4.069809 | 2.554611 | 4.773809 | 3.151463 | 4.173596 | 4.639001 | 2.639443 | 2.795553 | 2.71433 | 3.011791 | 4.119145 | 3.078025 | 3.200571 |
| **CCRL1** | 3.18212 | 4.098175 | 3.519833 | 3.599119 | 7.352695 | 4.451431 | 2.05838 | 4.901531 | 3.477573 | 1.482709 | 9.38213 | 4.883771 | 5.109221 | 2.386905 | 4.727195 | 4.173596 | 1.8556 | 4.750997 | 4.393011 | 4.976272 | 2.007861 | 2.574466 | 3.957461 | 3.200571 |
| **CCRL2** | 8.485652 | 12.29453 | 1.759917 | 4.798825 | 8.403079 | 9.793148 | 10.2919 | 11.02844 | 11.82375 | 6.672189 | 9.38213 | 5.697732 | 7.025179 | 12.53125 | 5.515061 | 14.60759 | 9.278002 | 5.806774 | 11.98094 | 12.21448 | 15.05896 | 12.35743 | 10.11351 | 13.60243 |
| **CD14** | 4.242826 | 6.147263 | 3.519833 | 1.199706 | 4.20154 | 6.232003 | 3.08757 | 2.450765 | 1.391029 | 8.896252 | 4.264605 | 2.441885 | 19.79823 | 39.98065 | 55.93847 | 58.43035 | 39.89541 | 25.86654 | 40.33583 | 39.35778 | 39.15328 | 45.31059 | 43.53207 | 59.61064 |
| **CD160** | 4.242826 | 1.024544 | 10.5595 | 3.599119 | 2.10077 | 5.341717 | 3.08757 | 12.25383 | 5.564117 | 3.706772 | 3.411684 | 7.325656 | 2.554611 | 4.177083 | 3.151463 | 2.608498 | 8.350202 | 4.223108 | 1.597459 | 3.166718 | 6.023582 | 3.089359 | 3.957461 | 4.800857 |
| **CD163** | 6.364239 | 5.122719 | 15.83925 | 8.397943 | 6.30231 | 8.902862 | 8.233521 | 7.352296 | 10.43272 | 2.224063 | 6.823368 | 6.511694 | 116.2348 | 156.3422 | 128.4221 | 159.1184 | 96.49123 | 109.8008 | 148.1643 | 147.0262 | 134.5267 | 185.3615 | 174.1283 | 163.6292 |
| **CD164** | 1095.71 | 817.5859 | 674.0481 | 1163.715 | 790.9399 | 909.8725 | 1134.167 | 996.2362 | 1298.526 | 1507.173 | 1375.761 | 1596.179 | 263.1249 | 221.3854 | 169.3912 | 253.546 | 173.4986 | 240.7172 | 310.3063 | 278.6712 | 326.2774 | 315.6295 | 353.9729 | 366.8655 |
| **CD19** | 7.424946 | 3.073631 | 10.5595 | 2.399412 | 5.251925 | 7.122289 | 3.08757 | 3.676148 | 6.259631 | 8.896252 | 7.676288 | 5.697732 | 5.109221 | 8.354166 | 6.302927 | 5.216995 | 13.917 | 7.918328 | 6.789199 | 7.238213 | 6.023582 | 7.208504 | 5.276615 | 3.600643 |
| **CD1A** | 7.424946 | 5.122719 | 12.31942 | 1.199706 | 3.151155 | 8.902862 | 4.11676 | 4.901531 | 2.782058 | 1.482709 | 1.705842 | 0.813962 | 28.73937 | 19.69196 | 28.36317 | 27.12838 | 25.97841 | 17.42032 | 18.37077 | 14.47643 | 27.10612 | 20.08083 | 25.06392 | 40.40721 |
| **CD1D** | 6.364239 | 2.049088 | 8.799583 | 5.998531 | 5.251925 | 6.232003 | 3.08757 | 3.676148 | 6.955146 | 2.224063 | 3.411684 | 4.883771 | 293.1416 | 235.7068 | 228.4811 | 273.3706 | 157.726 | 269.751 | 462.0649 | 188.6459 | 290.6378 | 294.5189 | 401.9021 | 440.4786 |
| **CD2** | 5.303533 | 4.098175 | 1.759917 | 5.998531 | 6.30231 | 9.793148 | 9.262711 | 2.450765 | 15.30132 | 2.965417 | 7.676288 | 12.20943 | 31.93263 | 15.51488 | 16.54518 | 35.99727 | 30.61741 | 29.56176 | 31.54981 | 32.57196 | 40.15721 | 44.7957 | 43.53207 | 34.00607 |
| **CD209** | 3.18212 | 5.122719 | 3.519833 | 7.198237 | 8.403079 | 8.012575 | 2.05838 | 1.225383 | 4.173088 | 6.672189 | 4.264605 | 5.697732 | 7.025179 | 7.75744 | 7.878658 | 10.43399 | 3.711201 | 6.862551 | 11.18221 | 7.690601 | 6.023582 | 5.148931 | 7.035486 | 10.80193 |
| **CD22** | 19.09272 | 23.56451 | 10.5595 | 13.19677 | 29.41078 | 32.0503 | 31.90489 | 22.05689 | 20.86544 | 13.34438 | 13.64674 | 24.41885 | 33.84859 | 28.64285 | 23.63597 | 29.73687 | 34.32861 | 47.50997 | 33.94599 | 29.85763 | 56.72207 | 44.7957 | 36.05687 | 43.20771 |
| **CD24** | 1.060707 | 5.122719 | 3.519833 | 1.199706 | 8.403079 | 7.122289 | 4.11676 | 2.450765 | 1.391029 | 4.448126 | 3.411684 | 1.627924 | 0.638653 | 4.773809 | 5.515061 | 1.043399 | 3.711201 | 2.111554 | 2.795553 | 2.71433 | 3.011791 | 2.059572 | 2.638307 | 2.000357 |
| **CD244** | 11.66777 | 5.122719 | 3.519833 | 4.798825 | 2.10077 | 8.902862 | 13.37947 | 7.352296 | 13.21478 | 5.930835 | 8.529209 | 12.20943 | 85.57945 | 114.5714 | 118.1799 | 105.3833 | 65.87382 | 99.24305 | 122.2056 | 136.1689 | 100.895 | 115.3361 | 140.27 | 142.4254 |
| **CD247** | 18.03201 | 11.26998 | 17.59917 | 5.998531 | 4.20154 | 8.902862 | 6.175141 | 6.126914 | 5.564117 | 8.154897 | 5.117526 | 2.441885 | 5.109221 | 1.790178 | 0.787866 | 4.173596 | 8.350202 | 4.223108 | 4.792376 | 9.047766 | 7.027513 | 4.119145 | 4.397179 | 7.601357 |
| **CD27** | 5.303533 | 1.024544 | 8.799583 | 2.399412 | 3.151155 | 5.341717 | 3.08757 | 4.901531 | 2.782058 | 0.741354 | 1.705842 | 3.255847 | 3.831916 | 1.193452 | 3.939329 | 1.565099 | 0.9278 | 3.69522 | 3.594282 | 2.261942 | 1.505896 | 0.514893 | 2.638307 | 4.800857 |
| **CD274** | 3.18212 | 3.073631 | 1.759917 | 1.199706 | 1.050385 | 0.890286 | 2.05838 | 1.225383 | 3.477573 | 0.741354 | 3.411684 | 1.627924 | 4.470568 | 2.983631 | 1.575732 | 2.608498 | 5.566801 | 2.111554 | 1.597459 | 3.619107 | 2.007861 | 4.119145 | 1.319154 | 2.000357 |
| **CD276** | 7.424946 | 6.147263 | 12.31942 | 4.798825 | 6.30231 | 4.451431 | 1.02919 | 6.126914 | 8.346175 | 4.448126 | 9.38213 | 4.883771 | 2.554611 | 4.177083 | 5.515061 | 2.086798 | 8.350202 | 5.806774 | 4.393011 | 4.071495 | 5.521617 | 6.178717 | 5.276615 | 3.600643 |
| **CD28** | 7.424946 | 5.122719 | 7.039667 | 1.199706 | 10.50385 | 6.232003 | 5.14595 | 12.25383 | 7.650661 | 5.18948 | 6.823368 | 9.767541 | 5.109221 | 5.370535 | 7.090792 | 6.260394 | 10.2058 | 4.223108 | 5.19174 | 8.595378 | 7.027513 | 2.574466 | 7.035486 | 2.8005 |
| **CD34** | 1.060707 | 2.049088 | 1.759917 | 1.199706 | 3.151155 | 3.561145 | 4.11676 | 1.225383 | 1.391029 | 2.965417 | 2.558763 | 0.813962 | 2.554611 | 0.596726 | 2.363597 | 1.043399 | 0.9278 | 2.639443 | 1.198094 | 1.809553 | 1.505896 | 0.514893 | 1.758872 | 1.600286 |
| **CD36** | 701.127 | 486.6583 | 359.023 | 545.8663 | 442.2121 | 508.3534 | 551.6459 | 535.4923 | 503.5526 | 768.0431 | 738.6295 | 604.7736 | 413.2083 | 302.5402 | 409.6902 | 246.7639 | 321.9467 | 428.1176 | 364.6199 | 360.5535 | 502.9691 | 355.2762 | 362.3275 | 377.2673 |
| **CD3D** | 3.18212 | 5.122719 | 10.5595 | 1.199706 | 2.10077 | 8.012575 | 1.02919 | 3.676148 | 1.391029 | 0.741354 | 5.970447 | 4.069809 | 1.915958 | 2.983631 | 4.727195 | 2.608498 | 4.639001 | 3.167331 | 5.19174 | 4.976272 | 2.509826 | 4.634038 | 3.517743 | 2.400428 |
| **CD3E** | 23.33554 | 18.44179 | 24.63883 | 17.99559 | 11.55423 | 18.69601 | 33.96327 | 24.50765 | 18.08338 | 11.12031 | 27.29347 | 17.0932 | 6.386526 | 4.773809 | 7.878658 | 5.738695 | 3.711201 | 3.69522 | 5.99047 | 7.690601 | 5.521617 | 7.208504 | 3.957461 | 4.400786 |
| **CD3EAP** | 18.03201 | 11.26998 | 10.5595 | 16.79589 | 24.15885 | 16.02515 | 15.43785 | 6.126914 | 20.16992 | 16.30979 | 16.2055 | 33.37243 | 8.302484 | 8.950892 | 7.878658 | 7.303793 | 5.566801 | 8.974105 | 9.984116 | 4.523883 | 15.05896 | 11.84254 | 9.234075 | 12.40221 |
| **CD4** | 833.7153 | 1219.207 | 929.236 | 1758.769 | 624.979 | 1365.699 | 1532.464 | 1591.772 | 1153.163 | 1112.773 | 1301.557 | 1302.339 | 395.326 | 282.8482 | 324.6007 | 336.4962 | 74.22402 | 319.3726 | 388.5818 | 364.1726 | 315.2341 | 326.9571 | 397.0652 | 379.6678 |
| **CD40** | 10.60707 | 23.56451 | 5.27975 | 5.998531 | 22.05808 | 16.91544 | 22.64218 | 7.352296 | 15.30132 | 18.53386 | 7.676288 | 12.20943 | 16.60497 | 14.91815 | 12.60585 | 14.60759 | 21.33941 | 25.86654 | 28.35489 | 19.90509 | 11.5452 | 14.9319 | 14.95041 | 19.20343 |
| **CD40LG** | 8.485652 | 10.24544 | 5.27975 | 3.599119 | 8.403079 | 9.793148 | 5.14595 | 2.450765 | 5.564117 | 5.18948 | 4.264605 | 3.255847 | 2.554611 | 1.790178 | 3.939329 | 2.086798 | 2.783401 | 6.862551 | 4.393011 | 2.71433 | 3.011791 | 2.574466 | 3.078025 | 2.000357 |
| **CD44** | 676.7308 | 368.8358 | 410.0606 | 781.0087 | 447.464 | 594.7112 | 768.805 | 767.0896 | 422.8729 | 695.3903 | 695.9835 | 755.3565 | 1066.55 | 738.1502 | 906.8336 | 966.1875 | 672.6552 | 891.0759 | 961.2707 | 1038.231 | 907.553 | 962.3352 | 947.592 | 1239.821 |
| **CD45R0** | 7.424946 | 8.19635 | 5.27975 | 1.199706 | 9.453464 | 9.793148 | 5.14595 | 4.901531 | 0.695515 | 3.706772 | 5.970447 | 7.325656 | 501.981 | 267.93 | 390.7815 | 469.0079 | 222.6721 | 453.4563 | 438.5024 | 494.4604 | 442.7333 | 427.8762 | 438.3987 | 560.5 |
| **CD45RA** | 316.0905 | 272.5287 | 295.666 | 367.1101 | 228.9839 | 332.0767 | 442.5517 | 321.0503 | 385.3151 | 375.1253 | 379.5498 | 357.3292 | 26.82341 | 60.26934 | 14.96945 | 20.86798 | 34.32861 | 17.94821 | 35.94282 | 20.35747 | 15.56092 | 24.19998 | 28.58166 | 32.00571 |
| **CD45RB** | 27.57837 | 24.58905 | 17.59917 | 44.38913 | 32.56193 | 42.73374 | 42.19679 | 37.98686 | 34.77573 | 30.39553 | 45.20481 | 50.46563 | 155.1926 | 159.3259 | 87.45311 | 147.1193 | 110.4082 | 153.6156 | 208.069 | 142.0499 | 181.2094 | 162.1913 | 178.0857 | 164.8294 |
| **CD46** | 186.6843 | 183.3933 | 154.8727 | 277.1321 | 151.2554 | 213.6687 | 250.0932 | 198.512 | 224.6512 | 261.6981 | 249.0529 | 265.3515 | 178.1841 | 136.6503 | 147.3309 | 195.1156 | 77.93522 | 148.8646 | 175.3211 | 151.5501 | 152.0954 | 158.0722 | 183.8021 | 206.4368 |
| **CD48** | 153.8024 | 62.49717 | 181.2714 | 140.3656 | 213.2281 | 235.9258 | 123.5028 | 328.4026 | 196.8306 | 303.9553 | 324.9629 | 478.6095 | 860.9038 | 699.363 | 722.473 | 663.6018 | 480.6005 | 776.5241 | 990.025 | 924.2293 | 1046.095 | 734.2376 | 1065.436 | 1142.204 |
| **CD5** | 15.9106 | 9.220894 | 10.5595 | 11.99706 | 17.85654 | 10.68343 | 24.70056 | 20.83151 | 9.04169 | 7.413543 | 11.08797 | 15.46527 | 48.5376 | 25.0625 | 19.69665 | 25.56328 | 31.54521 | 29.03387 | 42.73202 | 43.88167 | 29.61595 | 24.71487 | 42.21292 | 41.20736 |
| **CD53** | 988.5785 | 755.0888 | 774.3633 | 812.2011 | 744.7229 | 925.0073 | 1178.423 | 927.6147 | 1028.666 | 986.0012 | 1113.062 | 1141.988 | 742.753 | 600.9032 | 590.8994 | 573.8695 | 562.2469 | 679.3926 | 734.0322 | 729.25 | 765.9989 | 712.6121 | 779.6198 | 798.1425 |
| **CD55** | 18.03201 | 21.51542 | 5.27975 | 19.1953 | 17.85654 | 29.37944 | 26.75894 | 19.60612 | 20.16992 | 27.43011 | 27.29347 | 23.60489 | 9.57979 | 2.386905 | 7.090792 | 4.695296 | 5.566801 | 5.806774 | 4.393011 | 6.333436 | 5.019652 | 4.634038 | 9.234075 | 10.80193 |
| **CD58** | 40.30685 | 22.53996 | 26.39875 | 23.99412 | 30.46116 | 32.0503 | 29.84651 | 29.40919 | 25.73404 | 31.13688 | 28.99931 | 37.44224 | 164.1337 | 158.1324 | 153.6338 | 183.1165 | 125.253 | 125.6375 | 140.9757 | 177.3362 | 124.9893 | 145.1999 | 171.49 | 176.0314 |
| **CD59** | 80.61369 | 60.44808 | 51.03758 | 68.38325 | 60.92233 | 88.13833 | 79.24764 | 60.04375 | 55.64117 | 63.75647 | 58.85155 | 83.83806 | 114.9575 | 59.67261 | 84.30164 | 73.55963 | 64.01822 | 74.43229 | 103.8348 | 94.09677 | 93.86749 | 97.82969 | 83.98611 | 121.6217 |
| **CD6** | 2.121413 | 7.171807 | 1.759917 | 3.599119 | 5.251925 | 3.561145 | 3.08757 | 2.450765 | 4.173088 | 3.706772 | 1.705842 | 1.627924 | 7.025179 | 5.967261 | 5.515061 | 3.651897 | 7.422402 | 5.278885 | 5.99047 | 8.14299 | 5.521617 | 5.663824 | 7.475204 | 5.601 |
| **CD7** | 31.8212 | 40.98175 | 21.119 | 31.19236 | 16.80616 | 16.91544 | 41.1676 | 20.83151 | 16.69235 | 24.46469 | 36.6756 | 20.34904 | 3.831916 | 2.983631 | 5.515061 | 4.173596 | 8.350202 | 4.223108 | 3.993646 | 3.166718 | 3.513756 | 5.148931 | 4.397179 | 5.200928 |
| **CD70** | 3.18212 | 1.024544 | 1.759917 | 2.399412 | 4.20154 | 0.890286 | 2.05838 | 1.225383 | 2.086544 | 2.965417 | 1.705842 | 3.255847 | 3.193263 | 1.193452 | 2.363597 | 1.565099 | 2.783401 | 0.527889 | 1.198094 | 1.357165 | 1.505896 | 2.574466 | 0.879436 | 0.800143 |
| **CD74** | 15024.91 | 13614.14 | 11545.05 | 18914.57 | 12546.85 | 15837.3 | 19493.89 | 17081.84 | 13833.09 | 16210.45 | 15940.24 | 14841.78 | 26979.88 | 20461.74 | 23639.13 | 21851.38 | 15099.02 | 19906.68 | 23911.96 | 24906.24 | 22359.54 | 21336.66 | 24572.75 | 22944.9 |
| **CD79A** | 68.94592 | 43.03084 | 59.83717 | 56.38619 | 37.81386 | 37.39202 | 55.57626 | 67.39605 | 58.42323 | 54.86022 | 81.02749 | 71.62864 | 5.109221 | 5.967261 | 10.24226 | 6.260394 | 2.783401 | 6.862551 | 7.587928 | 6.785825 | 5.019652 | 4.119145 | 8.35464 | 6.401143 |
| **CD79B** | 12.72848 | 11.26998 | 15.83925 | 7.198237 | 6.30231 | 8.012575 | 9.262711 | 12.25383 | 9.04169 | 8.896252 | 9.38213 | 8.953579 | 13.41171 | 19.09524 | 21.27238 | 10.43399 | 15.7726 | 31.14542 | 19.96823 | 25.78613 | 17.56878 | 23.17019 | 28.58166 | 14.0025 |
| **CD80** | 3.18212 | 3.073631 | 10.5595 | 4.798825 | 5.251925 | 4.451431 | 6.175141 | 6.126914 | 9.04169 | 5.18948 | 1.705842 | 3.255847 | 3.831916 | 3.580357 | 7.878658 | 6.782094 | 2.783401 | 7.918328 | 5.99047 | 9.500155 | 3.513756 | 5.663824 | 6.595768 | 2.400428 |
| **CD81** | 322.4548 | 264.3323 | 168.952 | 280.7312 | 190.1197 | 270.647 | 279.9397 | 301.4442 | 232.3019 | 226.8544 | 243.0825 | 265.3515 | 140.5036 | 134.2634 | 103.2104 | 105.905 | 67.72942 | 120.3586 | 113.8189 | 158.7883 | 78.80853 | 126.1488 | 127.0785 | 141.2252 |
| **CD82** | 12.72848 | 6.147263 | 15.83925 | 8.397943 | 12.60462 | 7.122289 | 10.2919 | 8.577679 | 9.04169 | 5.18948 | 10.23505 | 13.83735 | 3.831916 | 2.386905 | 2.363597 | 6.260394 | 3.711201 | 3.167331 | 3.194917 | 3.619107 | 5.019652 | 6.178717 | 4.397179 | 3.600643 |
| **CD83** | 12.72848 | 24.58905 | 14.07933 | 13.19677 | 22.05808 | 20.47658 | 15.43785 | 15.92998 | 11.82375 | 16.30979 | 5.970447 | 26.04678 | 106.655 | 104.4271 | 126.8464 | 187.2901 | 127.1086 | 51.20519 | 111.0234 | 189.0983 | 53.71027 | 95.77012 | 108.6103 | 140.025 |
| **CD86** | 37.12473 | 21.51542 | 26.39875 | 23.99412 | 16.80616 | 25.8183 | 33.96327 | 20.83151 | 34.77573 | 25.20605 | 29.85223 | 37.44224 | 205.6461 | 229.7396 | 179.6334 | 259.2847 | 116.9028 | 201.1255 | 243.6124 | 211.7177 | 205.3038 | 204.9275 | 266.9088 | 236.8423 |
| **CD8A** | 3.18212 | 3.073631 | 7.039667 | 1.199706 | 4.20154 | 5.341717 | 3.08757 | 6.126914 | 1.391029 | 2.965417 | 2.558763 | 4.069809 | 5.747874 | 5.370535 | 3.151463 | 4.173596 | 7.422402 | 6.334663 | 5.19174 | 2.71433 | 3.011791 | 3.089359 | 3.517743 | 3.600643 |
| **CD8B** | 1.060707 | 5.122719 | 1.759917 | 2.399412 | 4.20154 | 1.780572 | 1.02919 | 1.225383 | 2.782058 | 5.18948 | 2.558763 | 2.441885 | 3.193263 | 1.193452 | 0.787866 | 1.565099 | 2.783401 | 2.111554 | 2.396188 | 3.166718 | 3.011791 | 2.059572 | 2.198589 | 0.400071 |
| **CD9** | 14.84989 | 13.31907 | 12.31942 | 14.39647 | 16.80616 | 15.13486 | 11.32109 | 18.38074 | 6.259631 | 4.448126 | 9.38213 | 9.767541 | 5.109221 | 35.80357 | 3.939329 | 4.173596 | 13.917 | 3.167331 | 3.594282 | 2.261942 | 3.011791 | 13.38722 | 2.198589 | 4.800857 |
| **CD96** | 10.60707 | 13.31907 | 14.07933 | 10.79736 | 17.85654 | 16.91544 | 9.262711 | 11.02844 | 13.21478 | 10.37896 | 9.38213 | 9.767541 | 10.21844 | 13.12798 | 11.81799 | 7.303793 | 11.1336 | 8.974105 | 7.587928 | 9.047766 | 11.5452 | 7.208504 | 7.475204 | 5.200928 |
| **CD97** | 123.042 | 89.13531 | 117.9144 | 208.7489 | 120.7943 | 157.5807 | 170.8456 | 133.5667 | 148.8401 | 128.2543 | 174.8488 | 183.1414 | 253.5451 | 226.7559 | 184.3606 | 263.98 | 137.3144 | 206.9323 | 295.1305 | 194.0746 | 236.9276 | 251.7827 | 331.5473 | 307.2548 |
| **CD99** | 1498.778 | 1268.385 | 1006.672 | 1690.386 | 1028.327 | 1102.174 | 1490.267 | 1407.965 | 1348.603 | 1280.319 | 1392.82 | 1474.899 | 814.2821 | 655.802 | 645.2621 | 720.467 | 514.9291 | 516.8029 | 710.0703 | 635.1532 | 748.4301 | 756.378 | 853.0527 | 956.1707 |
| **CDH5** | 5.303533 | 5.122719 | 5.27975 | 1.199706 | 1.050385 | 0.890286 | 1.02919 | 4.901531 | 0.695515 | 5.930835 | 1.705842 | 1.627924 | 1.915958 | 2.983631 | 1.575732 | 1.043399 | 0.9278 | 3.69522 | 1.996823 | 3.166718 | 1.505896 | 1.544679 | 0.439718 | 0.800143 |
| **CDKN1A** | 39.24614 | 64.54626 | 84.476 | 89.97796 | 67.22464 | 70.33261 | 32.93408 | 75.97373 | 45.90396 | 53.37751 | 40.08728 | 68.37279 | 33.20994 | 152.1652 | 128.4221 | 205.0279 | 127.1086 | 72.84862 | 26.35807 | 187.2888 | 37.14542 | 52.0042 | 35.61715 | 56.41007 |
| **CEACAM1** | 4.242826 | 2.049088 | 5.27975 | 2.399412 | 6.30231 | 2.670858 | 3.08757 | 7.352296 | 3.477573 | 4.448126 | 3.411684 | 4.883771 | 5.747874 | 4.177083 | 3.151463 | 4.173596 | 0.9278 | 3.69522 | 3.594282 | 2.261942 | 3.011791 | 0.514893 | 2.638307 | 2.8005 |
| **CEACAM6** | 4.242826 | 7.171807 | 10.5595 | 3.599119 | 5.251925 | 4.451431 | 4.11676 | 7.352296 | 4.868602 | 4.448126 | 5.970447 | 5.697732 | 3.831916 | 4.773809 | 4.727195 | 4.173596 | 6.494602 | 5.278885 | 3.194917 | 3.166718 | 5.521617 | 6.178717 | 2.638307 | 4.800857 |
| **CEACAM8** | 3.18212 | 6.147263 | 7.039667 | 3.599119 | 5.251925 | 7.122289 | 7.204331 | 3.676148 | 5.564117 | 4.448126 | 5.117526 | 1.627924 | 1.915958 | 0.596726 | 3.939329 | 1.043399 | 2.783401 | 2.111554 | 3.194917 | 4.071495 | 1.00393 | 3.604252 | 2.638307 | 1.600286 |
| **CEBPB** | 13.78918 | 14.34361 | 7.039667 | 9.597649 | 13.655 | 14.24458 | 6.175141 | 7.352296 | 11.12823 | 7.413543 | 11.08797 | 9.767541 | 105.3777 | 295.9762 | 184.3606 | 527.4382 | 222.6721 | 234.3825 | 188.5001 | 204.0271 | 202.292 | 198.7487 | 249.32 | 248.4443 |
| **CFB** | 8.485652 | 6.147263 | 14.07933 | 3.599119 | 4.20154 | 10.68343 | 2.05838 | 4.901531 | 6.259631 | 8.896252 | 4.264605 | 4.069809 | 5.109221 | 6.563988 | 2.363597 | 3.651897 | 2.783401 | 3.69522 | 3.594282 | 3.619107 | 4.517687 | 7.723397 | 2.198589 | 4.800857 |
| **CFD** | 5.303533 | 6.147263 | 5.27975 | 5.998531 | 6.30231 | 7.122289 | 9.262711 | 1.225383 | 6.955146 | 7.413543 | 2.558763 | 4.883771 | 7.025179 | 19.69196 | 7.878658 | 10.95569 | 8.350202 | 22.69921 | 15.17586 | 13.11926 | 20.07861 | 29.34891 | 15.82984 | 20.40364 |
| **CFH** | 3.18212 | 7.171807 | 1.759917 | 1.199706 | 4.20154 | 3.561145 | 2.05838 | 3.676148 | 3.477573 | 4.448126 | 1.705842 | 2.441885 | 3.831916 | 1.790178 | 4.727195 | 2.608498 | 3.711201 | 2.639443 | 1.597459 | 2.261942 | 3.011791 | 1.544679 | 3.078025 | 2.000357 |
| **CFI** | 7.424946 | 7.171807 | 17.59917 | 3.599119 | 6.30231 | 2.670858 | 3.08757 | 6.126914 | 9.04169 | 9.637606 | 5.117526 | 7.325656 | 6.386526 | 7.75744 | 8.666524 | 5.738695 | 8.350202 | 10.02988 | 5.99047 | 5.881048 | 4.015721 | 5.663824 | 6.595768 | 8.801571 |
| **CFP** | 2.121413 | 2.049088 | 7.039667 | 5.998531 | 5.251925 | 10.68343 | 3.08757 | 6.126914 | 7.650661 | 5.930835 | 5.117526 | 10.5815 | 210.1167 | 156.939 | 197.7543 | 214.4185 | 128.9642 | 163.6455 | 212.0626 | 213.0749 | 198.2762 | 203.3828 | 237.4477 | 297.2531 |
| **CHUK** | 58.33886 | 54.30082 | 61.59708 | 71.98237 | 78.77887 | 69.44232 | 95.71468 | 66.17067 | 85.5483 | 86.73846 | 93.8213 | 84.65202 | 43.42838 | 44.15773 | 54.36274 | 77.21153 | 39.89541 | 49.62152 | 70.28818 | 53.83421 | 64.25154 | 78.77865 | 66.83712 | 74.81335 |
| **CIITA** | 13.78918 | 25.61359 | 12.31942 | 45.58883 | 29.41078 | 19.5863 | 56.60545 | 25.73304 | 27.12507 | 71.17001 | 27.29347 | 37.44224 | 99.62981 | 64.44642 | 118.9677 | 127.8164 | 42.67881 | 66.51396 | 151.3592 | 71.02497 | 75.29478 | 86.50204 | 159.1779 | 134.8241 |
| **CISH** | 5.303533 | 6.147263 | 3.519833 | 14.39647 | 14.70539 | 6.232003 | 6.175141 | 12.25383 | 4.868602 | 2.965417 | 2.558763 | 5.697732 | 1.277305 | 8.354166 | 4.727195 | 6.260394 | 2.783401 | 2.639443 | 1.996823 | 2.261942 | 5.521617 | 2.574466 | 3.078025 | 5.200928 |
| **CLEC4A** | 70.00663 | 57.37445 | 65.11692 | 99.57561 | 67.22464 | 66.77146 | 167.758 | 60.04375 | 100.1541 | 68.94595 | 116.8502 | 65.9309 | 488.5693 | 391.4523 | 350.6003 | 403.7954 | 247.7227 | 407.0021 | 458.4706 | 453.2931 | 509.9966 | 529.3101 | 514.9096 | 542.0968 |
| **CLEC4E** | 2.121413 | 5.122719 | 8.799583 | 2.399412 | 5.251925 | 1.780572 | 4.11676 | 2.450765 | 6.955146 | 1.482709 | 1.705842 | 7.325656 | 6.386526 | 7.75744 | 7.090792 | 13.04249 | 7.422402 | 5.806774 | 5.591105 | 9.500155 | 14.55699 | 10.81276 | 10.11351 | 11.60207 |
| **CLEC5A** | 8.485652 | 1.024544 | 3.519833 | 4.798825 | 2.10077 | 7.122289 | 4.11676 | 3.676148 | 2.782058 | 5.18948 | 0.852921 | 4.069809 | 7.663832 | 7.160714 | 3.151463 | 3.651897 | 3.711201 | 4.750997 | 2.396188 | 5.42866 | 2.007861 | 4.119145 | 3.957461 | 3.600643 |
| **CLEC6A** | 2.121413 | 4.098175 | 1.759917 | 2.399412 | 2.10077 | 0.890286 | 2.05838 | 1.225383 | 3.477573 | 1.482709 | 2.558763 | 2.441885 | 3.193263 | 5.967261 | 7.090792 | 7.303793 | 6.494602 | 7.918328 | 3.194917 | 4.071495 | 4.517687 | 5.148931 | 3.078025 | 4.800857 |
| **CLEC7A** | 4.242826 | 5.122719 | 7.039667 | 2.399412 | 6.30231 | 6.232003 | 5.14595 | 2.450765 | 5.564117 | 6.672189 | 1.705842 | 7.325656 | 54.28547 | 69.22023 | 68.54433 | 64.69074 | 82.57422 | 57.01196 | 113.0202 | 85.50139 | 45.17687 | 76.20418 | 105.972 | 66.81193 |
| **CLU** | 2.121413 | 2.049088 | 1.759917 | 3.599119 | 4.20154 | 0.890286 | 6.175141 | 4.901531 | 4.173088 | 3.706772 | 0.852921 | 3.255847 | 3.193263 | 4.177083 | 4.727195 | 5.738695 | 7.422402 | 4.750997 | 4.393011 | 4.523883 | 3.011791 | 1.029786 | 4.397179 | 3.600643 |
| **CMKLR1** | 252.4481 | 188.5161 | 218.2297 | 580.6578 | 204.8251 | 385.4939 | 448.7269 | 241.4004 | 428.437 | 532.2924 | 427.3134 | 420.0043 | 3.831916 | 4.177083 | 7.090792 | 6.782094 | 4.639001 | 4.750997 | 1.996823 | 0.452388 | 5.521617 | 5.148931 | 3.517743 | 2.400428 |
| **CR1** | 4.242826 | 9.220894 | 7.039667 | 3.599119 | 9.453464 | 7.122289 | 1.02919 | 3.676148 | 4.173088 | 2.224063 | 3.411684 | 7.325656 | 26.18476 | 25.65922 | 18.90878 | 21.38968 | 14.8448 | 21.11554 | 17.57204 | 19.00031 | 16.56485 | 21.11062 | 23.30505 | 20.40364 |
| **CR2** | 6.364239 | 2.049088 | 12.31942 | 4.798825 | 9.453464 | 3.561145 | 5.14595 | 6.126914 | 1.391029 | 5.18948 | 1.705842 | 4.069809 | 3.831916 | 3.580357 | 3.151463 | 2.086798 | 6.494602 | 3.167331 | 1.597459 | 3.166718 | 2.007861 | 4.634038 | 3.517743 | 1.200214 |
| **CRADD** | 16.9713 | 12.29453 | 19.35908 | 13.19677 | 9.453464 | 16.91544 | 23.67137 | 23.28227 | 18.77889 | 14.08573 | 12.79381 | 20.34904 | 8.941137 | 8.950892 | 6.302927 | 13.56419 | 5.566801 | 6.334663 | 9.185387 | 11.30971 | 12.04716 | 10.29786 | 7.914922 | 10.40186 |
| **CSF1** | 4.242826 | 2.049088 | 3.519833 | 3.599119 | 2.10077 | 1.780572 | 2.05838 | 3.676148 | 3.477573 | 3.706772 | 4.264605 | 2.441885 | 0.638653 | 1.193452 | 3.151463 | 2.608498 | 1.8556 | 5.806774 | 1.597459 | 2.71433 | 0.501965 | 3.089359 | 0.879436 | 3.200571 |
| **CSF1R** | 14.84989 | 8.19635 | 7.039667 | 13.19677 | 13.655 | 4.451431 | 21.61299 | 7.352296 | 6.259631 | 3.706772 | 3.411684 | 4.069809 | 29.37802 | 32.81994 | 30.72677 | 38.60577 | 25.05061 | 30.61754 | 33.54663 | 29.85763 | 27.60808 | 34.49784 | 41.7732 | 30.00536 |
| **CSF2** | 2.121413 | 3.073631 | 1.759917 | 2.399412 | 2.10077 | 2.670858 | 1.02919 | 2.450765 | 0.695515 | 1.482709 | 1.705842 | 0.813962 | 4.470568 | 0.596726 | 1.575732 | 0.5217 | 1.8556 | 2.639443 | 2.396188 | 1.809553 | 1.505896 | 3.089359 | 2.638307 | 1.200214 |
| **CSF2RB** | 619.4526 | 621.8981 | 487.4969 | 783.4081 | 480.0259 | 588.4792 | 748.2212 | 665.3828 | 559.8893 | 576.0323 | 559.5161 | 559.1917 | 93.88194 | 84.13839 | 122.1192 | 126.2513 | 97.41903 | 85.51794 | 113.4196 | 137.9784 | 95.37338 | 134.3871 | 124.0004 | 112.02 |
| **CSF3R** | 14.84989 | 12.29453 | 10.5595 | 11.99706 | 13.655 | 13.35429 | 13.37947 | 14.70459 | 10.43272 | 8.896252 | 8.529209 | 15.46527 | 172.4362 | 142.0208 | 180.4213 | 187.2901 | 104.8414 | 124.5817 | 180.9122 | 143.8595 | 142.5581 | 153.4381 | 157.419 | 189.6338 |
| **CTLA4-TM** | 3.18212 | 4.098175 | 3.519833 | 1.199706 | 5.251925 | 2.670858 | 5.14595 | 3.676148 | 2.086544 | 2.965417 | 1.705842 | 4.069809 | 2.554611 | 2.386905 | 0.787866 | 4.173596 | 2.783401 | 1.583666 | 1.597459 | 4.071495 | 2.007861 | 4.634038 | 3.517743 | 2.8005 |
| **CTLA4_all** | 5.303533 | 4.098175 | 5.27975 | 2.399412 | 1.050385 | 2.670858 | 1.02919 | 2.450765 | 1.391029 | 0.741354 | 5.970447 | 0.813962 | 3.831916 | 7.160714 | 2.363597 | 4.173596 | 3.711201 | 3.69522 | 2.795553 | 4.071495 | 2.509826 | 2.574466 | 1.319154 | 2.400428 |
| **CTNNB1** | 268.3587 | 236.6696 | 175.9917 | 291.5286 | 205.8754 | 323.1739 | 402.4133 | 287.9649 | 324.1098 | 375.1253 | 374.4323 | 463.1442 | 298.2508 | 288.8155 | 284.4196 | 404.8388 | 213.3941 | 303.008 | 355.0352 | 309.886 | 304.1909 | 332.1061 | 377.2779 | 416.4743 |
| **CTSC** | 1525.296 | 1316.539 | 1054.19 | 1890.737 | 1173.28 | 1144.908 | 1524.231 | 1604.026 | 1073.875 | 1114.256 | 1606.05 | 1418.735 | 477.0735 | 346.6979 | 297.0254 | 390.7529 | 294.1127 | 407.53 | 444.8922 | 394.0302 | 392.0348 | 445.8974 | 408.0582 | 535.2955 |
| **CTSG** | 1.060707 | 1.024544 | 5.27975 | 3.599119 | 1.050385 | 1.780572 | 2.05838 | 1.225383 | 4.868602 | 4.448126 | 0.852921 | 0.813962 | 5.109221 | 1.790178 | 1.575732 | 2.086798 | 1.8556 | 1.055777 | 2.396188 | 4.523883 | 0.501965 | 3.604252 | 1.319154 | 1.200214 |
| **CTSS** | 1922 | 1712.013 | 1393.854 | 1969.918 | 1403.314 | 1963.971 | 2098.519 | 2302.494 | 1675.495 | 1914.918 | 2023.128 | 2113.859 | 2633.165 | 2883.381 | 1977.543 | 2641.365 | 1389.845 | 3118.238 | 2989.644 | 2932.381 | 2601.686 | 2920.989 | 2709.102 | 2912.52 |
| **CUL9** | 31.8212 | 36.88358 | 22.87892 | 25.19383 | 17.85654 | 24.03773 | 23.67137 | 37.98686 | 24.34301 | 34.1023 | 18.76426 | 26.86074 | 17.24362 | 11.3378 | 15.75732 | 19.82458 | 16.7004 | 17.94821 | 20.3676 | 15.3812 | 12.54913 | 15.96169 | 18.90787 | 16.803 |
| **CX3CL1** | 4.242826 | 6.147263 | 12.31942 | 4.798825 | 13.655 | 3.561145 | 5.14595 | 14.70459 | 6.259631 | 3.706772 | 5.970447 | 4.069809 | 5.747874 | 2.983631 | 7.878658 | 7.303793 | 11.1336 | 9.501994 | 4.393011 | 3.166718 | 3.513756 | 5.148931 | 3.957461 | 4.400786 |
| **CX3CR1** | 19.09272 | 12.29453 | 28.15867 | 10.79736 | 19.95731 | 48.96574 | 32.93408 | 36.76148 | 29.21161 | 14.82709 | 23.02887 | 41.51205 | 197.9823 | 102.6369 | 193.815 | 161.2052 | 206.8995 | 314.6216 | 423.3265 | 221.6703 | 463.3139 | 367.1188 | 343.8594 | 471.6842 |
| **CXCL1** | 1.060707 | 4.098175 | 12.31942 | 1.199706 | 5.251925 | 5.341717 | 5.14595 | 2.450765 | 6.259631 | 8.154897 | 4.264605 | 2.441885 | 3.831916 | 1.790178 | 4.727195 | 2.608498 | 1.8556 | 2.639443 | 4.393011 | 4.071495 | 5.019652 | 3.089359 | 3.078025 | 2.000357 |
| **CXCL10** | 4.242826 | 3.073631 | 1.759917 | 2.399412 | 3.151155 | 2.670858 | 4.11676 | 2.450765 | 2.782058 | 3.706772 | 3.411684 | 3.255847 | 5.747874 | 9.547618 | 7.090792 | 8.347192 | 4.639001 | 15.83666 | 2.795553 | 6.785825 | 4.517687 | 4.634038 | 5.716332 | 4.400786 |
| **CXCL11** | 8.485652 | 4.098175 | 1.759917 | 3.599119 | 5.251925 | 4.451431 | 1.02919 | 2.450765 | 6.955146 | 5.18948 | 3.411684 | 4.069809 | 3.831916 | 6.563988 | 11.03012 | 4.173596 | 5.566801 | 2.639443 | 4.393011 | 4.071495 | 4.517687 | 6.69361 | 3.517743 | 4.000714 |
| **CXCL12** | 5.303533 | 6.147263 | 10.5595 | 1.199706 | 6.30231 | 3.561145 | 2.05838 | 3.676148 | 4.173088 | 2.224063 | 6.823368 | 4.069809 | 1.915958 | 5.967261 | 6.302927 | 3.130197 | 7.422402 | 3.69522 | 2.795553 | 3.166718 | 2.509826 | 3.089359 | 2.198589 | 1.600286 |
| **CXCL13** | 5.303533 | 4.098175 | 3.519833 | 9.597649 | 4.20154 | 4.451431 | 1.02919 | 2.450765 | 2.782058 | 5.930835 | 5.117526 | 1.627924 | 7.025179 | 2.386905 | 3.151463 | 4.173596 | 3.711201 | 4.750997 | 3.594282 | 3.619107 | 3.011791 | 3.089359 | 3.078025 | 2.8005 |
| **CXCL2** | 5.303533 | 8.19635 | 1.759917 | 3.599119 | 6.30231 | 3.561145 | 3.08757 | 3.676148 | 4.868602 | 0.741354 | 3.411684 | 7.325656 | 6.386526 | 2.983631 | 2.363597 | 4.695296 | 11.1336 | 3.167331 | 3.993646 | 3.166718 | 5.019652 | 5.148931 | 3.517743 | 2.000357 |
| **CXCL9** | 3.18212 | 1.024544 | 7.039667 | 3.599119 | 6.30231 | 8.012575 | 2.05838 | 3.676148 | 4.173088 | 3.706772 | 1.705842 | 0.813962 | 3.193263 | 8.950892 | 5.515061 | 8.347192 | 4.639001 | 10.02988 | 6.789199 | 4.523883 | 4.015721 | 5.663824 | 6.595768 | 4.800857 |
| **CXCR1** | 1.060707 | 3.073631 | 3.519833 | 1.199706 | 5.251925 | 0.890286 | 2.05838 | 1.225383 | 2.782058 | 2.965417 | 2.558763 | 1.627924 | 1.277305 | 0.596726 | 2.363597 | 2.086798 | 1.8556 | 2.639443 | 2.396188 | 0.452388 | 1.00393 | 1.544679 | 1.758872 | 2.000357 |
| **CXCR2** | 20.15342 | 18.44179 | 14.07933 | 27.59324 | 16.80616 | 26.70858 | 47.34274 | 12.25383 | 34.08022 | 28.91282 | 17.91134 | 44.7679 | 10.21844 | 8.950892 | 4.727195 | 8.347192 | 15.7726 | 12.14144 | 17.97141 | 9.952543 | 14.55699 | 21.62551 | 20.22702 | 14.80264 |
| **CXCR3** | 370.1866 | 300.1913 | 242.8685 | 557.8634 | 353.9797 | 665.9341 | 568.1129 | 289.1903 | 522.3315 | 292.835 | 442.666 | 424.888 | 14.05036 | 7.160714 | 11.81799 | 6.782094 | 10.2058 | 8.974105 | 10.38348 | 11.30971 | 17.06682 | 7.723397 | 15.39013 | 16.00286 |
| **CXCR4** | 644.9096 | 1216.133 | 536.7746 | 1394.059 | 1062.99 | 1706.679 | 990.0809 | 1050.153 | 735.1589 | 642.0128 | 595.3388 | 939.3119 | 225.4444 | 336.5535 | 248.1777 | 321.8886 | 167.004 | 1099.064 | 246.8073 | 175.0743 | 257.5081 | 293.4891 | 408.4979 | 302.0539 |
| **CXCR6** | 6.364239 | 1.024544 | 7.039667 | 2.399412 | 1.050385 | 1.780572 | 1.02919 | 1.225383 | 2.086544 | 0.741354 | 2.558763 | 0.813962 | 0.638653 | 2.386905 | 1.575732 | 1.565099 | 2.783401 | 3.167331 | 1.597459 | 1.357165 | 1.505896 | 1.544679 | 0.879436 | 0.800143 |
| **CYBB** | 1266.484 | 1095.237 | 955.6348 | 1505.631 | 1091.35 | 1432.47 | 1563.34 | 1444.726 | 1231.756 | 1524.966 | 1672.578 | 1590.481 | 397.2419 | 427.8526 | 301.7526 | 350.5821 | 310.8131 | 563.2571 | 488.423 | 376.8395 | 420.6468 | 378.4464 | 425.2072 | 410.0732 |
| **DDX58 (RIG-1)** | 8.485652 | 1.024544 | 1.759917 | 2.399412 | 6.30231 | 4.451431 | 1.02919 | 1.225383 | 1.391029 | 2.965417 | 3.411684 | 4.069809 | 11.49575 | 11.3378 | 11.03012 | 16.17269 | 9.278002 | 16.36455 | 19.96823 | 6.333436 | 14.05503 | 13.90211 | 13.19154 | 18.40328 |
| **DEFB1** | 10.60707 | 8.19635 | 14.07933 | 11.99706 | 9.453464 | 7.122289 | 4.11676 | 4.901531 | 10.43272 | 7.413543 | 6.823368 | 11.39546 | 7.663832 | 7.75744 | 9.45439 | 4.173596 | 12.9892 | 7.39044 | 5.99047 | 8.595378 | 4.517687 | 3.604252 | 6.595768 | 3.600643 |
| **DEFB103A** | 6.364239 | 5.122719 | 7.039667 | 5.998531 | 8.403079 | 5.341717 | 2.05838 | 1.225383 | 2.782058 | 7.413543 | 0.852921 | 3.255847 | 4.470568 | 3.580357 | 9.45439 | 4.695296 | 9.278002 | 3.69522 | 3.594282 | 3.619107 | 3.513756 | 3.604252 | 4.836897 | 3.600643 |
| **DEFB103B** | 1.060707 | 2.049088 | 3.519833 | 3.599119 | 7.352695 | 2.670858 | 2.05838 | 3.676148 | 7.650661 | 2.224063 | 3.411684 | 1.627924 | 1.915958 | 5.370535 | 3.151463 | 2.086798 | 2.783401 | 4.750997 | 2.795553 | 3.619107 | 1.505896 | 3.604252 | 4.397179 | 2.000357 |
| **DEFB4A** | 8.485652 | 4.098175 | 7.039667 | 2.399412 | 5.251925 | 6.232003 | 3.08757 | 3.676148 | 6.259631 | 1.482709 | 2.558763 | 4.883771 | 3.193263 | 4.177083 | 6.302927 | 3.130197 | 3.711201 | 1.055777 | 2.795553 | 3.619107 | 1.505896 | 1.029786 | 1.758872 | 2.8005 |
| **DPP4** | 80.61369 | 63.52172 | 66.87683 | 91.17767 | 64.07348 | 56.08803 | 124.532 | 98.03062 | 90.4169 | 60.79105 | 63.96907 | 95.23353 | 12.77305 | 7.75744 | 13.39372 | 12.52079 | 7.422402 | 6.862551 | 19.1695 | 7.690601 | 14.55699 | 12.35743 | 13.19154 | 16.40293 |
| **DUSP4** | 7.424946 | 12.29453 | 24.63883 | 5.998531 | 9.453464 | 7.122289 | 3.08757 | 6.126914 | 8.346175 | 2.965417 | 11.94089 | 24.41885 | 3.193263 | 4.773809 | 4.727195 | 4.173596 | 5.566801 | 3.69522 | 2.396188 | 4.523883 | 3.513756 | 2.574466 | 3.517743 | 2.400428 |
| **EBI3** | 7.424946 | 4.098175 | 3.519833 | 8.397943 | 3.151155 | 3.561145 | 4.11676 | 1.225383 | 2.086544 | 2.224063 | 3.411684 | 3.255847 | 3.831916 | 5.370535 | 6.302927 | 3.651897 | 3.711201 | 4.223108 | 4.393011 | 3.166718 | 5.019652 | 4.119145 | 3.957461 | 2.400428 |
| **EDNRB** | 4.242826 | 2.049088 | 1.759917 | 1.199706 | 1.050385 | 2.670858 | 3.08757 | 1.225383 | 2.086544 | 0.741354 | 1.705842 | 2.441885 | 0.638653 | 1.193452 | 3.939329 | 1.043399 | 0.9278 | 1.583666 | 0.798729 | 1.809553 | 0.501965 | 0.514893 | 1.319154 | 1.600286 |
| **EGR1** | 4.242826 | 9.220894 | 7.039667 | 2.399412 | 5.251925 | 4.451431 | 4.11676 | 4.901531 | 4.173088 | 3.706772 | 6.823368 | 5.697732 | 15.96632 | 35.20684 | 45.69622 | 141.3806 | 41.75101 | 26.92232 | 11.98094 | 43.42928 | 39.65525 | 40.67656 | 25.94335 | 31.60564 |
| **EGR2** | 6.364239 | 6.147263 | 1.759917 | 3.599119 | 4.20154 | 8.012575 | 1.02919 | 6.126914 | 5.564117 | 1.482709 | 6.823368 | 5.697732 | 8.941137 | 16.70833 | 25.99957 | 35.47557 | 40.82321 | 13.19721 | 20.76696 | 23.97658 | 17.56878 | 18.02126 | 6.15605 | 34.40614 |
| **ENTPD1** | 2.121413 | 9.220894 | 10.5595 | 1.199706 | 5.251925 | 5.341717 | 1.02919 | 6.126914 | 7.650661 | 5.930835 | 5.117526 | 7.325656 | 17.24362 | 13.12798 | 14.18158 | 14.08589 | 9.278002 | 11.08566 | 22.36442 | 17.64314 | 15.56092 | 12.87233 | 19.34759 | 20.80371 |
| **EOMES** | 9.546359 | 12.29453 | 15.83925 | 4.798825 | 8.403079 | 3.561145 | 8.233521 | 6.126914 | 9.737204 | 9.637606 | 9.38213 | 5.697732 | 8.302484 | 2.983631 | 8.666524 | 2.608498 | 7.422402 | 3.69522 | 6.789199 | 5.42866 | 7.027513 | 6.69361 | 6.15605 | 3.600643 |
| **ETS1** | 327.7583 | 248.9641 | 401.261 | 459.4875 | 274.1505 | 430.8985 | 519.741 | 335.7549 | 372.7958 | 403.2967 | 528.811 | 531.517 | 14.68901 | 9.547618 | 6.302927 | 8.868892 | 16.7004 | 12.14144 | 19.96823 | 12.21448 | 13.05109 | 18.02126 | 16.26956 | 16.40293 |
| **FADD** | 3.18212 | 11.26998 | 8.799583 | 7.198237 | 11.55423 | 8.012575 | 8.233521 | 1.225383 | 9.737204 | 6.672189 | 7.676288 | 11.39546 | 6.386526 | 8.354166 | 7.090792 | 4.173596 | 11.1336 | 5.278885 | 6.789199 | 6.333436 | 8.031443 | 4.634038 | 5.276615 | 6.401143 |
| **FAS** | 2.121413 | 6.147263 | 1.759917 | 4.798825 | 5.251925 | 1.780572 | 2.05838 | 2.450765 | 6.259631 | 3.706772 | 2.558763 | 4.883771 | 10.21844 | 10.14434 | 14.18158 | 6.782094 | 10.2058 | 10.55777 | 13.5784 | 11.30971 | 12.04716 | 9.268076 | 7.475204 | 12.00214 |
| **FCAR** | 1.060707 | 2.049088 | 5.27975 | 3.599119 | 1.050385 | 4.451431 | 5.14595 | 8.577679 | 4.173088 | 2.224063 | 3.411684 | 1.627924 | 2.554611 | 4.177083 | 5.515061 | 4.173596 | 3.711201 | 2.639443 | 1.996823 | 0.904777 | 2.509826 | 3.604252 | 2.638307 | 1.200214 |
| **FCER1A** | 651.2738 | 414.9402 | 418.8602 | 352.7136 | 755.2268 | 533.2814 | 921.1251 | 736.455 | 730.2903 | 1334.438 | 1011.564 | 1411.41 | 4471.846 | 3089.251 | 4024.419 | 3322.183 | 1401.906 | 1991.723 | 4713.302 | 3357.174 | 4679.821 | 4649.485 | 4506.669 | 5931.059 |
| **FCER1G** | 1374.676 | 957.9485 | 1047.15 | 1486.436 | 1093.451 | 1429.8 | 1831.958 | 1371.203 | 1447.366 | 1352.23 | 1268.293 | 1716.645 | 1064.634 | 912.3943 | 808.3503 | 941.6676 | 698.6336 | 921.1655 | 1206.081 | 1083.018 | 1029.029 | 947.9182 | 1040.812 | 1438.657 |
| **FCGR1A/B** | 3.18212 | 2.049088 | 5.27975 | 2.399412 | 8.403079 | 3.561145 | 1.02919 | 1.225383 | 1.391029 | 4.448126 | 4.264605 | 2.441885 | 4.470568 | 4.177083 | 3.151463 | 4.173596 | 11.1336 | 4.223108 | 3.594282 | 4.071495 | 6.023582 | 4.634038 | 1.758872 | 2.8005 |
| **FCGR2A** | 5.303533 | 2.049088 | 7.039667 | 4.798825 | 1.050385 | 8.902862 | 8.233521 | 3.676148 | 4.173088 | 5.18948 | 4.264605 | 6.511694 | 91.96598 | 92.49255 | 96.9075 | 196.6807 | 124.3252 | 131.4442 | 146.1675 | 185.0268 | 88.84784 | 116.3658 | 174.568 | 149.2266 |
| **FCGR2A/C** | 4.242826 | 4.098175 | 8.799583 | 3.599119 | 9.453464 | 14.24458 | 10.2919 | 9.803062 | 4.173088 | 9.637606 | 7.676288 | 7.325656 | 174.9908 | 164.6964 | 160.7246 | 289.0215 | 165.1484 | 196.9024 | 210.4652 | 265.5519 | 163.1387 | 290.9146 | 335.065 | 204.4365 |
| **FCGR2B** | 2.121413 | 2.049088 | 3.519833 | 1.199706 | 1.050385 | 0.890286 | 2.05838 | 3.676148 | 4.173088 | 2.965417 | 2.558763 | 3.255847 | 178.8227 | 122.9256 | 164.664 | 186.2467 | 176.282 | 122.998 | 176.5192 | 273.6949 | 186.731 | 213.6806 | 306.0436 | 168.4301 |
| **FCGR3A/B** | 3.18212 | 2.049088 | 3.519833 | 4.798825 | 2.10077 | 25.8183 | 8.233521 | 4.901531 | 0.695515 | 3.706772 | 3.411684 | 1.627924 | 14.68901 | 18.49851 | 7.878658 | 13.04249 | 18.556 | 10.55777 | 21.96506 | 30.31002 | 9.537338 | 15.96169 | 13.19154 | 51.60921 |
| **FCGRT** | 373.3687 | 322.7313 | 251.6681 | 475.0836 | 252.0924 | 394.3968 | 478.5734 | 486.4769 | 396.4433 | 402.5554 | 392.3436 | 478.6095 | 544.132 | 433.8199 | 517.6279 | 477.3551 | 228.2389 | 379.024 | 583.0724 | 499.8891 | 567.7226 | 527.2505 | 602.4135 | 621.711 |
| **FKBP5** | 194.1093 | 133.1907 | 124.9541 | 190.7533 | 167.0112 | 198.5338 | 235.6845 | 197.2866 | 264.9911 | 257.9913 | 303.6399 | 240.9327 | 159.6632 | 131.2798 | 158.361 | 146.5976 | 174.4264 | 174.2032 | 177.3179 | 152.0025 | 363.4228 | 325.4124 | 252.8378 | 266.8476 |
| **FN1** | 5.303533 | 6.147263 | 5.27975 | 3.599119 | 10.50385 | 2.670858 | 1.02919 | 1.225383 | 1.391029 | 2.224063 | 0.852921 | 3.255847 | 0.638653 | 4.177083 | 7.090792 | 4.173596 | 2.783401 | 1.583666 | 1.597459 | 2.261942 | 1.00393 | 1.544679 | 1.758872 | 0.800143 |
| **FOXP3** | 6.364239 | 5.122719 | 10.5595 | 4.798825 | 3.151155 | 4.451431 | 5.14595 | 6.126914 | 4.868602 | 4.448126 | 2.558763 | 4.883771 | 6.386526 | 7.160714 | 2.363597 | 4.173596 | 1.8556 | 6.334663 | 1.597459 | 5.42866 | 3.513756 | 1.029786 | 3.957461 | 2.400428 |
| **FYN** | 91.22076 | 82.98805 | 61.59708 | 105.5741 | 68.27502 | 95.26062 | 96.74387 | 80.87526 | 73.02903 | 65.98053 | 76.76288 | 72.4426 | 277.1752 | 170.0669 | 200.9058 | 245.7205 | 153.087 | 230.6873 | 200.8804 | 260.1233 | 221.3666 | 237.8806 | 262.9513 | 279.6499 |
| **GATA3** | 5.303533 | 12.29453 | 3.519833 | 2.399412 | 7.352695 | 2.670858 | 9.262711 | 6.126914 | 8.346175 | 2.965417 | 3.411684 | 4.069809 | 5.747874 | 3.580357 | 3.939329 | 5.738695 | 7.422402 | 5.278885 | 2.396188 | 4.523883 | 3.513756 | 2.059572 | 3.957461 | 2.8005 |
| **GBP1** | 4.242826 | 4.098175 | 3.519833 | 1.199706 | 4.20154 | 6.232003 | 3.08757 | 3.676148 | 2.086544 | 2.965417 | 4.264605 | 0.813962 | 84.30215 | 99.05654 | 59.08994 | 101.7314 | 72.36842 | 287.1714 | 102.2373 | 62.88198 | 115.954 | 66.42121 | 78.7095 | 86.41542 |
| **GBP5** | 3.18212 | 2.049088 | 1.759917 | 2.399412 | 2.10077 | 5.341717 | 2.05838 | 2.450765 | 0.695515 | 2.224063 | 1.705842 | 2.441885 | 6.386526 | 5.370535 | 7.090792 | 11.47739 | 9.278002 | 23.75498 | 8.786022 | 6.785825 | 7.027513 | 5.663824 | 3.078025 | 4.400786 |
| **GFI1** | 76.37087 | 92.20894 | 42.238 | 67.18355 | 74.57733 | 39.17259 | 54.54707 | 112.7352 | 38.2533 | 60.0497 | 38.38144 | 59.41921 | 3.831916 | 4.773809 | 7.878658 | 7.303793 | 12.0614 | 11.08566 | 5.591105 | 5.881048 | 6.023582 | 8.23829 | 4.836897 | 5.601 |
| **GNLY** | 10.60707 | 8.19635 | 7.039667 | 9.597649 | 10.50385 | 14.24458 | 5.14595 | 4.901531 | 6.955146 | 7.413543 | 6.823368 | 5.697732 | 12.1344 | 12.53125 | 6.302927 | 17.73778 | 18.556 | 6.334663 | 10.78285 | 28.50046 | 10.0393 | 22.1404 | 9.673793 | 22.00393 |
| **GP1BB** | 3.18212 | 8.19635 | 3.519833 | 2.399412 | 1.050385 | 2.670858 | 5.14595 | 7.352296 | 7.650661 | 3.706772 | 3.411684 | 3.255847 | 1.277305 | 5.370535 | 5.515061 | 3.651897 | 8.350202 | 4.223108 | 1.198094 | 6.333436 | 5.521617 | 1.544679 | 3.517743 | 4.000714 |
| **GPI** | 82.73511 | 63.52172 | 82.71608 | 70.78266 | 69.32541 | 84.57719 | 96.74387 | 98.03062 | 58.42323 | 71.17001 | 83.58625 | 63.48902 | 146.2515 | 137.247 | 123.6949 | 145.0325 | 100.2024 | 158.8945 | 164.1389 | 166.4789 | 140.5503 | 140.5658 | 153.4615 | 179.6321 |
| **GPR183** | 917.5111 | 666.978 | 392.4614 | 1793.561 | 598.7194 | 1716.472 | 1005.519 | 779.3434 | 864.5246 | 690.2009 | 980.8591 | 805.8221 | 182.6547 | 227.9494 | 226.1175 | 427.2719 | 300.6073 | 320.9562 | 252.3985 | 368.6965 | 200.2841 | 214.7104 | 183.8021 | 286.4511 |
| **GZMA** | 6.364239 | 3.073631 | 8.799583 | 2.399412 | 4.20154 | 4.451431 | 6.175141 | 2.450765 | 7.650661 | 7.413543 | 5.970447 | 2.441885 | 4.470568 | 4.177083 | 4.727195 | 4.173596 | 4.639001 | 6.862551 | 6.789199 | 7.690601 | 6.525547 | 5.663824 | 5.276615 | 12.40221 |
| **GZMB** | 6403.485 | 4343.041 | 5362.466 | 9853.187 | 6391.592 | 6043.262 | 8312.768 | 8888.926 | 9651.656 | 6347.476 | 8206.805 | 7416.82 | 5.747874 | 6.563988 | 10.24226 | 11.47739 | 8.350202 | 4.223108 | 8.786022 | 7.238213 | 5.521617 | 8.753183 | 3.078025 | 10.40186 |
| **GZMK** | 3.18212 | 4.098175 | 7.039667 | 3.599119 | 3.151155 | 3.561145 | 1.02919 | 3.676148 | 4.868602 | 2.965417 | 2.558763 | 0.813962 | 0.638653 | 1.790178 | 3.151463 | 2.608498 | 3.711201 | 2.639443 | 3.594282 | 2.71433 | 3.011791 | 3.604252 | 3.078025 | 4.000714 |
| **HAMP** | 7.424946 | 7.171807 | 3.519833 | 7.198237 | 6.30231 | 6.232003 | 2.05838 | 3.676148 | 5.564117 | 2.965417 | 2.558763 | 3.255847 | 7.663832 | 9.547618 | 6.302927 | 5.216995 | 7.422402 | 4.750997 | 2.795553 | 3.619107 | 3.011791 | 4.119145 | 5.276615 | 4.800857 |
| **HAVCR2** | 7.424946 | 7.171807 | 8.799583 | 5.998531 | 8.403079 | 12.46401 | 18.52542 | 6.126914 | 13.91029 | 8.896252 | 14.49966 | 17.90716 | 93.24328 | 81.15475 | 64.605 | 105.905 | 76.07962 | 111.3845 | 121.4069 | 91.38244 | 116.4559 | 107.0978 | 136.7523 | 148.8266 |
| **HFE** | 3.18212 | 2.049088 | 1.759917 | 1.199706 | 2.10077 | 2.670858 | 3.08757 | 1.225383 | 2.086544 | 2.224063 | 3.411684 | 0.813962 | 4.470568 | 2.386905 | 6.302927 | 4.173596 | 7.422402 | 7.918328 | 5.591105 | 8.14299 | 7.529478 | 3.089359 | 3.957461 | 3.600643 |
| **HLA-A** | 1071.314 | 1229.453 | 915.1567 | 1401.257 | 839.2576 | 1723.594 | 1025.073 | 1013.392 | 1151.772 | 664.2535 | 884.479 | 941.7538 | 1254.952 | 1358.149 | 1172.344 | 1278.686 | 616.0594 | 1521.903 | 1027.965 | 1082.565 | 1306.615 | 686.8674 | 967.3793 | 1110.198 |
| **HLA-B** | 2691.012 | 2247.849 | 1976.386 | 3391.569 | 1892.794 | 3378.636 | 2507.107 | 2184.857 | 1977.348 | 2089.136 | 1948.924 | 1669.436 | 2442.846 | 1974.567 | 1913.726 | 2363.821 | 1190.368 | 2229.801 | 1965.273 | 1795.077 | 1753.866 | 1746.003 | 1635.311 | 1638.292 |
| **HLA-C** | 566.4173 | 251.0132 | 278.0668 | 400.7019 | 277.3016 | 539.5134 | 593.8427 | 311.2472 | 324.1098 | 212.7687 | 125.3794 | 355.7013 | 554.3505 | 456.4955 | 374.2363 | 504.4834 | 185.56 | 740.0997 | 509.1899 | 408.959 | 604.868 | 317.1742 | 160.9367 | 506.8905 |
| **HLA-DMA** | 551.5674 | 506.1246 | 438.2193 | 815.8002 | 528.3436 | 571.5637 | 844.9651 | 546.5207 | 666.9985 | 659.064 | 620.9264 | 678.8441 | 942.0126 | 841.3839 | 854.8344 | 980.2734 | 575.2362 | 766.4942 | 1015.185 | 871.2999 | 1048.605 | 800.6588 | 945.3934 | 1028.184 |
| **HLA-DMB** | 792.3478 | 628.0453 | 491.0168 | 1002.954 | 668.0448 | 930.349 | 1275.167 | 861.4441 | 847.1368 | 1029.741 | 910.9196 | 1036.987 | 961.8109 | 750.0848 | 824.1077 | 993.8376 | 591.0088 | 962.8687 | 1140.985 | 1001.135 | 950.2201 | 896.4289 | 1100.614 | 1279.028 |
| **HLA-DOB** | 13.78918 | 18.44179 | 17.59917 | 11.99706 | 13.655 | 22.25715 | 28.81732 | 9.803062 | 13.91029 | 15.56844 | 13.64674 | 13.83735 | 124.5373 | 46.54464 | 50.42341 | 39.64916 | 38.96761 | 57.01196 | 72.68437 | 82.78706 | 81.31836 | 53.54888 | 83.5464 | 73.61314 |
| **HLA-DPA1** | 4050.838 | 3403.534 | 2560.679 | 4982.38 | 3111.24 | 4073.949 | 5395.014 | 3621.006 | 3344.034 | 3500.675 | 3417.654 | 3907.83 | 13803.84 | 10689.75 | 7219.215 | 10831.53 | 5951.839 | 9054.872 | 13057.63 | 9691.967 | 12091.84 | 10444.09 | 11455.97 | 12351 |
| **HLA-DPB1** | 3067.563 | 2973.226 | 2096.061 | 3770.676 | 2810.83 | 3164.967 | 4718.837 | 3199.474 | 2426.65 | 3147.79 | 3226.6 | 3274.568 | 12333.66 | 9988.002 | 7469.756 | 10131.93 | 5510.206 | 7858.677 | 13028.07 | 10369.65 | 10682.32 | 9777.82 | 12209.21 | 11717.29 |
| **HLA-DQA1** | 793.4085 | 529.6891 | 841.2402 | 694.6299 | 3.151155 | 902.7502 | 2.05838 | 1.225383 | 1544.042 | 1447.865 | 1.705842 | 5.697732 | 5056.852 | 2981.841 | 4025.207 | 2695.621 | 0.9278 | 3004.742 | 1.996823 | 3.166718 | 8021.404 | 4557.319 | 2.638307 | 2.8005 |
| **HLA-DQB1** | 360.6402 | 85.03714 | 302.7057 | 304.7254 | 2.10077 | 140.6652 | 1.02919 | 2.450765 | 801.2328 | 461.8637 | 1.705842 | 1.627924 | 1656.665 | 566.8898 | 947.8026 | 840.9796 | 0.9278 | 491.9921 | 2.396188 | 2.261942 | 2871.743 | 940.1948 | 1.758872 | 2.400428 |
| **HLA-DRA** | 6148.916 | 4931.129 | 5019.282 | 8126.81 | 5397.928 | 6893.486 | 8572.124 | 7398.861 | 6074.625 | 7688.586 | 7330.856 | 7794.498 | 24068.9 | 19435.37 | 16998.21 | 20052.56 | 12543.86 | 18054.84 | 23407.16 | 23056.42 | 19185.11 | 19674.07 | 22440.12 | 24338.34 |
| **HLA-DRB1** | 882.5078 | 343.2222 | 688.1274 | 225.5448 | 278.352 | 424.6665 | 368.4501 | 372.5163 | 1324.955 | 1013.431 | 218.3478 | 216.5138 | 4343.477 | 1761.536 | 3279.885 | 1055.398 | 922.2334 | 1582.61 | 1389.789 | 1299.712 | 6043.159 | 3478.103 | 815.6767 | 757.3352 |
| **HLA-DRB3** | 1777.744 | 1244.821 | 1483.61 | 2591.365 | 1850.778 | 2028.962 | 2641.931 | 2754.66 | 1804.86 | 2540.621 | 1623.961 | 1647.459 | 8451.929 | 5577.002 | 6978.128 | 7077.376 | 4667.763 | 5618.846 | 7154.618 | 9103.863 | 7338.731 | 7514.865 | 6099.766 | 5899.853 |
| **HRAS** | 27.57837 | 12.29453 | 14.07933 | 21.59471 | 15.75577 | 15.13486 | 10.2919 | 15.92998 | 22.25647 | 21.49928 | 10.23505 | 17.0932 | 13.41171 | 22.07887 | 11.03012 | 13.56419 | 12.9892 | 12.66933 | 11.98094 | 13.57165 | 16.06289 | 13.38722 | 13.19154 | 17.20307 |
| **ICAM1** | 73.18875 | 204.9088 | 110.8748 | 148.7636 | 177.5151 | 102.3829 | 76.16007 | 241.4004 | 80.67969 | 99.34148 | 68.23368 | 75.69844 | 68.97448 | 194.5327 | 113.4527 | 179.9863 | 131.7476 | 103.994 | 99.04243 | 171.4552 | 81.82032 | 92.16587 | 126.199 | 78.01393 |
| **ICAM2** | 31.8212 | 17.41724 | 22.87892 | 41.98972 | 25.20924 | 33.83087 | 41.1676 | 28.1838 | 31.29816 | 39.29178 | 36.6756 | 50.46563 | 95.7979 | 90.70237 | 70.12006 | 82.95022 | 55.66801 | 117.1913 | 122.6049 | 97.26349 | 115.954 | 98.34458 | 123.5607 | 140.025 |
| **ICAM3** | 164.4095 | 246.9151 | 188.3111 | 209.9486 | 127.0966 | 154.0195 | 188.3418 | 208.3151 | 170.4011 | 160.8739 | 220.0536 | 202.6765 | 638.014 | 511.991 | 507.3856 | 568.1308 | 302.4629 | 455.0399 | 650.9644 | 586.7477 | 630.4683 | 623.0207 | 764.2297 | 761.736 |
| **ICAM4** | 5.303533 | 9.220894 | 8.799583 | 10.79736 | 6.30231 | 1.780572 | 5.14595 | 3.676148 | 3.477573 | 8.154897 | 4.264605 | 10.5815 | 5.109221 | 4.773809 | 9.45439 | 8.868892 | 3.711201 | 5.278885 | 9.584751 | 10.40493 | 8.031443 | 6.178717 | 7.914922 | 9.201642 |
| **ICAM5** | 1.060707 | 2.049088 | 3.519833 | 1.199706 | 4.20154 | 1.780572 | 1.02919 | 2.450765 | 2.782058 | 1.482709 | 2.558763 | 4.069809 | 1.277305 | 0.596726 | 0.787866 | 2.086798 | 0.9278 | 1.583666 | 0.798729 | 0.904777 | 0.501965 | 1.544679 | 0.879436 | 2.400428 |
| **ICOS** | 7.424946 | 3.073631 | 10.5595 | 4.798825 | 6.30231 | 2.670858 | 1.02919 | 8.577679 | 4.173088 | 2.965417 | 4.264605 | 4.069809 | 3.193263 | 2.983631 | 2.363597 | 3.651897 | 3.711201 | 5.278885 | 1.996823 | 4.071495 | 4.015721 | 3.604252 | 1.758872 | 2.000357 |
| **ICOSLG** | 30.76049 | 34.83449 | 24.63883 | 68.38325 | 30.46116 | 30.26973 | 33.96327 | 19.60612 | 37.55779 | 53.37751 | 47.76357 | 36.62828 | 11.49575 | 41.77083 | 38.60543 | 51.12655 | 15.7726 | 17.42032 | 9.984116 | 18.09553 | 6.023582 | 11.84254 | 17.149 | 11.202 |
| **IDO1** | 3.18212 | 5.122719 | 3.519833 | 1.199706 | 3.151155 | 2.670858 | 3.08757 | 1.225383 | 0.695515 | 3.706772 | 2.558763 | 2.441885 | 30.65533 | 12.53125 | 11.03012 | 20.86798 | 10.2058 | 49.09364 | 29.95235 | 7.238213 | 29.11398 | 14.9319 | 46.17038 | 33.20593 |
| **IDO2** | 6.364239 | 4.098175 | 3.519833 | 1.199706 | 3.151155 | 2.670858 | 2.05838 | 3.676148 | 2.782058 | 2.224063 | 1.705842 | 0.813962 | 4.470568 | 4.177083 | 3.939329 | 3.130197 | 3.711201 | 2.111554 | 1.597459 | 4.523883 | 1.505896 | 1.544679 | 1.758872 | 1.200214 |
| **IFI16** | 126.2241 | 278.6759 | 131.9938 | 219.5462 | 179.6158 | 239.487 | 305.6695 | 148.2713 | 236.475 | 209.8033 | 205.5539 | 231.9791 | 249.7132 | 255.9955 | 248.9656 | 257.7196 | 223.5999 | 383.775 | 357.032 | 198.5985 | 298.1673 | 268.2593 | 292.8521 | 317.6567 |
| **IFI35** | 48.7925 | 58.399 | 54.55742 | 79.18061 | 38.86424 | 63.21032 | 59.69302 | 41.66301 | 52.85911 | 63.01512 | 63.11615 | 43.95394 | 37.04185 | 51.91517 | 38.60543 | 34.95387 | 35.25641 | 60.17929 | 33.14727 | 28.95285 | 40.65918 | 24.71487 | 35.17743 | 38.40686 |
| **IFI44L** | 35.00331 | 1006.102 | 45.75783 | 77.9809 | 27.31001 | 24.92801 | 36.02165 | 18.38074 | 68.85595 | 80.80762 | 26.44055 | 29.30262 | 28.10072 | 429.0461 | 23.63597 | 43.30106 | 13.917 | 32.2012 | 20.76696 | 14.02404 | 35.63953 | 11.84254 | 14.07097 | 15.60279 |
| **IFI6** | 5.303533 | 8.19635 | 8.799583 | 1.199706 | 5.251925 | 0.890286 | 1.02919 | 3.676148 | 5.564117 | 8.154897 | 7.676288 | 3.255847 | 35.76455 | 212.4345 | 28.36317 | 44.34446 | 36.18421 | 72.84862 | 35.14409 | 41.61973 | 48.69062 | 31.92337 | 29.02138 | 41.60743 |
| **IFIH1** | 18.03201 | 36.88358 | 7.039667 | 19.1953 | 13.655 | 16.02515 | 15.43785 | 22.05689 | 11.12823 | 26.68876 | 7.676288 | 12.20943 | 25.54611 | 44.15773 | 30.72677 | 40.17086 | 29.68961 | 33.25698 | 43.53075 | 37.54823 | 30.11791 | 32.95316 | 36.49658 | 43.20771 |
| **IFIT2** | 68.94592 | 200.8106 | 72.15658 | 121.1703 | 57.77117 | 66.77146 | 168.7872 | 68.62143 | 138.4074 | 155.6844 | 51.17526 | 263.7236 | 21.71419 | 92.49255 | 11.81799 | 18.78118 | 12.9892 | 24.81076 | 22.36442 | 17.64314 | 26.10219 | 19.56594 | 11.87238 | 30.8055 |
| **IFITM1** | 9.546359 | 64.54626 | 14.07933 | 13.19677 | 21.0077 | 25.8183 | 17.49623 | 8.577679 | 11.82375 | 14.82709 | 8.529209 | 7.325656 | 139.2263 | 1134.376 | 155.2096 | 320.3235 | 379.4703 | 139.8905 | 103.4354 | 241.123 | 140.5503 | 112.2467 | 127.5182 | 125.2224 |
| **IFNA1/13** | 2.121413 | 3.073631 | 7.039667 | 1.199706 | 7.352695 | 3.561145 | 1.02919 | 1.225383 | 2.086544 | 2.224063 | 5.117526 | 4.069809 | 1.277305 | 4.177083 | 2.363597 | 3.130197 | 5.566801 | 2.111554 | 3.194917 | 1.357165 | 2.509826 | 2.574466 | 1.319154 | 2.000357 |
| **IFNA2** | 2.121413 | 3.073631 | 10.5595 | 2.399412 | 4.20154 | 2.670858 | 2.05838 | 2.450765 | 3.477573 | 2.965417 | 1.705842 | 1.627924 | 6.386526 | 2.983631 | 2.363597 | 1.565099 | 6.494602 | 1.583666 | 3.194917 | 5.42866 | 3.513756 | 4.634038 | 2.638307 | 1.600286 |
| **IFNAR1** | 26.51766 | 16.3927 | 24.63883 | 34.79148 | 13.655 | 30.26973 | 41.1676 | 34.31072 | 32.68919 | 56.34293 | 63.11615 | 42.32601 | 11.49575 | 12.53125 | 13.39372 | 14.60759 | 9.278002 | 21.11554 | 14.37713 | 19.90509 | 20.07861 | 14.41701 | 18.02843 | 12.40221 |
| **IFNAR2** | 560.053 | 614.7263 | 443.499 | 736.6196 | 488.429 | 645.4575 | 772.9218 | 622.4944 | 566.1489 | 772.4912 | 738.6295 | 826.9851 | 167.327 | 217.805 | 229.269 | 182.5948 | 182.7766 | 202.7092 | 241.6156 | 177.3362 | 202.7939 | 179.1828 | 196.9936 | 234.8419 |
| **IFNB1** | 3.18212 | 1.024544 | 1.759917 | 1.199706 | 1.050385 | 3.561145 | 2.05838 | 4.901531 | 2.782058 | 4.448126 | 5.117526 | 0.813962 | 1.277305 | 4.773809 | 2.363597 | 2.608498 | 1.8556 | 2.639443 | 2.795553 | 3.619107 | 1.00393 | 3.089359 | 1.758872 | 2.400428 |
| **IFNG** | 1.060707 | 4.098175 | 5.27975 | 2.399412 | 5.251925 | 0.890286 | 1.02919 | 1.225383 | 2.782058 | 2.224063 | 2.558763 | 2.441885 | 2.554611 | 0.596726 | 2.363597 | 0.5217 | 4.639001 | 2.639443 | 1.996823 | 2.261942 | 2.509826 | 2.059572 | 0.439718 | 2.000357 |
| **IFNGR1** | 156.9846 | 171.0988 | 135.5136 | 207.5492 | 189.0693 | 228.8035 | 356.0998 | 192.3851 | 334.5425 | 283.9387 | 330.0804 | 269.4213 | 301.444 | 335.3601 | 293.0861 | 272.8489 | 273.7011 | 400.1395 | 502.0014 | 318.9338 | 546.1381 | 423.2421 | 413.7745 | 468.8837 |
| **IGF2R** | 175.0166 | 188.5161 | 107.3549 | 220.7459 | 140.7516 | 181.6184 | 221.2759 | 207.0897 | 157.8818 | 155.6844 | 176.5546 | 175.0018 | 23.63015 | 14.32143 | 19.69665 | 10.95569 | 17.6282 | 17.94821 | 25.9587 | 18.09553 | 15.56092 | 13.38722 | 20.22702 | 20.80371 |
| **IKBKAP** | 28.63908 | 22.53996 | 35.19833 | 38.3906 | 28.36039 | 50.74631 | 47.34274 | 42.8884 | 27.12507 | 43.7399 | 37.52852 | 44.7679 | 26.18476 | 22.67559 | 27.5753 | 29.73687 | 19.48381 | 30.08965 | 35.14409 | 25.33375 | 29.11398 | 25.22976 | 25.50364 | 37.20664 |
| **IKBKB** | 59.39956 | 49.1781 | 45.75783 | 97.1762 | 49.36809 | 64.99089 | 131.7363 | 77.19911 | 88.33035 | 142.34 | 78.46873 | 97.67541 | 61.31065 | 39.98065 | 57.51421 | 60.51715 | 34.32861 | 45.39842 | 65.4958 | 39.81017 | 52.70634 | 58.18292 | 71.2343 | 73.61314 |
| **IKBKE** | 6.364239 | 8.19635 | 7.039667 | 10.79736 | 7.352695 | 8.012575 | 8.233521 | 2.450765 | 11.82375 | 14.82709 | 6.823368 | 4.883771 | 9.57979 | 7.75744 | 14.18158 | 10.95569 | 13.917 | 13.19721 | 9.185387 | 9.047766 | 9.537338 | 10.29786 | 15.82984 | 11.202 |
| **IKBKG** | 39.24614 | 45.07993 | 42.238 | 55.18648 | 36.76347 | 49.85603 | 73.0725 | 42.8884 | 65.37837 | 73.39408 | 57.1457 | 54.53544 | 45.98299 | 29.83631 | 44.90835 | 52.69165 | 47.31781 | 41.17531 | 52.71613 | 33.47674 | 36.14149 | 37.0723 | 54.96473 | 44.40793 |
| **IKZF1** | 107.1314 | 94.25803 | 63.357 | 122.37 | 82.98041 | 110.3955 | 189.371 | 89.45294 | 130.0612 | 128.9957 | 168.8783 | 146.5131 | 33.84859 | 48.33482 | 24.42384 | 47.99636 | 39.89541 | 49.62152 | 71.48627 | 52.92943 | 68.26726 | 74.6595 | 80.46837 | 75.6135 |
| **IKZF2** | 22.27484 | 22.53996 | 15.83925 | 15.59618 | 23.10847 | 27.59887 | 14.40866 | 28.1838 | 25.73404 | 17.05115 | 21.32302 | 27.6747 | 5.747874 | 5.370535 | 4.727195 | 4.173596 | 7.422402 | 3.167331 | 5.99047 | 5.42866 | 8.031443 | 3.089359 | 5.276615 | 4.000714 |
| **IKZF3** | 3.18212 | 3.073631 | 1.759917 | 1.199706 | 1.050385 | 3.561145 | 1.02919 | 2.450765 | 2.086544 | 1.482709 | 4.264605 | 0.813962 | 2.554611 | 2.386905 | 6.302927 | 0.5217 | 2.783401 | 2.111554 | 2.396188 | 2.71433 | 0.501965 | 2.059572 | 1.319154 | 1.600286 |
| **IL10** | 1.060707 | 4.098175 | 8.799583 | 5.998531 | 4.20154 | 1.780572 | 9.262711 | 4.901531 | 1.391029 | 6.672189 | 4.264605 | 4.883771 | 1.915958 | 1.790178 | 3.939329 | 4.695296 | 2.783401 | 4.223108 | 3.594282 | 5.881048 | 1.505896 | 3.089359 | 4.397179 | 2.400428 |
| **IL10RA** | 325.6369 | 397.523 | 286.8664 | 505.0763 | 349.7782 | 373.0299 | 433.289 | 438.687 | 284.4655 | 312.1102 | 409.4021 | 390.7016 | 339.7632 | 464.2529 | 432.5383 | 547.2628 | 237.5169 | 380.6076 | 484.0299 | 366.4345 | 378.4817 | 344.9784 | 548.7679 | 548.0978 |
| **IL11RA** | 3.18212 | 16.3927 | 10.5595 | 7.198237 | 3.151155 | 4.451431 | 4.11676 | 1.225383 | 8.346175 | 9.637606 | 6.823368 | 5.697732 | 6.386526 | 5.370535 | 8.666524 | 4.695296 | 3.711201 | 6.862551 | 8.786022 | 4.071495 | 2.509826 | 3.604252 | 3.078025 | 4.000714 |
| **IL12A** | 9.546359 | 5.122719 | 10.5595 | 3.599119 | 7.352695 | 4.451431 | 1.02919 | 3.676148 | 3.477573 | 4.448126 | 5.117526 | 5.697732 | 2.554611 | 5.370535 | 7.878658 | 2.086798 | 5.566801 | 4.223108 | 3.594282 | 3.166718 | 5.521617 | 4.119145 | 4.397179 | 2.8005 |
| **IL12B** | 7.424946 | 6.147263 | 5.27975 | 2.399412 | 2.10077 | 3.561145 | 3.08757 | 4.901531 | 6.955146 | 2.965417 | 5.117526 | 4.069809 | 5.747874 | 5.967261 | 3.151463 | 6.782094 | 9.278002 | 6.334663 | 3.993646 | 4.976272 | 4.517687 | 4.119145 | 4.836897 | 2.8005 |
| **IL12RB1** | 9.546359 | 18.44179 | 14.07933 | 10.79736 | 14.70539 | 18.69601 | 11.32109 | 17.15536 | 11.12823 | 11.12031 | 11.08797 | 14.65131 | 26.18476 | 22.67559 | 19.69665 | 23.99818 | 19.48381 | 31.67331 | 27.55616 | 23.97658 | 24.59629 | 18.02126 | 29.02138 | 21.60386 |
| **IL13** | 8.485652 | 6.147263 | 7.039667 | 2.399412 | 6.30231 | 0.890286 | 4.11676 | 2.450765 | 7.650661 | 5.18948 | 2.558763 | 4.883771 | 5.109221 | 2.386905 | 7.878658 | 4.695296 | 7.422402 | 1.583666 | 4.792376 | 4.976272 | 5.521617 | 3.604252 | 5.716332 | 2.400428 |
| **IL13RA1** | 100.7671 | 137.2889 | 65.11692 | 87.57855 | 96.63541 | 81.90633 | 118.3569 | 113.9606 | 92.50344 | 111.9445 | 84.43917 | 164.4203 | 680.8037 | 584.7916 | 602.7174 | 509.7004 | 310.8131 | 487.2411 | 716.4602 | 527.9372 | 753.4497 | 716.2163 | 697.3926 | 686.9226 |
| **IL15** | 15.9106 | 11.26998 | 7.039667 | 8.397943 | 10.50385 | 15.13486 | 8.233521 | 13.47921 | 10.43272 | 14.82709 | 8.529209 | 8.953579 | 8.941137 | 8.950892 | 11.03012 | 13.04249 | 14.8448 | 11.61355 | 14.37713 | 10.40493 | 15.05896 | 14.41701 | 12.3121 | 6.801214 |
| **IL16** | 151.681 | 161.8779 | 214.7098 | 231.5433 | 163.86 | 227.9133 | 278.9105 | 159.2998 | 237.1705 | 246.1296 | 256.7292 | 238.4908 | 177.5454 | 141.4241 | 143.3916 | 169.5523 | 94.63562 | 172.0917 | 253.1972 | 133.9069 | 212.3313 | 206.987 | 237.4477 | 229.641 |
| **IL17A** | 1.060707 | 4.098175 | 1.759917 | 2.399412 | 3.151155 | 0.890286 | 2.05838 | 2.450765 | 2.782058 | 4.448126 | 0.852921 | 2.441885 | 4.470568 | 1.790178 | 1.575732 | 5.738695 | 4.639001 | 0.527889 | 1.597459 | 2.261942 | 0.501965 | 3.089359 | 2.638307 | 1.200214 |
| **IL17B** | 2.121413 | 4.098175 | 10.5595 | 7.198237 | 7.352695 | 3.561145 | 4.11676 | 4.901531 | 4.173088 | 2.224063 | 3.411684 | 4.069809 | 6.386526 | 4.773809 | 4.727195 | 2.608498 | 4.639001 | 4.223108 | 5.591105 | 3.619107 | 3.513756 | 3.604252 | 5.716332 | 4.000714 |
| **IL17F** | 6.364239 | 8.19635 | 10.5595 | 3.599119 | 6.30231 | 8.012575 | 4.11676 | 6.126914 | 2.782058 | 3.706772 | 3.411684 | 1.627924 | 4.470568 | 2.386905 | 7.878658 | 1.043399 | 4.639001 | 3.167331 | 3.993646 | 4.523883 | 3.513756 | 4.634038 | 3.078025 | 3.600643 |
| **IL18** | 35.00331 | 32.7854 | 36.95825 | 26.39354 | 35.71309 | 27.59887 | 30.8757 | 22.05689 | 31.29816 | 54.11887 | 36.6756 | 45.58186 | 171.1589 | 134.8601 | 122.1192 | 102.7748 | 70.51282 | 97.65938 | 150.9598 | 121.6925 | 156.1112 | 144.1701 | 130.5962 | 157.2281 |
| **IL18R1** | 204.7164 | 169.0497 | 128.4739 | 251.9383 | 141.802 | 184.2892 | 293.3192 | 189.9343 | 221.8692 | 214.2514 | 216.6419 | 297.91 | 60.03335 | 32.81994 | 19.69665 | 38.08407 | 38.03981 | 34.84064 | 69.09008 | 44.78644 | 62.24368 | 40.67656 | 80.46837 | 67.212 |
| **IL18RAP** | 6.364239 | 10.24544 | 3.519833 | 5.998531 | 7.352695 | 4.451431 | 16.46704 | 8.577679 | 7.650661 | 13.34438 | 6.823368 | 14.65131 | 7.663832 | 5.370535 | 3.939329 | 3.130197 | 3.711201 | 3.167331 | 6.789199 | 4.071495 | 3.513756 | 4.634038 | 5.276615 | 6.401143 |
| **IL19** | 3.18212 | 3.073631 | 1.759917 | 4.798825 | 3.151155 | 0.890286 | 1.02919 | 1.225383 | 1.391029 | 0.741354 | 0.852921 | 2.441885 | 1.277305 | 0.596726 | 1.575732 | 1.043399 | 2.783401 | 0.527889 | 0.798729 | 2.261942 | 1.00393 | 3.089359 | 1.758872 | 0.400071 |
| **IL1A** | 3.18212 | 1.024544 | 1.759917 | 1.199706 | 4.20154 | 0.890286 | 1.02919 | 2.450765 | 1.391029 | 1.482709 | 3.411684 | 0.813962 | 5.747874 | 0.596726 | 2.363597 | 3.130197 | 7.422402 | 1.583666 | 3.594282 | 2.71433 | 4.015721 | 1.544679 | 3.517743 | 1.600286 |
| **IL1B** | 7.424946 | 5.122719 | 3.519833 | 1.199706 | 4.20154 | 3.561145 | 2.05838 | 6.126914 | 5.564117 | 5.930835 | 4.264605 | 4.883771 | 15.96632 | 35.80357 | 51.99914 | 86.08042 | 75.15182 | 40.64742 | 78.27547 | 47.95316 | 58.22796 | 112.7616 | 88.38329 | 160.8287 |
| **IL1R1** | 6.364239 | 6.147263 | 8.799583 | 2.399412 | 5.251925 | 0.890286 | 3.08757 | 4.901531 | 4.868602 | 4.448126 | 0.852921 | 1.627924 | 16.60497 | 6.563988 | 12.60585 | 10.95569 | 14.8448 | 5.278885 | 7.987293 | 13.57165 | 12.04716 | 7.208504 | 6.595768 | 10.40186 |
| **IL1R2** | 9.546359 | 8.19635 | 5.27975 | 9.597649 | 6.30231 | 9.793148 | 8.233521 | 7.352296 | 12.51926 | 5.18948 | 6.823368 | 7.325656 | 36.4032 | 14.91815 | 34.6661 | 41.73596 | 9.278002 | 13.7251 | 27.95553 | 27.59569 | 27.10612 | 25.22976 | 25.94335 | 34.80621 |
| **IL1RAP** | 7.424946 | 7.171807 | 12.31942 | 4.798825 | 6.30231 | 6.232003 | 9.262711 | 8.577679 | 6.259631 | 8.154897 | 9.38213 | 6.511694 | 23.63015 | 14.91815 | 24.42384 | 18.78118 | 19.48381 | 11.61355 | 20.3676 | 19.4527 | 15.56092 | 20.59572 | 19.34759 | 25.2045 |
| **IL1RL1** | 3.18212 | 2.049088 | 5.27975 | 5.998531 | 5.251925 | 1.780572 | 2.05838 | 1.225383 | 4.173088 | 1.482709 | 1.705842 | 4.069809 | 3.831916 | 4.773809 | 3.939329 | 2.086798 | 0.9278 | 4.750997 | 3.194917 | 4.071495 | 5.019652 | 4.119145 | 3.957461 | 2.400428 |
| **IL1RL2** | 3.18212 | 3.073631 | 12.31942 | 8.397943 | 7.352695 | 6.232003 | 7.204331 | 4.901531 | 6.955146 | 5.930835 | 4.264605 | 8.139618 | 6.386526 | 5.370535 | 5.515061 | 3.651897 | 5.566801 | 3.69522 | 4.792376 | 4.976272 | 2.007861 | 3.089359 | 4.836897 | 3.200571 |
| **IL1RN** | 4.242826 | 3.073631 | 12.31942 | 2.399412 | 4.20154 | 7.122289 | 2.05838 | 4.901531 | 2.782058 | 1.482709 | 4.264605 | 3.255847 | 57.47874 | 53.70535 | 35.45396 | 102.2531 | 45.46221 | 49.09364 | 54.71296 | 75.09646 | 43.67097 | 63.33185 | 63.75909 | 61.21093 |
| **IL2** | 2.121413 | 7.171807 | 1.759917 | 1.199706 | 2.10077 | 3.561145 | 5.14595 | 7.352296 | 2.086544 | 3.706772 | 4.264605 | 0.813962 | 2.554611 | 2.983631 | 3.151463 | 2.086798 | 1.8556 | 1.583666 | 1.996823 | 3.166718 | 1.505896 | 0.514893 | 3.517743 | 1.600286 |
| **IL20** | 8.485652 | 11.26998 | 7.039667 | 5.998531 | 5.251925 | 10.68343 | 2.05838 | 2.450765 | 6.955146 | 7.413543 | 5.117526 | 4.069809 | 4.470568 | 4.177083 | 7.090792 | 5.738695 | 6.494602 | 6.334663 | 3.993646 | 6.333436 | 4.517687 | 4.634038 | 5.716332 | 2.8005 |
| **IL21** | 1.060707 | 1.024544 | 1.759917 | 2.399412 | 1.050385 | 0.890286 | 3.08757 | 1.225383 | 0.695515 | 4.448126 | 3.411684 | 1.627924 | 0.638653 | 1.790178 | 1.575732 | 1.043399 | 4.639001 | 4.223108 | 1.996823 | 1.809553 | 4.517687 | 2.574466 | 0.879436 | 2.000357 |
| **IL21R** | 6.364239 | 2.049088 | 8.799583 | 3.599119 | 7.352695 | 16.02515 | 28.81732 | 6.126914 | 7.650661 | 12.60302 | 12.79381 | 4.883771 | 10.85709 | 10.14434 | 7.878658 | 7.825493 | 25.05061 | 14.78088 | 11.98094 | 14.02404 | 8.031443 | 11.84254 | 7.035486 | 10.40186 |
| **IL22** | 2.121413 | 5.122719 | 3.519833 | 4.798825 | 1.050385 | 0.890286 | 3.08757 | 4.901531 | 1.391029 | 2.224063 | 4.264605 | 3.255847 | 3.193263 | 4.773809 | 1.575732 | 2.608498 | 6.494602 | 2.639443 | 2.795553 | 2.261942 | 1.505896 | 1.544679 | 1.319154 | 2.000357 |
| **IL22RA2** | 3.18212 | 1.024544 | 1.759917 | 1.199706 | 2.10077 | 3.561145 | 1.02919 | 2.450765 | 1.391029 | 3.706772 | 3.411684 | 2.441885 | 2.554611 | 2.386905 | 0.787866 | 4.695296 | 2.783401 | 3.167331 | 1.198094 | 1.357165 | 3.513756 | 4.119145 | 1.758872 | 2.400428 |
| **IL23A** | 5.303533 | 8.19635 | 12.31942 | 2.399412 | 7.352695 | 1.780572 | 6.175141 | 1.225383 | 4.868602 | 0.741354 | 0.852921 | 1.627924 | 3.831916 | 4.177083 | 3.939329 | 1.565099 | 9.278002 | 4.223108 | 1.597459 | 3.619107 | 6.023582 | 3.604252 | 3.517743 | 6.001071 |
| **IL23R** | 2.121413 | 2.049088 | 1.759917 | 3.599119 | 7.352695 | 4.451431 | 2.05838 | 4.901531 | 2.782058 | 0.741354 | 5.117526 | 3.255847 | 1.915958 | 1.790178 | 1.575732 | 1.565099 | 3.711201 | 2.111554 | 3.194917 | 1.357165 | 3.011791 | 4.119145 | 1.758872 | 3.200571 |
| **IL26** | 10.60707 | 12.29453 | 10.5595 | 14.39647 | 15.75577 | 11.57372 | 8.233521 | 7.352296 | 9.04169 | 8.154897 | 10.23505 | 9.767541 | 9.57979 | 10.74107 | 18.12091 | 8.347192 | 15.7726 | 11.61355 | 5.19174 | 8.14299 | 7.529478 | 14.41701 | 7.914922 | 6.401143 |
| **IL27** | 2.121413 | 4.098175 | 7.039667 | 2.399412 | 1.050385 | 1.780572 | 3.08757 | 1.225383 | 2.086544 | 1.482709 | 6.823368 | 3.255847 | 1.277305 | 5.370535 | 2.363597 | 2.608498 | 2.783401 | 2.639443 | 3.194917 | 2.261942 | 5.521617 | 2.059572 | 2.198589 | 1.200214 |
| **IL28A** | 8.485652 | 7.171807 | 3.519833 | 8.397943 | 6.30231 | 8.012575 | 8.233521 | 11.02844 | 5.564117 | 4.448126 | 6.823368 | 8.139618 | 5.747874 | 5.967261 | 7.090792 | 2.608498 | 5.566801 | 5.806774 | 5.19174 | 4.523883 | 5.521617 | 5.148931 | 4.836897 | 3.600643 |
| **IL28A/B** | 6.364239 | 7.171807 | 3.519833 | 3.599119 | 3.151155 | 3.561145 | 6.175141 | 1.225383 | 4.868602 | 2.965417 | 4.264605 | 6.511694 | 1.915958 | 4.773809 | 3.151463 | 2.086798 | 6.494602 | 1.583666 | 1.198094 | 4.523883 | 4.517687 | 1.544679 | 1.758872 | 3.600643 |
| **IL28RA** | 96.52429 | 49.1781 | 54.55742 | 58.7856 | 59.87194 | 93.48005 | 113.2109 | 68.62143 | 118.933 | 106.755 | 71.64536 | 122.9082 | 8.302484 | 2.983631 | 6.302927 | 4.695296 | 5.566801 | 3.69522 | 5.19174 | 5.42866 | 6.023582 | 8.23829 | 3.517743 | 5.601 |
| **IL29** | 1.060707 | 2.049088 | 1.759917 | 2.399412 | 1.050385 | 2.670858 | 4.11676 | 1.225383 | 4.173088 | 3.706772 | 3.411684 | 0.813962 | 2.554611 | 2.386905 | 3.939329 | 1.043399 | 2.783401 | 4.223108 | 1.597459 | 2.71433 | 2.007861 | 3.604252 | 4.397179 | 0.800143 |
| **IL2RA** | 4.242826 | 4.098175 | 8.799583 | 1.199706 | 8.403079 | 6.232003 | 2.05838 | 7.352296 | 3.477573 | 3.706772 | 3.411684 | 6.511694 | 3.193263 | 4.177083 | 2.363597 | 3.651897 | 4.639001 | 7.39044 | 4.393011 | 3.619107 | 2.509826 | 2.059572 | 3.517743 | 5.200928 |
| **IL2RB** | 10.60707 | 10.24544 | 3.519833 | 8.397943 | 14.70539 | 13.35429 | 15.43785 | 4.901531 | 9.04169 | 10.37896 | 7.676288 | 6.511694 | 5.747874 | 13.12798 | 11.03012 | 14.08589 | 13.917 | 8.974105 | 9.984116 | 9.047766 | 10.54127 | 7.723397 | 7.035486 | 16.00286 |
| **IL2RG** | 816.744 | 711.0334 | 716.2861 | 854.1908 | 584.014 | 975.7536 | 913.9208 | 936.1924 | 752.5468 | 829.5755 | 782.1285 | 786.2871 | 611.8292 | 437.997 | 390.7815 | 374.5803 | 333.0803 | 520.4981 | 526.762 | 520.2466 | 504.977 | 595.7313 | 496.8812 | 542.0968 |
| **IL3** | 3.18212 | 9.220894 | 7.039667 | 3.599119 | 9.453464 | 0.890286 | 3.08757 | 6.126914 | 4.173088 | 3.706772 | 4.264605 | 1.627924 | 3.193263 | 2.386905 | 2.363597 | 1.565099 | 6.494602 | 7.39044 | 3.594282 | 4.071495 | 1.505896 | 4.634038 | 2.638307 | 3.200571 |
| **IL32** | 3000.739 | 4462.913 | 6027.715 | 7939.655 | 6285.503 | 4265.361 | 9747.459 | 5651.465 | 5681.659 | 5668.395 | 7499.734 | 3925.738 | 3153.028 | 1302.056 | 2369.9 | 1849.425 | 1605.094 | 1732.002 | 3434.935 | 1784.22 | 1904.456 | 2392.708 | 1318.714 | 2602.865 |
| **IL4** | 1.060707 | 3.073631 | 1.759917 | 3.599119 | 2.10077 | 2.670858 | 1.02919 | 1.225383 | 0.695515 | 4.448126 | 0.852921 | 2.441885 | 1.915958 | 2.983631 | 0.787866 | 1.565099 | 3.711201 | 0.527889 | 0.798729 | 1.809553 | 3.011791 | 5.148931 | 2.198589 | 0.400071 |
| **IL4R** | 84.85652 | 36.88358 | 96.79542 | 82.77973 | 69.32541 | 84.57719 | 133.7947 | 83.32603 | 84.15727 | 101.5655 | 111.7326 | 118.8384 | 56.84008 | 51.31845 | 70.90792 | 55.82185 | 49.17341 | 43.81475 | 63.09961 | 60.16765 | 46.68276 | 64.87653 | 70.79458 | 57.21021 |
| **IL5** | 2.121413 | 6.147263 | 1.759917 | 2.399412 | 3.151155 | 0.890286 | 2.05838 | 1.225383 | 1.391029 | 1.482709 | 3.411684 | 0.813962 | 1.277305 | 1.790178 | 2.363597 | 1.565099 | 2.783401 | 2.111554 | 1.996823 | 1.357165 | 2.509826 | 1.544679 | 3.078025 | 2.400428 |
| **IL6** | 5.303533 | 4.098175 | 1.759917 | 2.399412 | 1.050385 | 5.341717 | 2.05838 | 1.225383 | 1.391029 | 2.224063 | 0.852921 | 3.255847 | 3.193263 | 2.983631 | 7.878658 | 2.086798 | 1.8556 | 2.639443 | 1.996823 | 2.71433 | 3.011791 | 2.059572 | 0.879436 | 1.600286 |
| **IL6R** | 50.91391 | 30.73631 | 51.03758 | 44.38913 | 38.86424 | 64.1006 | 75.13088 | 45.33916 | 75.11558 | 63.01512 | 86.99794 | 98.48937 | 109.8483 | 83.54166 | 88.24097 | 94.42761 | 67.72942 | 97.13149 | 141.3751 | 89.57289 | 158.119 | 132.3275 | 157.8587 | 165.6296 |
| **IL6ST** | 44.54967 | 91.1844 | 45.75783 | 104.3744 | 72.47656 | 47.18517 | 135.8531 | 191.1597 | 165.5325 | 172.7356 | 210.6715 | 153.0248 | 53.00817 | 44.15773 | 51.99914 | 74.60303 | 54.74021 | 51.73308 | 63.49898 | 46.14361 | 64.25154 | 75.17439 | 69.47542 | 51.20914 |
| **IL7** | 2.121413 | 6.147263 | 1.759917 | 1.199706 | 7.352695 | 0.890286 | 3.08757 | 3.676148 | 3.477573 | 2.224063 | 2.558763 | 3.255847 | 10.21844 | 3.580357 | 4.727195 | 3.651897 | 8.350202 | 5.806774 | 6.789199 | 4.523883 | 5.019652 | 6.69361 | 5.716332 | 6.401143 |
| **IL7R** | 5.303533 | 3.073631 | 3.519833 | 1.199706 | 3.151155 | 4.451431 | 3.08757 | 6.126914 | 6.955146 | 2.965417 | 3.411684 | 3.255847 | 4.470568 | 3.580357 | 6.302927 | 3.651897 | 3.711201 | 6.334663 | 7.587928 | 5.881048 | 6.525547 | 5.663824 | 3.517743 | 5.601 |
| **IL8** | 5.303533 | 4.098175 | 5.27975 | 4.798825 | 4.20154 | 7.122289 | 2.05838 | 1.225383 | 4.868602 | 2.965417 | 3.411684 | 3.255847 | 45.34434 | 39.38393 | 45.69622 | 62.60394 | 190.1991 | 48.03786 | 161.3433 | 128.9307 | 44.17294 | 209.0466 | 148.1849 | 76.81371 |
| **IL9** | 1.060707 | 2.049088 | 1.759917 | 2.399412 | 4.20154 | 2.670858 | 2.05838 | 2.450765 | 4.173088 | 0.741354 | 0.852921 | 0.813962 | 3.193263 | 1.790178 | 4.727195 | 1.043399 | 2.783401 | 3.69522 | 0.798729 | 1.809553 | 1.505896 | 3.089359 | 2.198589 | 1.200214 |
| **ILF3** | 242.9018 | 281.7495 | 197.1107 | 295.1277 | 197.4724 | 211.8881 | 322.1365 | 265.9081 | 255.9494 | 323.2305 | 256.7292 | 275.933 | 260.5703 | 210.0476 | 278.9045 | 295.8036 | 216.1775 | 213.7949 | 263.5807 | 245.6469 | 227.3902 | 222.4338 | 288.4549 | 270.8483 |
| **IRAK1** | 146.3775 | 127.0434 | 80.95617 | 139.1659 | 101.8873 | 114.8469 | 146.145 | 165.4267 | 102.2406 | 137.1505 | 110.8797 | 100.1173 | 79.83158 | 86.52529 | 89.8167 | 65.21244 | 64.01822 | 69.68129 | 66.29453 | 83.69184 | 53.20831 | 70.02546 | 72.99317 | 68.81228 |
| **IRAK2** | 4.242826 | 6.147263 | 10.5595 | 7.198237 | 1.050385 | 9.793148 | 4.11676 | 8.577679 | 5.564117 | 5.18948 | 3.411684 | 4.069809 | 10.85709 | 8.354166 | 11.03012 | 6.782094 | 7.422402 | 4.223108 | 11.58157 | 7.238213 | 8.533408 | 4.119145 | 7.035486 | 11.60207 |
| **IRAK3** | 3.18212 | 6.147263 | 5.27975 | 4.798825 | 3.151155 | 2.670858 | 7.204331 | 8.577679 | 1.391029 | 2.224063 | 0.852921 | 2.441885 | 30.01667 | 25.65922 | 20.48451 | 29.21517 | 16.7004 | 30.08965 | 25.9587 | 21.71464 | 25.09826 | 33.46805 | 27.26251 | 38.00678 |
| **IRAK4** | 39.24614 | 25.61359 | 19.35908 | 39.5903 | 26.25962 | 37.39202 | 48.37193 | 34.31072 | 47.29499 | 37.80907 | 34.96976 | 40.69809 | 12.1344 | 16.70833 | 12.60585 | 14.08589 | 9.278002 | 17.94821 | 23.56251 | 17.64314 | 22.58843 | 24.19998 | 19.7873 | 18.00321 |
| **IRF1** | 61.52098 | 123.9698 | 77.43633 | 93.57708 | 96.63541 | 95.26062 | 113.2109 | 87.00217 | 48.68602 | 67.46324 | 71.64536 | 74.88448 | 85.57945 | 148.5848 | 84.30164 | 165.9004 | 111.336 | 147.8088 | 141.3751 | 71.47735 | 124.9893 | 62.30207 | 85.74499 | 104.4186 |
| **IRF3** | 7.424946 | 4.098175 | 3.519833 | 1.199706 | 7.352695 | 3.561145 | 8.233521 | 1.225383 | 9.04169 | 5.18948 | 1.705842 | 8.139618 | 7.663832 | 4.773809 | 5.515061 | 1.565099 | 1.8556 | 8.974105 | 5.19174 | 4.071495 | 4.015721 | 4.119145 | 5.276615 | 2.8005 |
| **IRF4** | 568.5387 | 1151.587 | 517.4155 | 1866.743 | 577.7117 | 1087.93 | 530.0329 | 1300.131 | 418.6998 | 522.6548 | 449.4893 | 530.7031 | 169.8816 | 108.0074 | 185.1485 | 146.0759 | 102.9858 | 145.1694 | 230.4334 | 137.0737 | 209.3195 | 217.2849 | 233.9299 | 252.4451 |
| **IRF5** | 29.69978 | 15.36816 | 45.75783 | 39.5903 | 26.25962 | 38.28231 | 39.10922 | 15.92998 | 26.42955 | 36.32636 | 43.49897 | 43.95394 | 90.05002 | 63.8497 | 84.30164 | 71.47284 | 43.60661 | 96.6036 | 90.25641 | 59.71526 | 83.32622 | 106.068 | 130.5962 | 105.6189 |
| **IRF7** | 592.9349 | 448.7502 | 508.6159 | 687.4316 | 617.6263 | 603.614 | 791.4472 | 589.4091 | 601.6201 | 727.2686 | 629.4557 | 698.3792 | 16.60497 | 44.15773 | 22.84811 | 29.73687 | 29.68961 | 33.25698 | 26.35807 | 22.61942 | 25.60022 | 20.59572 | 29.90082 | 27.20486 |
| **IRF8** | 3048.471 | 2802.127 | 2750.75 | 4057.406 | 2911.667 | 3524.643 | 4525.349 | 3215.404 | 3752.997 | 4984.866 | 4295.31 | 4564.698 | 131.5624 | 112.1845 | 178.0577 | 123.6428 | 82.57422 | 115.0797 | 170.5287 | 102.6921 | 158.621 | 183.8168 | 219.4192 | 252.045 |
| **IRF9** | 118.7991 | 161.8779 | 84.476 | 118.7709 | 105.0385 | 117.5178 | 162.612 | 91.9037 | 155.0998 | 146.7882 | 134.7615 | 141.6293 | 62.58796 | 91.2991 | 78.78658 | 104.8616 | 65.87382 | 83.93428 | 104.6335 | 51.11988 | 86.83998 | 68.99568 | 82.66696 | 109.6196 |
| **IRGM** | 6.364239 | 3.073631 | 8.799583 | 3.599119 | 3.151155 | 3.561145 | 3.08757 | 4.901531 | 5.564117 | 5.930835 | 3.411684 | 4.069809 | 3.193263 | 2.386905 | 1.575732 | 2.608498 | 2.783401 | 1.583666 | 2.396188 | 1.809553 | 3.513756 | 3.604252 | 2.638307 | 0.400071 |
| **ISG15** | 9.546359 | 123.9698 | 7.039667 | 16.79589 | 6.30231 | 10.68343 | 16.46704 | 11.02844 | 11.12823 | 22.98198 | 12.79381 | 9.767541 | 51.09221 | 242.8675 | 35.45396 | 69.38604 | 47.31781 | 73.9044 | 64.29771 | 70.12019 | 59.23189 | 45.31059 | 57.60304 | 65.61171 |
| **ITGA2B** | 3.18212 | 4.098175 | 3.519833 | 2.399412 | 4.20154 | 0.890286 | 5.14595 | 6.126914 | 0.695515 | 1.482709 | 2.558763 | 1.627924 | 3.193263 | 2.386905 | 3.151463 | 2.086798 | 3.711201 | 1.055777 | 5.19174 | 7.238213 | 6.023582 | 4.119145 | 2.198589 | 2.8005 |
| **ITGA4** | 113.4956 | 156.7552 | 95.0355 | 167.9589 | 85.08118 | 154.9098 | 185.2542 | 160.5251 | 142.5805 | 180.8905 | 147.5553 | 157.9086 | 189.0412 | 187.372 | 241.8748 | 166.9438 | 156.7982 | 210.0996 | 257.5902 | 214.8845 | 192.2527 | 183.3019 | 174.568 | 207.6371 |
| **ITGA5** | 12.72848 | 9.220894 | 22.87892 | 21.59471 | 24.15885 | 29.37944 | 22.64218 | 25.73304 | 26.42955 | 21.49928 | 23.02887 | 27.6747 | 190.9571 | 122.3289 | 129.9979 | 193.0288 | 120.614 | 175.259 | 254.3953 | 139.3356 | 253.9944 | 217.2849 | 324.9515 | 314.0561 |
| **ITGA6** | 6.364239 | 1.024544 | 5.27975 | 3.599119 | 7.352695 | 6.232003 | 8.233521 | 1.225383 | 3.477573 | 8.154897 | 5.117526 | 3.255847 | 5.747874 | 5.370535 | 5.515061 | 4.173596 | 1.8556 | 6.334663 | 3.993646 | 3.166718 | 4.517687 | 2.059572 | 2.638307 | 2.400428 |
| **ITGAE** | 121.9812 | 110.6507 | 75.67642 | 147.5639 | 93.48426 | 125.5303 | 158.4953 | 170.3282 | 98.06756 | 122.3235 | 153.5258 | 118.0245 | 56.84008 | 38.7872 | 51.99914 | 58.43035 | 43.60661 | 36.42431 | 59.50533 | 76.00124 | 54.21224 | 55.09356 | 70.35486 | 59.61064 |
| **ITGAL** | 94.40288 | 101.4298 | 175.9917 | 123.5697 | 116.5927 | 185.1795 | 208.9256 | 131.116 | 133.5388 | 163.8393 | 168.0254 | 140.8154 | 139.2263 | 103.2336 | 151.2702 | 87.12382 | 82.57422 | 177.3706 | 132.5891 | 142.5023 | 206.3077 | 144.685 | 163.575 | 134.0239 |
| **ITGAM** | 11.66777 | 8.19635 | 12.31942 | 2.399412 | 12.60462 | 13.35429 | 13.37947 | 7.352296 | 11.82375 | 11.86167 | 11.94089 | 8.953579 | 58.11739 | 38.7872 | 48.84768 | 77.73323 | 55.66801 | 49.09364 | 58.30724 | 70.57258 | 70.77709 | 57.66803 | 68.59599 | 71.21271 |
| **ITGAX** | 10.60707 | 11.26998 | 5.27975 | 5.998531 | 6.30231 | 16.91544 | 8.233521 | 19.60612 | 11.82375 | 9.637606 | 11.08797 | 15.46527 | 841.7442 | 643.8675 | 552.2939 | 723.5972 | 352.5641 | 599.6814 | 744.4157 | 628.8198 | 617.9191 | 634.8632 | 795.4496 | 661.7181 |
| **ITGB1** | 161.2274 | 142.4116 | 123.1942 | 193.1527 | 121.8447 | 153.1292 | 169.8164 | 196.0612 | 153.0132 | 156.4258 | 179.9663 | 229.5372 | 206.9235 | 161.7128 | 161.5125 | 175.291 | 121.5418 | 164.7012 | 259.1877 | 199.5032 | 214.3391 | 214.7104 | 227.3341 | 263.6471 |
| **ITGB2** | 211.0806 | 168.0252 | 175.9917 | 205.1498 | 135.4997 | 122.8595 | 233.6261 | 176.4551 | 170.4011 | 168.2874 | 213.2302 | 198.6067 | 1037.172 | 830.0461 | 735.0788 | 799.7654 | 687.5 | 693.1177 | 1006 | 905.6814 | 995.8989 | 853.6928 | 931.3225 | 1048.987 |
| **ITLN1** | 2.121413 | 1.024544 | 8.799583 | 1.199706 | 1.050385 | 2.670858 | 4.11676 | 4.901531 | 2.782058 | 7.413543 | 3.411684 | 1.627924 | 6.386526 | 5.370535 | 2.363597 | 2.086798 | 5.566801 | 2.639443 | 3.194917 | 2.71433 | 2.007861 | 3.089359 | 1.319154 | 1.200214 |
| **ITLN2** | 5.303533 | 2.049088 | 1.759917 | 1.199706 | 3.151155 | 0.890286 | 2.05838 | 2.450765 | 2.086544 | 2.224063 | 2.558763 | 3.255847 | 0.638653 | 1.790178 | 0.787866 | 2.608498 | 5.566801 | 1.055777 | 1.198094 | 1.809553 | 1.00393 | 2.574466 | 1.319154 | 0.400071 |
| **JAK1** | 323.5155 | 307.3631 | 219.9896 | 411.4992 | 273.1001 | 321.3933 | 376.6836 | 384.7702 | 290.7251 | 304.6966 | 356.521 | 293.8402 | 313.5784 | 251.2217 | 224.5418 | 263.4583 | 161.4372 | 222.769 | 251.2004 | 257.409 | 217.8529 | 233.2466 | 279.2209 | 279.2498 |
| **JAK2** | 181.3808 | 167.0006 | 153.1128 | 209.9486 | 157.5577 | 213.6687 | 359.1873 | 203.4135 | 241.3436 | 252.0605 | 254.1704 | 314.1892 | 376.8051 | 312.6845 | 449.0835 | 383.9709 | 267.2065 | 389.0539 | 392.5754 | 300.8382 | 457.7922 | 367.6337 | 412.8951 | 404.8723 |
| **JAK3** | 33.94261 | 39.95721 | 42.238 | 40.79001 | 47.26732 | 50.74631 | 40.13841 | 53.91684 | 36.86227 | 50.41209 | 34.11684 | 30.93055 | 68.97448 | 53.70535 | 62.2414 | 74.60303 | 115.975 | 67.04185 | 59.10597 | 112.1923 | 63.74958 | 64.36164 | 46.61009 | 60.41078 |
| **KCNJ2** | 8.485652 | 3.073631 | 10.5595 | 1.199706 | 5.251925 | 8.902862 | 2.05838 | 2.450765 | 6.955146 | 4.448126 | 3.411684 | 6.511694 | 6.386526 | 2.983631 | 3.939329 | 4.173596 | 9.278002 | 4.223108 | 5.19174 | 2.261942 | 10.54127 | 5.663824 | 5.276615 | 8.001428 |
| **KIR3DL1** | 1.060707 | 4.098175 | 7.039667 | 2.399412 | 3.151155 | 3.561145 | 2.05838 | 9.803062 | 2.086544 | 0.741354 | 0.852921 | 1.627924 | 0.638653 | 4.177083 | 3.939329 | 2.086798 | 6.494602 | 2.639443 | 2.795553 | 2.71433 | 2.509826 | 3.089359 | 1.319154 | 2.000357 |
| **KIR3DL2** | 6.364239 | 11.26998 | 8.799583 | 4.798825 | 6.30231 | 5.341717 | 4.11676 | 2.450765 | 7.650661 | 5.18948 | 6.823368 | 8.139618 | 6.386526 | 4.773809 | 5.515061 | 4.173596 | 6.494602 | 3.69522 | 4.792376 | 4.523883 | 6.525547 | 4.119145 | 3.957461 | 5.601 |
| **KIR3DL3** | 2.121413 | 3.073631 | 1.759917 | 3.599119 | 1.050385 | 0.890286 | 1.02919 | 2.450765 | 0.695515 | 4.448126 | 3.411684 | 1.627924 | 3.193263 | 1.790178 | 2.363597 | 2.086798 | 2.783401 | 1.583666 | 1.198094 | 4.071495 | 2.509826 | 2.059572 | 1.758872 | 2.000357 |
| **KIR_Activating_Subgroup_1** | 16.9713 | 16.3927 | 22.87892 | 14.39647 | 21.0077 | 14.24458 | 9.262711 | 13.47921 | 16.69235 | 21.49928 | 14.49966 | 10.5815 | 14.68901 | 12.53125 | 12.60585 | 10.95569 | 8.350202 | 11.08566 | 10.78285 | 12.66687 | 12.04716 | 14.9319 | 12.3121 | 10.40186 |
| **KIR_Activating_Subgroup_2** | 6.364239 | 1.024544 | 10.5595 | 3.599119 | 2.10077 | 4.451431 | 2.05838 | 6.126914 | 2.782058 | 2.224063 | 3.411684 | 6.511694 | 4.470568 | 4.773809 | 3.939329 | 3.651897 | 8.350202 | 3.69522 | 2.795553 | 1.809553 | 3.011791 | 1.029786 | 5.716332 | 2.000357 |
| **KIR_Inhibiting_Subgroup_1** | 3.18212 | 4.098175 | 3.519833 | 1.199706 | 6.30231 | 2.670858 | 2.05838 | 3.676148 | 3.477573 | 2.965417 | 6.823368 | 1.627924 | 0.638653 | 3.580357 | 4.727195 | 2.086798 | 6.494602 | 2.639443 | 2.396188 | 2.71433 | 4.015721 | 3.089359 | 1.758872 | 2.8005 |
| **KIR_Inhibiting_Subgroup_2** | 3.18212 | 3.073631 | 7.039667 | 5.998531 | 4.20154 | 3.561145 | 5.14595 | 4.901531 | 4.173088 | 3.706772 | 0.852921 | 1.627924 | 4.470568 | 1.790178 | 3.151463 | 3.130197 | 3.711201 | 3.167331 | 4.393011 | 2.71433 | 4.517687 | 3.604252 | 2.638307 | 4.400786 |
| **KIT** | 5.303533 | 6.147263 | 5.27975 | 4.798825 | 6.30231 | 5.341717 | 1.02919 | 7.352296 | 2.086544 | 1.482709 | 5.117526 | 3.255847 | 23.63015 | 5.967261 | 10.24226 | 4.173596 | 0.9278 | 6.334663 | 8.786022 | 10.85732 | 16.06289 | 15.44679 | 15.39013 | 15.20271 |
| **KLRAP1** | 6.364239 | 3.073631 | 5.27975 | 11.99706 | 8.403079 | 4.451431 | 6.175141 | 3.676148 | 7.650661 | 6.672189 | 4.264605 | 3.255847 | 7.025179 | 5.370535 | 3.151463 | 4.173596 | 8.350202 | 2.111554 | 3.194917 | 2.71433 | 6.023582 | 3.089359 | 4.836897 | 3.600643 |
| **KLRB1** | 3.18212 | 2.049088 | 1.759917 | 9.597649 | 6.30231 | 3.561145 | 1.02919 | 2.450765 | 5.564117 | 1.482709 | 3.411684 | 7.325656 | 5.109221 | 5.967261 | 3.151463 | 5.216995 | 8.350202 | 6.334663 | 6.389834 | 10.40493 | 9.537338 | 4.634038 | 5.276615 | 11.60207 |
| **KLRC1** | 2.121413 | 7.171807 | 3.519833 | 5.998531 | 4.20154 | 6.232003 | 2.05838 | 2.450765 | 1.391029 | 1.482709 | 5.970447 | 4.883771 | 1.277305 | 4.773809 | 3.939329 | 2.608498 | 5.566801 | 1.583666 | 3.993646 | 6.333436 | 3.513756 | 2.059572 | 2.198589 | 2.8005 |
| **KLRC2** | 4.242826 | 4.098175 | 5.27975 | 1.199706 | 1.050385 | 3.561145 | 1.02919 | 2.450765 | 3.477573 | 1.482709 | 3.411684 | 6.511694 | 3.193263 | 1.790178 | 3.939329 | 3.130197 | 3.711201 | 2.639443 | 0.798729 | 1.357165 | 2.509826 | 3.604252 | 1.758872 | 3.600643 |
| **KLRC3** | 6.364239 | 4.098175 | 8.799583 | 2.399412 | 5.251925 | 3.561145 | 5.14595 | 7.352296 | 2.086544 | 4.448126 | 1.705842 | 4.069809 | 1.915958 | 3.580357 | 3.939329 | 4.695296 | 8.350202 | 3.69522 | 4.792376 | 4.523883 | 3.011791 | 3.604252 | 2.638307 | 4.000714 |
| **KLRC4** | 19.09272 | 3.073631 | 5.27975 | 5.998531 | 3.151155 | 4.451431 | 9.262711 | 13.47921 | 9.737204 | 2.965417 | 3.411684 | 13.83735 | 4.470568 | 4.773809 | 4.727195 | 2.608498 | 7.422402 | 3.167331 | 5.19174 | 9.047766 | 3.011791 | 2.574466 | 3.957461 | 3.600643 |
| **KLRD1** | 3.18212 | 5.122719 | 8.799583 | 3.599119 | 7.352695 | 6.232003 | 1.02919 | 3.676148 | 2.782058 | 2.224063 | 5.970447 | 4.069809 | 4.470568 | 4.177083 | 7.878658 | 5.738695 | 3.711201 | 5.806774 | 5.19174 | 6.785825 | 5.521617 | 8.23829 | 4.397179 | 8.001428 |
| **KLRF1** | 4.242826 | 2.049088 | 5.27975 | 13.19677 | 8.403079 | 1.780572 | 9.262711 | 1.225383 | 31.99367 | 9.637606 | 8.529209 | 3.255847 | 4.470568 | 2.386905 | 2.363597 | 6.782094 | 3.711201 | 4.750997 | 5.591105 | 4.071495 | 13.55306 | 9.268076 | 13.19154 | 8.801571 |
| **KLRF2** | 4.242826 | 13.31907 | 21.119 | 20.395 | 14.70539 | 22.25715 | 20.5838 | 34.31072 | 15.99684 | 21.49928 | 54.58694 | 18.72112 | 1.277305 | 5.370535 | 3.939329 | 3.130197 | 3.711201 | 3.167331 | 3.594282 | 4.976272 | 5.521617 | 1.544679 | 1.758872 | 1.600286 |
| **KLRG1** | 7.424946 | 4.098175 | 7.039667 | 1.199706 | 7.352695 | 2.670858 | 1.02919 | 6.126914 | 6.259631 | 2.224063 | 1.705842 | 0.813962 | 1.915958 | 11.3378 | 6.302927 | 8.347192 | 13.917 | 5.278885 | 7.987293 | 6.333436 | 7.529478 | 7.208504 | 8.794358 | 7.201285 |
| **KLRG2** | 9.546359 | 10.24544 | 12.31942 | 5.998531 | 1.050385 | 4.451431 | 4.11676 | 6.126914 | 4.868602 | 7.413543 | 4.264605 | 6.511694 | 6.386526 | 1.790178 | 6.302927 | 3.130197 | 9.278002 | 6.862551 | 5.19174 | 4.071495 | 6.023582 | 3.089359 | 2.198589 | 0.800143 |
| **KLRK1** | 15.9106 | 5.122719 | 3.519833 | 11.99706 | 2.10077 | 6.232003 | 14.40866 | 14.70459 | 9.737204 | 1.482709 | 8.529209 | 13.83735 | 7.025179 | 4.177083 | 4.727195 | 5.216995 | 4.639001 | 1.583666 | 5.99047 | 9.047766 | 7.027513 | 5.148931 | 2.198589 | 10.80193 |
| **LAG3** | 7.424946 | 7.171807 | 3.519833 | 8.397943 | 3.151155 | 8.902862 | 5.14595 | 2.450765 | 7.650661 | 7.413543 | 6.823368 | 9.767541 | 3.831916 | 2.983631 | 0.787866 | 4.173596 | 3.711201 | 3.69522 | 1.996823 | 1.809553 | 5.019652 | 2.059572 | 2.198589 | 5.200928 |
| **LAIR1** | 461.4073 | 324.7804 | 406.5408 | 517.0734 | 336.1232 | 406.8608 | 684.4114 | 357.8118 | 452.78 | 553.7917 | 489.5766 | 612.0992 | 15.96632 | 11.93452 | 22.84811 | 17.73778 | 18.556 | 25.86654 | 19.1695 | 6.785825 | 27.60808 | 16.47658 | 19.34759 | 20.00357 |
| **LAMP3** | 5.303533 | 5.122719 | 5.27975 | 2.399412 | 4.20154 | 5.341717 | 3.08757 | 6.126914 | 2.086544 | 5.18948 | 2.558763 | 4.883771 | 2.554611 | 4.773809 | 3.939329 | 3.651897 | 4.639001 | 5.806774 | 4.393011 | 4.523883 | 3.011791 | 6.178717 | 3.078025 | 2.000357 |
| **LCK** | 3.18212 | 3.073631 | 10.5595 | 4.798825 | 2.10077 | 3.561145 | 7.204331 | 1.225383 | 4.868602 | 5.930835 | 5.117526 | 5.697732 | 3.193263 | 3.580357 | 4.727195 | 2.608498 | 6.494602 | 3.167331 | 5.591105 | 7.690601 | 1.00393 | 6.178717 | 3.078025 | 3.600643 |
| **LCP2** | 46.67109 | 39.95721 | 49.27767 | 51.58737 | 64.07348 | 89.9189 | 109.0941 | 36.76148 | 96.67653 | 76.35949 | 70.79244 | 82.21014 | 313.5784 | 305.5238 | 285.2074 | 290.0649 | 239.3725 | 362.1315 | 396.1697 | 290.4333 | 362.4189 | 351.1571 | 350.0154 | 363.2648 |
| **LEF1** | 3.18212 | 6.147263 | 1.759917 | 5.998531 | 7.352695 | 5.341717 | 2.05838 | 3.676148 | 4.173088 | 5.18948 | 2.558763 | 0.813962 | 3.193263 | 2.983631 | 3.151463 | 1.565099 | 5.566801 | 2.111554 | 5.19174 | 3.619107 | 2.007861 | 3.089359 | 3.517743 | 1.200214 |
| **LGALS3** | 5.303533 | 5.122719 | 8.799583 | 5.998531 | 5.251925 | 11.57372 | 5.14595 | 3.676148 | 8.346175 | 2.965417 | 4.264605 | 6.511694 | 314.8557 | 274.494 | 273.3894 | 273.3706 | 135.4588 | 242.3008 | 307.1114 | 308.0764 | 333.3049 | 359.3954 | 336.8239 | 307.6549 |
| **LIF** | 1.060707 | 7.171807 | 10.5595 | 3.599119 | 11.55423 | 3.561145 | 5.14595 | 7.352296 | 2.782058 | 6.672189 | 2.558763 | 4.069809 | 2.554611 | 2.386905 | 3.939329 | 3.130197 | 3.711201 | 2.639443 | 1.198094 | 5.42866 | 3.011791 | 3.089359 | 3.517743 | 2.8005 |
| **LILRA1** | 18.03201 | 11.26998 | 19.35908 | 7.198237 | 17.85654 | 10.68343 | 27.78813 | 20.83151 | 20.86544 | 25.9474 | 8.529209 | 38.2562 | 54.92413 | 54.8988 | 33.09036 | 54.25675 | 54.74021 | 63.87451 | 75.87928 | 52.92943 | 60.73779 | 57.66803 | 73.43289 | 68.41221 |
| **LILRA2** | 11.66777 | 6.147263 | 7.039667 | 10.79736 | 6.30231 | 16.02515 | 9.262711 | 14.70459 | 18.08338 | 11.86167 | 11.08797 | 26.04678 | 134.7557 | 91.89583 | 91.39244 | 108.5135 | 73.29622 | 124.5817 | 145.7681 | 114.9066 | 165.1465 | 143.1403 | 149.9438 | 169.2302 |
| **LILRA3** | 9.546359 | 10.24544 | 14.07933 | 11.99706 | 26.25962 | 11.57372 | 12.35028 | 3.676148 | 11.82375 | 13.34438 | 9.38213 | 9.767541 | 8.302484 | 13.7247 | 8.666524 | 7.825493 | 15.7726 | 11.61355 | 9.984116 | 18.54792 | 4.517687 | 7.723397 | 10.99295 | 6.001071 |
| **LILRA4** | 1850.933 | 1040.936 | 1172.105 | 1408.455 | 1037.78 | 1336.32 | 1599.361 | 1611.378 | 1656.716 | 1663.599 | 1363.821 | 1118.383 | 6.386526 | 4.773809 | 7.878658 | 5.738695 | 5.566801 | 5.278885 | 6.789199 | 3.166718 | 13.05109 | 7.208504 | 7.914922 | 7.201285 |
| **LILRA5** | 54.09603 | 61.47263 | 63.357 | 139.1659 | 51.46886 | 73.89375 | 96.74387 | 90.67832 | 74.42006 | 61.53241 | 82.73333 | 93.6056 | 40.23512 | 111.5878 | 69.33219 | 265.5451 | 83.50202 | 79.18328 | 113.0202 | 133.0022 | 74.29085 | 84.95736 | 94.97906 | 147.6264 |
| **LILRA6** | 5.303533 | 3.073631 | 15.83925 | 5.998531 | 9.453464 | 5.341717 | 3.08757 | 1.225383 | 6.955146 | 2.224063 | 7.676288 | 3.255847 | 10.85709 | 14.32143 | 11.03012 | 32.86707 | 12.0614 | 12.66933 | 14.37713 | 12.21448 | 19.57664 | 19.05105 | 16.26956 | 14.40257 |
| **LILRB1** | 172.8952 | 205.9333 | 112.6347 | 151.163 | 133.3989 | 142.4458 | 264.5019 | 107.8337 | 147.4491 | 237.2334 | 189.3484 | 170.932 | 83.6635 | 67.43005 | 58.30207 | 70.42944 | 75.15182 | 72.32073 | 69.48945 | 47.50077 | 51.70241 | 41.19145 | 74.75204 | 53.2095 |
| **LILRB2** | 41.36755 | 39.95721 | 33.43842 | 61.18501 | 54.62002 | 42.73374 | 80.27683 | 46.56454 | 54.25014 | 74.87679 | 82.73333 | 58.60525 | 113.6802 | 109.7976 | 100.8468 | 153.3797 | 77.93522 | 137.251 | 154.5541 | 130.7402 | 121.9775 | 113.7914 | 151.2629 | 167.2298 |
| **LILRB3** | 6.364239 | 1.024544 | 1.759917 | 1.199706 | 1.050385 | 7.122289 | 3.08757 | 2.450765 | 4.173088 | 2.965417 | 1.705842 | 1.627924 | 111.1256 | 109.2009 | 90.60457 | 122.5994 | 73.29622 | 124.0538 | 103.4354 | 104.5017 | 95.87535 | 97.3148 | 121.8019 | 124.8223 |
| **LILRB4** | 762.648 | 484.6092 | 478.6973 | 778.6093 | 391.7936 | 624.9809 | 747.192 | 792.8226 | 664.2164 | 650.9091 | 675.5134 | 740.7052 | 199.8983 | 179.6146 | 161.5125 | 160.6835 | 135.4588 | 186.3447 | 186.1039 | 216.694 | 179.2016 | 148.8041 | 249.32 | 200.4358 |
| **LILRB5** | 4.242826 | 1.024544 | 3.519833 | 1.199706 | 1.050385 | 2.670858 | 6.175141 | 1.225383 | 1.391029 | 2.224063 | 3.411684 | 3.255847 | 5.747874 | 2.386905 | 4.727195 | 1.043399 | 3.711201 | 1.583666 | 2.396188 | 1.809553 | 2.007861 | 2.059572 | 1.319154 | 2.000357 |
| **LITAF** | 238.659 | 184.4179 | 181.2714 | 226.7445 | 205.8754 | 185.1795 | 279.9397 | 262.2319 | 196.8306 | 234.268 | 286.5814 | 259.6538 | 192.8731 | 316.2649 | 155.2096 | 287.4564 | 171.643 | 225.4084 | 216.4556 | 338.3865 | 162.1348 | 226.0381 | 202.7099 | 242.4433 |
| **LTA** | 11.66777 | 5.122719 | 15.83925 | 7.198237 | 8.403079 | 12.46401 | 11.32109 | 13.47921 | 11.12823 | 10.37896 | 7.676288 | 4.069809 | 4.470568 | 6.563988 | 5.515061 | 3.651897 | 4.639001 | 4.750997 | 3.194917 | 2.261942 | 5.019652 | 3.604252 | 1.758872 | 2.400428 |
| **LTB4R** | 12.72848 | 15.36816 | 26.39875 | 21.59471 | 12.60462 | 20.47658 | 18.52542 | 20.83151 | 21.56095 | 23.72334 | 23.02887 | 10.5815 | 37.04185 | 31.02976 | 31.51463 | 39.12746 | 35.25641 | 36.42431 | 47.52439 | 27.1433 | 29.61595 | 41.70634 | 40.45404 | 43.20771 |
| **LTB4R2** | 4.242826 | 7.171807 | 14.07933 | 7.198237 | 10.50385 | 0.890286 | 12.35028 | 4.901531 | 6.259631 | 11.86167 | 0.852921 | 6.511694 | 10.21844 | 4.773809 | 17.33305 | 10.43399 | 3.711201 | 5.278885 | 7.987293 | 7.238213 | 8.533408 | 10.81276 | 12.3121 | 12.80229 |
| **LTBR** | 33.94261 | 51.22719 | 26.39875 | 56.38619 | 36.76347 | 51.6366 | 62.7806 | 55.14222 | 53.55462 | 62.27376 | 57.1457 | 75.69844 | 129.6465 | 125.3125 | 108.7255 | 144.5108 | 141.9534 | 137.251 | 159.7459 | 163.3122 | 126.9972 | 151.8935 | 161.8162 | 169.6303 |
| **LTF** | 6.364239 | 7.171807 | 7.039667 | 1.199706 | 2.10077 | 4.451431 | 4.11676 | 7.352296 | 1.391029 | 2.965417 | 2.558763 | 4.883771 | 5.109221 | 4.177083 | 3.151463 | 2.608498 | 3.711201 | 5.806774 | 2.396188 | 5.42866 | 2.509826 | 2.574466 | 3.078025 | 2.8005 |
| **LY96** | 145.3168 | 54.30082 | 49.27767 | 239.9412 | 67.22464 | 137.1041 | 77.18926 | 151.9475 | 77.20212 | 137.1505 | 91.26254 | 80.58221 | 96.43655 | 82.34821 | 52.78701 | 112.6871 | 120.614 | 111.9124 | 95.84751 | 129.8354 | 86.33801 | 108.6424 | 82.66696 | 96.01714 |
| **MAF** | 8.485652 | 3.073631 | 7.039667 | 1.199706 | 8.403079 | 5.341717 | 3.08757 | 6.126914 | 3.477573 | 3.706772 | 5.117526 | 5.697732 | 4.470568 | 5.967261 | 7.878658 | 4.173596 | 15.7726 | 3.167331 | 3.594282 | 3.166718 | 7.529478 | 5.663824 | 2.638307 | 3.200571 |
| **MALT1** | 141.074 | 122.9453 | 147.833 | 152.3627 | 123.9454 | 135.3235 | 200.6921 | 150.7221 | 141.885 | 154.9431 | 137.3203 | 189.6531 | 115.5961 | 131.8765 | 126.0585 | 185.725 | 89.06882 | 105.0498 | 108.2278 | 129.3831 | 99.38911 | 108.6424 | 129.2771 | 150.0268 |
| **MAP4K1** | 235.4768 | 204.9088 | 153.1128 | 308.3245 | 214.2785 | 255.5121 | 311.8446 | 265.9081 | 265.6866 | 326.9373 | 262.6997 | 262.9096 | 240.1334 | 202.8869 | 222.1782 | 211.81 | 141.0256 | 193.2072 | 217.6537 | 198.5985 | 188.7389 | 219.3445 | 256.7952 | 254.0453 |
| **MAP4K2** | 65.7638 | 73.76715 | 43.99792 | 91.17767 | 47.26732 | 54.30746 | 76.16007 | 71.0722 | 47.29499 | 71.17001 | 60.55739 | 65.11694 | 38.31916 | 44.75446 | 30.72677 | 45.38786 | 27.83401 | 34.31276 | 35.54345 | 32.57196 | 31.12184 | 28.31912 | 46.61009 | 48.00857 |
| **MAP4K4** | 194.1093 | 175.197 | 112.6347 | 254.3377 | 127.0966 | 166.4835 | 225.3926 | 183.8074 | 167.619 | 197.9416 | 164.6137 | 214.8859 | 42.15107 | 34.01339 | 25.99957 | 46.43126 | 29.68961 | 26.39443 | 38.33901 | 47.50077 | 28.61202 | 28.31912 | 40.45404 | 42.40757 |
| **MAPK1** | 272.6016 | 204.9088 | 183.0313 | 369.5095 | 205.8754 | 262.6344 | 310.8154 | 297.768 | 245.5167 | 300.9899 | 360.7856 | 332.0964 | 229.2763 | 207.064 | 212.7238 | 263.98 | 169.7874 | 215.3785 | 252.3985 | 232.5276 | 227.3902 | 277.5274 | 299.0082 | 321.6574 |
| **MAPK11** | 8.485652 | 11.26998 | 14.07933 | 8.397943 | 21.0077 | 10.68343 | 12.35028 | 7.352296 | 12.51926 | 7.413543 | 10.23505 | 9.767541 | 7.025179 | 10.74107 | 10.24226 | 8.347192 | 14.8448 | 7.39044 | 3.993646 | 9.047766 | 12.04716 | 11.32765 | 7.914922 | 6.401143 |
| **MAPK14** | 41.36755 | 47.12901 | 61.59708 | 85.17914 | 68.27502 | 71.22289 | 131.7363 | 58.81837 | 100.8496 | 126.0302 | 127.9381 | 133.4897 | 122.6213 | 53.70535 | 112.6648 | 137.207 | 94.63562 | 102.9383 | 168.1325 | 79.16796 | 166.1505 | 155.4977 | 167.5325 | 188.8337 |
| **MAPKAPK2** | 1488.171 | 1346.251 | 1085.869 | 1478.038 | 1160.675 | 1199.215 | 1570.544 | 1438.599 | 1149.686 | 1364.833 | 1316.91 | 1430.131 | 106.655 | 129.4896 | 106.3619 | 115.8173 | 84.42982 | 79.18328 | 110.624 | 115.8114 | 76.29871 | 104.5233 | 105.972 | 108.0193 |
| **MARCO** | 5.303533 | 3.073631 | 3.519833 | 4.798825 | 4.20154 | 2.670858 | 1.02919 | 3.676148 | 5.564117 | 2.965417 | 4.264605 | 6.511694 | 10.21844 | 11.93452 | 10.24226 | 11.99909 | 10.2058 | 12.14144 | 15.97459 | 10.85732 | 18.07075 | 15.96169 | 20.22702 | 15.20271 |
| **MASP1** | 6.364239 | 2.049088 | 8.799583 | 2.399412 | 6.30231 | 3.561145 | 4.11676 | 2.450765 | 4.868602 | 5.930835 | 5.117526 | 0.813962 | 7.025179 | 4.773809 | 7.090792 | 1.565099 | 5.566801 | 1.055777 | 3.594282 | 3.166718 | 3.513756 | 2.574466 | 1.758872 | 2.400428 |
| **MASP2** | 4.242826 | 9.220894 | 8.799583 | 1.199706 | 6.30231 | 3.561145 | 1.02919 | 6.126914 | 4.173088 | 3.706772 | 3.411684 | 4.069809 | 5.747874 | 5.370535 | 3.939329 | 3.651897 | 4.639001 | 3.167331 | 2.396188 | 4.976272 | 3.513756 | 3.089359 | 2.638307 | 2.400428 |
| **MAVS** | 43.48897 | 40.98175 | 28.15867 | 43.18942 | 28.36039 | 33.83087 | 60.72221 | 36.76148 | 52.1636 | 61.53241 | 56.29278 | 54.53544 | 28.10072 | 25.65922 | 29.15104 | 22.95478 | 24.12281 | 29.56176 | 39.5371 | 36.19107 | 36.64346 | 31.40848 | 49.2484 | 39.207 |
| **MBL2** | 5.303533 | 1.024544 | 7.039667 | 2.399412 | 7.352695 | 0.890286 | 1.02919 | 2.450765 | 0.695515 | 2.224063 | 1.705842 | 1.627924 | 3.193263 | 4.773809 | 2.363597 | 2.086798 | 1.8556 | 1.055777 | 1.198094 | 0.904777 | 2.509826 | 2.059572 | 2.198589 | 3.600643 |
| **MBP** | 72.12804 | 60.44808 | 66.87683 | 104.3744 | 54.62002 | 65.88118 | 96.74387 | 55.14222 | 64.68286 | 61.53241 | 86.14502 | 90.34976 | 72.8064 | 47.14136 | 70.90792 | 75.64643 | 38.96761 | 50.14941 | 74.68119 | 47.95316 | 58.72993 | 49.94463 | 81.34781 | 70.81264 |
| **MCL1** | 580.2065 | 987.6602 | 366.0627 | 838.5946 | 720.5641 | 501.2311 | 778.0677 | 1073.435 | 548.0655 | 619.7722 | 488.7237 | 588.4944 | 607.9973 | 1185.098 | 787.8658 | 1823.34 | 926.8724 | 830.3687 | 726.0449 | 1131.423 | 644.0213 | 665.2419 | 1005.195 | 851.752 |
| **MIF** | 93.34217 | 91.1844 | 105.595 | 123.5697 | 75.62772 | 105.0538 | 125.5612 | 158.0744 | 114.7599 | 112.6859 | 123.6735 | 104.1871 | 115.5961 | 85.33184 | 89.02884 | 77.21153 | 86.28542 | 97.65938 | 110.2246 | 121.2401 | 112.4402 | 123.0595 | 113.8869 | 96.01714 |
| **MME** | 16.9713 | 12.29453 | 7.039667 | 25.19383 | 4.20154 | 15.13486 | 20.5838 | 17.15536 | 13.21478 | 23.72334 | 17.91134 | 35.00036 | 0.638653 | 2.386905 | 3.151463 | 3.130197 | 3.711201 | 4.750997 | 2.795553 | 2.261942 | 1.505896 | 1.544679 | 1.758872 | 1.200214 |
| **MR1** | 10.60707 | 7.171807 | 5.27975 | 8.397943 | 9.453464 | 8.902862 | 13.37947 | 8.577679 | 16.69235 | 14.08573 | 12.79381 | 9.767541 | 12.77305 | 13.7247 | 8.666524 | 14.08589 | 8.350202 | 10.02988 | 18.77014 | 7.690601 | 11.5452 | 10.29786 | 20.22702 | 22.00393 |
| **MRC1** | 7.424946 | 7.171807 | 12.31942 | 7.198237 | 10.50385 | 13.35429 | 11.32109 | 9.803062 | 7.650661 | 38.55042 | 8.529209 | 13.83735 | 93.88194 | 33.41666 | 69.33219 | 70.42944 | 83.50202 | 96.07572 | 130.9916 | 77.3584 | 35.13756 | 114.8212 | 154.7807 | 113.6203 |
| **MS4A1** | 3.18212 | 4.098175 | 10.5595 | 1.199706 | 3.151155 | 0.890286 | 1.02919 | 3.676148 | 2.782058 | 2.965417 | 0.852921 | 1.627924 | 2.554611 | 2.386905 | 2.363597 | 3.651897 | 0.9278 | 2.111554 | 2.795553 | 2.71433 | 4.517687 | 3.604252 | 1.319154 | 1.200214 |
| **MSR1** | 3.18212 | 3.073631 | 3.519833 | 3.599119 | 5.251925 | 1.780572 | 3.08757 | 2.450765 | 4.173088 | 2.965417 | 3.411684 | 3.255847 | 38.95781 | 14.32143 | 15.75732 | 8.868892 | 20.41161 | 24.81076 | 20.3676 | 19.00031 | 9.035373 | 8.753183 | 14.95041 | 11.60207 |
| **MUC1** | 6.364239 | 2.049088 | 10.5595 | 5.998531 | 6.30231 | 8.012575 | 1.02919 | 1.225383 | 4.173088 | 5.18948 | 6.823368 | 3.255847 | 3.193263 | 5.370535 | 5.515061 | 4.173596 | 6.494602 | 6.862551 | 1.597459 | 5.42866 | 3.011791 | 5.663824 | 4.397179 | 2.000357 |
| **MX1** | 299.1192 | 1622.877 | 256.9478 | 806.2025 | 274.1505 | 568.8929 | 594.8719 | 348.0087 | 587.0143 | 671.667 | 416.2254 | 501.4004 | 270.1501 | 903.4434 | 222.1782 | 311.4546 | 113.1916 | 286.1156 | 260.7851 | 142.5023 | 246.9669 | 183.3019 | 186.0007 | 205.6367 |
| **MYD88** | 337.3047 | 403.6703 | 267.5073 | 475.0836 | 290.9566 | 359.6756 | 459.0188 | 357.8118 | 454.171 | 426.2787 | 351.4034 | 453.3767 | 570.9555 | 567.4866 | 461.6894 | 501.3532 | 439.7773 | 569.5917 | 624.6063 | 564.1282 | 625.9506 | 493.7825 | 515.7891 | 552.0985 |
| **NCAM1** | 26.51766 | 23.56451 | 35.19833 | 19.1953 | 24.15885 | 16.02515 | 16.46704 | 17.15536 | 17.38787 | 12.60302 | 18.76426 | 25.23281 | 26.18476 | 16.70833 | 18.12091 | 14.08589 | 25.97841 | 15.30877 | 15.57522 | 18.09553 | 10.54127 | 23.17019 | 13.19154 | 21.20378 |
| **NCF4** | 101.8278 | 64.54626 | 102.0752 | 127.1689 | 97.6858 | 86.35776 | 164.6704 | 88.22756 | 123.1061 | 146.7882 | 170.5842 | 122.9082 | 40.87377 | 41.77083 | 81.93805 | 55.30015 | 42.67881 | 50.6773 | 70.68754 | 43.88167 | 61.23975 | 62.81696 | 66.83712 | 68.01214 |
| **NCR1** | 1.060707 | 1.024544 | 3.519833 | 1.199706 | 5.251925 | 0.890286 | 2.05838 | 1.225383 | 3.477573 | 0.741354 | 0.852921 | 1.627924 | 0.638653 | 0.596726 | 0.787866 | 1.043399 | 3.711201 | 1.583666 | 2.396188 | 0.452388 | 1.00393 | 2.574466 | 0.879436 | 2.8005 |
| **NFATC1** | 23.33554 | 21.51542 | 24.63883 | 20.395 | 16.80616 | 19.5863 | 34.99246 | 23.28227 | 23.6475 | 28.17146 | 30.70515 | 24.41885 | 10.85709 | 8.354166 | 19.69665 | 15.65099 | 7.422402 | 10.55777 | 10.78285 | 14.92881 | 14.05503 | 8.23829 | 19.34759 | 13.60243 |
| **NFATC2** | 50.91391 | 50.20265 | 49.27767 | 53.98678 | 48.31771 | 71.22289 | 76.16007 | 40.43763 | 35.47124 | 50.41209 | 92.96838 | 87.09391 | 167.327 | 122.9256 | 152.0581 | 133.0334 | 76.07962 | 111.3845 | 192.8931 | 126.6687 | 140.0483 | 122.0297 | 202.7099 | 141.6253 |
| **NFATC3** | 94.40288 | 114.7489 | 80.95617 | 113.9721 | 86.13156 | 96.15091 | 152.3201 | 110.2844 | 128.6702 | 116.3926 | 136.4674 | 137.5595 | 74.72236 | 73.99404 | 56.72634 | 82.42853 | 50.10121 | 62.29085 | 91.4545 | 73.28691 | 70.27513 | 91.65097 | 92.34075 | 100.0179 |
| **NFIL3** | 28.63908 | 14.34361 | 15.83925 | 13.19677 | 15.75577 | 13.35429 | 16.46704 | 14.70459 | 13.91029 | 11.86167 | 17.91134 | 21.97697 | 52.36952 | 81.75148 | 41.75689 | 144.5108 | 62.16262 | 75.48806 | 23.16315 | 98.16827 | 21.5845 | 32.95316 | 43.53207 | 30.8055 |
| **NFKB1** | 31.8212 | 34.83449 | 17.59917 | 27.59324 | 25.20924 | 41.84345 | 66.89736 | 33.08533 | 52.1636 | 33.36094 | 50.32234 | 41.51205 | 63.86526 | 42.96428 | 39.39329 | 64.16904 | 37.11201 | 61.23507 | 62.30088 | 59.71526 | 72.78495 | 65.90632 | 79.58894 | 90.01607 |
| **NFKB2** | 21.21413 | 27.66268 | 24.63883 | 31.19236 | 33.61232 | 37.39202 | 32.93408 | 19.60612 | 20.86544 | 37.06772 | 25.58763 | 42.32601 | 44.70568 | 39.98065 | 66.9686 | 61.56054 | 41.75101 | 46.45419 | 56.31041 | 55.19138 | 53.20831 | 63.33185 | 82.22724 | 59.21057 |
| **NFKBIA** | 430.6468 | 537.8855 | 216.4698 | 466.6857 | 423.3051 | 306.2584 | 394.1798 | 406.8271 | 267.7731 | 363.2636 | 313.022 | 341.8639 | 768.2991 | 663.5595 | 619.2625 | 994.3593 | 521.4237 | 602.3208 | 539.941 | 864.9665 | 508.4907 | 796.5396 | 683.3216 | 686.9226 |
| **NFKBIZ** | 213.202 | 175.197 | 68.63675 | 161.9603 | 155.457 | 119.2983 | 165.6996 | 182.582 | 97.37204 | 194.9762 | 145.8495 | 90.34976 | 104.739 | 84.73511 | 112.6648 | 180.508 | 101.1302 | 82.35061 | 85.06467 | 108.1208 | 50.69848 | 108.1276 | 102.4543 | 88.41578 |
| **NLRP3** | 9.546359 | 16.3927 | 10.5595 | 9.597649 | 7.352695 | 8.012575 | 20.5838 | 6.126914 | 7.650661 | 4.448126 | 10.23505 | 15.46527 | 102.1844 | 147.9881 | 144.9673 | 187.2901 | 84.42982 | 133.5558 | 174.5223 | 118.0734 | 130.009 | 135.4169 | 156.5396 | 172.0307 |
| **NOD1** | 13.78918 | 14.34361 | 12.31942 | 5.998531 | 8.403079 | 14.24458 | 23.67137 | 4.901531 | 14.60581 | 25.20605 | 14.49966 | 17.90716 | 14.05036 | 4.773809 | 16.54518 | 10.95569 | 9.278002 | 12.14144 | 15.57522 | 6.333436 | 9.537338 | 11.84254 | 10.55323 | 16.40293 |
| **NOD2** | 9.546359 | 18.44179 | 17.59917 | 5.998531 | 11.55423 | 7.122289 | 7.204331 | 17.15536 | 11.12823 | 14.08573 | 9.38213 | 8.953579 | 72.8064 | 106.814 | 77.99872 | 95.99271 | 73.29622 | 91.32472 | 125.7999 | 81.88229 | 123.9854 | 116.8807 | 149.5041 | 112.4201 |
| **NOS2** | 8.485652 | 7.171807 | 12.31942 | 10.79736 | 11.55423 | 8.902862 | 6.175141 | 6.126914 | 7.650661 | 2.965417 | 4.264605 | 4.883771 | 12.77305 | 5.967261 | 5.515061 | 4.695296 | 4.639001 | 5.278885 | 5.591105 | 6.785825 | 6.023582 | 5.663824 | 7.035486 | 5.200928 |
| **NOTCH1** | 30.76049 | 31.76086 | 26.39875 | 51.58737 | 35.71309 | 29.37944 | 42.19679 | 34.31072 | 33.3847 | 48.18803 | 46.91065 | 39.88413 | 14.68901 | 16.11161 | 44.12049 | 22.95478 | 19.48381 | 21.64343 | 27.55616 | 14.02404 | 22.58843 | 23.17019 | 24.6242 | 22.80407 |
| **NOTCH2** | 251.3874 | 243.8414 | 205.9103 | 320.3215 | 259.4451 | 414.8734 | 434.3182 | 354.1356 | 280.2924 | 327.6786 | 365.0502 | 369.5386 | 286.755 | 198.7098 | 214.2995 | 269.7187 | 150.3036 | 283.4762 | 370.211 | 252.8851 | 265.5396 | 252.8125 | 304.2848 | 297.6531 |
| **NT5E** | 91.22076 | 72.74261 | 105.595 | 49.18795 | 121.8447 | 59.64917 | 61.75141 | 20.83151 | 60.50977 | 83.77304 | 55.43986 | 61.86109 | 50.45356 | 57.88244 | 81.93805 | 45.90956 | 48.24561 | 42.23108 | 47.12503 | 55.19138 | 54.21224 | 59.21271 | 41.33348 | 35.60636 |
| **OAS1** | 114.5563 | 165.9761 | 95.0355 | 181.1556 | 97.6858 | 136.2138 | 212.0132 | 118.8621 | 214.2185 | 233.5266 | 232.8474 | 247.4444 | 84.30215 | 137.247 | 74.05939 | 87.12382 | 40.82321 | 133.0279 | 119.8094 | 68.76302 | 148.0797 | 87.01694 | 86.62442 | 105.2188 |
| **PAX5** | 6.364239 | 10.24544 | 12.31942 | 3.599119 | 6.30231 | 2.670858 | 6.175141 | 9.803062 | 2.086544 | 3.706772 | 5.117526 | 2.441885 | 6.386526 | 4.773809 | 5.515061 | 5.216995 | 19.48381 | 5.278885 | 4.393011 | 4.976272 | 6.525547 | 4.119145 | 5.716332 | 3.200571 |
| **PDCD1** | 2.121413 | 2.049088 | 1.759917 | 1.199706 | 2.10077 | 0.890286 | 4.11676 | 3.676148 | 4.868602 | 1.482709 | 1.705842 | 5.697732 | 1.915958 | 1.790178 | 1.575732 | 0.5217 | 3.711201 | 2.639443 | 0.798729 | 1.809553 | 0.501965 | 1.544679 | 1.319154 | 0.800143 |
| **PDCD1LG2** | 3.18212 | 5.122719 | 12.31942 | 2.399412 | 3.151155 | 1.780572 | 3.08757 | 2.450765 | 4.173088 | 3.706772 | 2.558763 | 4.883771 | 3.831916 | 5.967261 | 11.81799 | 8.347192 | 16.7004 | 9.501994 | 6.789199 | 5.881048 | 7.529478 | 2.574466 | 2.638307 | 2.400428 |
| **PDCD2** | 27.57837 | 22.53996 | 29.91858 | 26.39354 | 31.51155 | 32.94059 | 26.75894 | 44.11378 | 25.03853 | 24.46469 | 29.85223 | 32.55847 | 23.63015 | 23.27232 | 25.99957 | 17.21608 | 24.12281 | 23.2271 | 22.76378 | 23.52419 | 18.57271 | 18.02126 | 25.94335 | 30.00536 |
| **PDGFB** | 11.66777 | 5.122719 | 7.039667 | 4.798825 | 4.20154 | 5.341717 | 7.204331 | 6.126914 | 7.650661 | 7.413543 | 8.529209 | 7.325656 | 3.193263 | 7.160714 | 3.939329 | 3.130197 | 4.639001 | 5.278885 | 2.795553 | 4.976272 | 4.015721 | 4.634038 | 3.957461 | 1.600286 |
| **PDGFRB** | 6.364239 | 2.049088 | 7.039667 | 2.399412 | 5.251925 | 1.780572 | 3.08757 | 2.450765 | 6.955146 | 3.706772 | 2.558763 | 1.627924 | 2.554611 | 1.193452 | 0.787866 | 1.043399 | 2.783401 | 4.223108 | 3.194917 | 1.809553 | 1.505896 | 2.574466 | 2.198589 | 3.600643 |
| **PECAM1** | 648.0917 | 691.5671 | 448.7788 | 665.8369 | 478.9755 | 693.5329 | 845.9942 | 626.1706 | 806.7969 | 575.2909 | 632.0144 | 608.0294 | 323.1582 | 299.5565 | 233.2083 | 294.2385 | 181.8488 | 275.5578 | 348.246 | 335.2197 | 321.2577 | 296.0635 | 332.4267 | 388.8694 |
| **PIGR** | 25.45696 | 23.56451 | 31.6785 | 22.79442 | 34.6627 | 24.92801 | 16.46704 | 7.352296 | 18.77889 | 20.01657 | 15.35258 | 15.46527 | 15.32766 | 14.32143 | 18.90878 | 14.08589 | 9.278002 | 11.61355 | 13.17903 | 9.047766 | 15.56092 | 19.56594 | 9.234075 | 9.601714 |
| **PLA2G2A** | 5.303533 | 5.122719 | 5.27975 | 3.599119 | 10.50385 | 3.561145 | 5.14595 | 4.901531 | 4.173088 | 4.448126 | 5.970447 | 3.255847 | 5.747874 | 6.563988 | 3.151463 | 1.565099 | 6.494602 | 3.69522 | 3.594282 | 5.42866 | 3.011791 | 7.723397 | 5.276615 | 2.000357 |
| **PLA2G2E** | 4.242826 | 3.073631 | 3.519833 | 3.599119 | 6.30231 | 2.670858 | 8.233521 | 4.901531 | 4.868602 | 8.154897 | 7.676288 | 3.255847 | 7.663832 | 6.563988 | 9.45439 | 6.782094 | 9.278002 | 7.39044 | 8.786022 | 6.333436 | 6.525547 | 7.723397 | 5.716332 | 6.801214 |
| **PLAU** | 12.72848 | 18.44179 | 8.799583 | 25.19383 | 13.655 | 13.35429 | 13.37947 | 26.95842 | 5.564117 | 10.37896 | 8.529209 | 17.0932 | 7.663832 | 8.950892 | 9.45439 | 9.912291 | 6.494602 | 8.446217 | 3.993646 | 19.90509 | 3.513756 | 4.634038 | 6.15605 | 6.801214 |
| **PLAUR** | 16.9713 | 6.147263 | 5.27975 | 38.3906 | 13.655 | 24.03773 | 4.11676 | 41.66301 | 4.868602 | 14.82709 | 12.79381 | 16.27924 | 22.35284 | 53.70535 | 31.51463 | 86.60212 | 40.82321 | 21.64343 | 14.77649 | 67.85825 | 24.09433 | 27.80423 | 28.58166 | 25.2045 |
| **PML** | 35.00331 | 32.7854 | 15.83925 | 46.78854 | 32.56193 | 49.85603 | 47.34274 | 36.76148 | 44.51293 | 54.11887 | 47.76357 | 46.39582 | 45.98299 | 63.25297 | 55.15061 | 57.38695 | 36.18421 | 52.26097 | 53.51486 | 40.26256 | 51.20045 | 41.19145 | 58.04276 | 49.60885 |
| **POU2F2** | 24.39625 | 25.61359 | 24.63883 | 31.19236 | 26.25962 | 29.37944 | 23.67137 | 35.5361 | 22.25647 | 26.68876 | 27.29347 | 31.74451 | 104.739 | 82.94493 | 111.8769 | 89.73232 | 85.35762 | 101.8825 | 112.6208 | 93.19199 | 111.4363 | 102.4637 | 138.9508 | 106.8191 |
| **PPARG** | 4.242826 | 4.098175 | 1.759917 | 1.199706 | 4.20154 | 1.780572 | 3.08757 | 3.676148 | 2.086544 | 1.482709 | 5.117526 | 3.255847 | 3.193263 | 2.386905 | 2.363597 | 1.565099 | 5.566801 | 3.69522 | 2.396188 | 0.904777 | 1.00393 | 4.119145 | 3.517743 | 2.000357 |
| **PPBP** | 9.546359 | 8.19635 | 10.5595 | 23.99412 | 18.90693 | 6.232003 | 11.32109 | 25.73304 | 10.43272 | 10.37896 | 4.264605 | 10.5815 | 3.831916 | 10.74107 | 11.03012 | 33.38877 | 7.422402 | 6.862551 | 11.58157 | 43.42928 | 23.59236 | 8.753183 | 5.276615 | 10.40186 |
| **PRDM1** | 31.8212 | 25.61359 | 17.59917 | 39.5903 | 22.05808 | 22.25715 | 8.233521 | 62.49452 | 6.955146 | 5.930835 | 17.05842 | 15.46527 | 15.96632 | 14.32143 | 22.06024 | 39.12746 | 15.7726 | 22.17132 | 14.37713 | 24.42897 | 10.0393 | 13.90211 | 14.51069 | 10.80193 |
| **PRF1** | 8.485652 | 3.073631 | 14.07933 | 9.597649 | 5.251925 | 14.24458 | 11.32109 | 12.25383 | 6.259631 | 5.18948 | 5.970447 | 3.255847 | 8.941137 | 6.563988 | 7.090792 | 14.08589 | 14.8448 | 8.974105 | 15.97459 | 16.73837 | 14.55699 | 16.47658 | 14.07097 | 27.20486 |
| **PRKCD** | 257.7517 | 184.4179 | 202.3904 | 409.0998 | 191.1701 | 365.0173 | 554.7335 | 332.0787 | 436.7832 | 403.2967 | 414.5196 | 338.6081 | 238.8561 | 204.6771 | 220.6024 | 256.1545 | 158.6538 | 279.7809 | 347.0479 | 209.4558 | 298.1673 | 332.1061 | 401.0227 | 375.6671 |
| **PSMB10** | 195.17 | 129.0925 | 133.7537 | 167.9589 | 124.9958 | 163.8127 | 218.1883 | 175.2297 | 146.0581 | 127.5129 | 139.0261 | 150.5829 | 136.033 | 135.4568 | 118.9677 | 141.3806 | 89.06882 | 206.4044 | 154.1548 | 117.621 | 142.0561 | 104.5233 | 118.7238 | 125.2224 |
| **PSMB5** | 47.73179 | 58.399 | 54.55742 | 83.97943 | 81.93002 | 91.69947 | 128.6488 | 68.62143 | 105.0227 | 95.63471 | 104.9093 | 138.3735 | 41.51242 | 41.1741 | 44.12049 | 47.47466 | 40.82321 | 68.09762 | 87.86022 | 47.50077 | 81.82032 | 66.42121 | 82.22724 | 120.4215 |
| **PSMB7** | 347.9117 | 293.0195 | 269.2673 | 453.4889 | 297.2589 | 383.7133 | 437.4058 | 419.0809 | 349.8438 | 399.59 | 403.4316 | 441.9812 | 367.8639 | 347.2946 | 293.0861 | 333.366 | 295.0405 | 359.4921 | 388.5818 | 375.9347 | 367.4385 | 340.8592 | 382.5546 | 393.6703 |
| **PSMB8** | 726.584 | 588.0881 | 494.5366 | 848.1923 | 638.634 | 936.581 | 910.8332 | 741.3566 | 817.9252 | 679.8219 | 966.3594 | 871.753 | 808.5342 | 550.7782 | 500.2948 | 659.4282 | 488.9507 | 930.1396 | 661.3479 | 565.9378 | 719.8181 | 512.8335 | 747.0807 | 751.7342 |
| **PSMB9** | 164.4095 | 270.4796 | 230.5491 | 337.1174 | 191.1701 | 412.2025 | 291.2608 | 200.9628 | 248.9942 | 198.683 | 219.2007 | 235.2349 | 336.5699 | 393.2425 | 307.2677 | 382.9275 | 283.9069 | 644.5519 | 377.3996 | 310.7908 | 363.4228 | 242.5147 | 334.6253 | 288.4515 |
| **PSMC2** | 110.3135 | 107.5771 | 110.8748 | 129.5683 | 129.1973 | 129.0915 | 180.1083 | 111.5098 | 157.8818 | 159.3912 | 168.8783 | 153.0248 | 112.4029 | 133.0699 | 101.6347 | 121.0343 | 105.7692 | 150.4482 | 144.57 | 120.3353 | 150.5896 | 146.2296 | 161.8162 | 154.4276 |
| **PSMD7** | 289.5729 | 319.6577 | 221.7495 | 334.718 | 249.9916 | 303.5876 | 332.4284 | 305.1203 | 264.2955 | 303.9553 | 342.0213 | 284.8866 | 227.3603 | 253.6086 | 226.9054 | 238.9384 | 204.1161 | 239.6614 | 257.1908 | 235.2419 | 233.4138 | 235.3062 | 262.0719 | 281.2502 |
| **PTAFR** | 2.121413 | 4.098175 | 10.5595 | 5.998531 | 5.251925 | 5.341717 | 8.233521 | 12.25383 | 6.955146 | 4.448126 | 4.264605 | 4.069809 | 109.2096 | 40.57738 | 77.99872 | 146.5976 | 46.39001 | 84.46217 | 144.57 | 84.14423 | 130.5109 | 146.7445 | 165.3339 | 184.0328 |
| **PTGER4** | 16.9713 | 14.34361 | 14.07933 | 16.79589 | 28.36039 | 10.68343 | 13.37947 | 40.43763 | 13.91029 | 21.49928 | 14.49966 | 23.60489 | 63.86526 | 35.20684 | 62.2414 | 77.21153 | 39.89541 | 45.39842 | 73.48309 | 65.59631 | 55.21617 | 60.24249 | 62.00022 | 77.61385 |
| **PTGS2** | 6.364239 | 6.147263 | 10.5595 | 3.599119 | 9.453464 | 8.012575 | 9.262711 | 3.676148 | 9.737204 | 2.965417 | 6.823368 | 15.46527 | 8.941137 | 20.88541 | 29.15104 | 65.73414 | 28.76181 | 26.92232 | 26.75743 | 32.57196 | 19.57664 | 33.46805 | 21.10646 | 30.40543 |
| **PTK2** | 106.0707 | 63.52172 | 77.43633 | 75.58149 | 87.18195 | 124.6401 | 120.4152 | 80.87526 | 126.5837 | 149.0122 | 105.7622 | 130.2339 | 24.2688 | 22.07887 | 24.42384 | 45.38786 | 21.33941 | 36.42431 | 36.74155 | 47.95316 | 35.13756 | 36.04252 | 42.65263 | 43.20771 |
| **PTPN2** | 118.7991 | 108.6016 | 102.0752 | 148.7636 | 122.895 | 106.8343 | 178.0499 | 110.2844 | 132.8433 | 124.5475 | 131.3498 | 147.3271 | 92.60463 | 93.08928 | 107.9376 | 94.94931 | 121.5418 | 76.54384 | 95.84751 | 112.1923 | 91.35766 | 89.07651 | 90.58188 | 94.01678 |
| **PTPN22** | 118.7991 | 90.15985 | 95.0355 | 121.1703 | 85.08118 | 130.8721 | 153.3493 | 90.67832 | 116.1509 | 132.7024 | 112.5856 | 118.0245 | 74.72236 | 78.17112 | 72.48366 | 87.12382 | 62.16262 | 80.76695 | 107.8285 | 65.59631 | 92.86356 | 87.01694 | 109.4898 | 112.02 |
| **PTPN6** | 161.2274 | 136.2643 | 149.5929 | 160.7606 | 132.3485 | 178.0572 | 247.0056 | 158.0744 | 219.0871 | 237.9747 | 234.5533 | 238.4908 | 455.998 | 356.8422 | 397.8722 | 374.0586 | 301.5351 | 420.7272 | 487.2249 | 388.6016 | 458.2942 | 409.8549 | 528.9806 | 496.4886 |
| **PTPRC_all** | 866.5972 | 836.0277 | 737.4051 | 1064.139 | 706.9091 | 886.725 | 1239.145 | 1045.251 | 1081.525 | 1092.756 | 1113.915 | 1226.64 | 1532.128 | 1240.594 | 1210.95 | 1355.375 | 1086.454 | 1217.839 | 1572.698 | 1390.189 | 1420.561 | 1458.177 | 1586.502 | 1673.899 |
| **PYCARD** | 45.61038 | 27.66268 | 29.91858 | 46.78854 | 42.0154 | 46.29488 | 72.04331 | 45.33916 | 65.37837 | 54.11887 | 67.38075 | 69.18675 | 68.97448 | 56.09226 | 70.90792 | 63.12564 | 51.02901 | 63.34663 | 71.88564 | 64.69153 | 74.29085 | 63.84675 | 71.2343 | 77.61385 |
| **RAF1** | 71.06734 | 78.88987 | 56.31733 | 88.77826 | 72.47656 | 81.01604 | 95.71468 | 62.49452 | 92.50344 | 87.47981 | 92.11546 | 105.0011 | 82.38619 | 65.04315 | 58.30207 | 76.68983 | 55.66801 | 69.68129 | 84.6653 | 72.83452 | 77.30264 | 72.59993 | 104.6529 | 106.419 |
| **RAG1** | 6.364239 | 6.147263 | 8.799583 | 5.998531 | 8.403079 | 8.902862 | 5.14595 | 6.126914 | 3.477573 | 6.672189 | 6.823368 | 5.697732 | 5.109221 | 5.370535 | 3.939329 | 5.216995 | 6.494602 | 2.639443 | 3.194917 | 2.71433 | 6.525547 | 4.119145 | 2.198589 | 3.200571 |
| **RAG2** | 2.121413 | 9.220894 | 7.039667 | 4.798825 | 4.20154 | 5.341717 | 7.204331 | 2.450765 | 3.477573 | 2.965417 | 2.558763 | 3.255847 | 5.109221 | 5.370535 | 2.363597 | 6.260394 | 7.422402 | 3.167331 | 5.99047 | 2.261942 | 5.521617 | 3.089359 | 3.078025 | 4.000714 |
| **RARRES3** | 3.18212 | 5.122719 | 7.039667 | 7.198237 | 3.151155 | 8.902862 | 3.08757 | 7.352296 | 5.564117 | 7.413543 | 5.117526 | 4.883771 | 39.59646 | 35.80357 | 20.48451 | 35.99727 | 22.26721 | 78.65539 | 47.52439 | 28.04808 | 42.66704 | 20.08083 | 35.61715 | 34.80621 |
| **RELA** | 53.03533 | 59.42354 | 35.19833 | 75.58149 | 42.0154 | 59.64917 | 69.98493 | 51.46607 | 64.68286 | 51.15345 | 58.85155 | 73.25656 | 45.34434 | 53.70535 | 47.27195 | 56.86525 | 29.68961 | 43.81475 | 40.33583 | 52.47705 | 42.16508 | 31.92337 | 46.17038 | 48.00857 |
| **RELB** | 12.72848 | 34.83449 | 17.59917 | 20.395 | 23.10847 | 24.92801 | 23.67137 | 44.11378 | 22.95198 | 16.30979 | 32.411 | 35.81432 | 35.76455 | 35.20684 | 40.18116 | 31.30197 | 35.25641 | 30.08965 | 34.34536 | 36.64345 | 29.61595 | 29.8638 | 32.53912 | 26.80478 |
| **RNaseH2** | 16.9713 | 10.24544 | 3.519833 | 5.998531 | 14.70539 | 15.13486 | 14.40866 | 17.15536 | 11.82375 | 14.08573 | 11.94089 | 14.65131 | 12.77305 | 11.3378 | 11.81799 | 13.04249 | 11.1336 | 8.974105 | 13.97776 | 14.92881 | 11.5452 | 11.84254 | 13.19154 | 8.4015 |
| **RORC** | 9.546359 | 10.24544 | 10.5595 | 7.198237 | 8.403079 | 8.902862 | 3.08757 | 8.577679 | 3.477573 | 2.224063 | 2.558763 | 10.5815 | 5.109221 | 4.773809 | 13.39372 | 6.782094 | 9.278002 | 10.55777 | 7.587928 | 6.333436 | 6.023582 | 6.178717 | 3.517743 | 4.400786 |
| **RUNX1** | 54.09603 | 44.05538 | 45.75783 | 47.98825 | 48.31771 | 50.74631 | 55.57626 | 41.66301 | 54.94565 | 63.75647 | 48.61649 | 60.23317 | 35.76455 | 41.77083 | 44.12049 | 50.08315 | 26.90621 | 29.03387 | 39.5371 | 45.69122 | 29.61595 | 32.95316 | 44.85122 | 51.20914 |
| **S100A8** | 5.303533 | 6.147263 | 3.519833 | 2.399412 | 7.352695 | 59.64917 | 12.35028 | 3.676148 | 9.04169 | 9.637606 | 4.264605 | 3.255847 | 330.8221 | 416.5148 | 438.0534 | 561.3487 | 976.0459 | 824.034 | 455.2757 | 535.6278 | 535.0949 | 621.9909 | 472.6967 | 600.1071 |
| **S100A9** | 5.303533 | 3.073631 | 14.07933 | 2.399412 | 5.251925 | 76.56461 | 13.37947 | 8.577679 | 6.259631 | 8.154897 | 1.705842 | 4.069809 | 809.8115 | 1158.245 | 1176.284 | 1472.758 | 2522.689 | 1643.845 | 1087.869 | 1396.975 | 1367.353 | 1491.645 | 1197.352 | 1579.082 |
| **S1PR1** | 10.60707 | 14.34361 | 14.07933 | 10.79736 | 21.0077 | 9.793148 | 5.14595 | 8.577679 | 9.04169 | 13.34438 | 5.970447 | 4.883771 | 3.831916 | 8.354166 | 8.666524 | 5.216995 | 4.639001 | 5.806774 | 6.389834 | 7.690601 | 6.525547 | 7.723397 | 7.035486 | 5.601 |
| **SAMHD1** | 1452.107 | 1005.077 | 1045.391 | 1281.286 | 1064.04 | 1403.981 | 1781.528 | 1431.247 | 1461.972 | 1426.366 | 1626.52 | 1735.366 | 2350.242 | 1565.213 | 1600.156 | 2016.369 | 1585.611 | 2105.22 | 2577.1 | 2156.535 | 2206.137 | 2144.015 | 2299.285 | 2895.717 |
| **SELE** | 2.121413 | 4.098175 | 5.27975 | 4.798825 | 2.10077 | 7.122289 | 5.14595 | 3.676148 | 2.782058 | 4.448126 | 4.264605 | 1.627924 | 3.193263 | 2.983631 | 3.939329 | 2.608498 | 3.711201 | 4.223108 | 1.597459 | 4.523883 | 4.517687 | 2.574466 | 1.758872 | 3.600643 |
| **SELL** | 1270.726 | 1164.906 | 920.4364 | 1390.459 | 1089.249 | 1420.897 | 1450.129 | 1477.812 | 1299.917 | 1281.06 | 1562.551 | 1313.734 | 632.9048 | 523.3288 | 415.9932 | 705.8595 | 751.5182 | 622.9085 | 711.2684 | 685.3683 | 642.0135 | 612.2079 | 697.3926 | 862.1539 |
| **SELPLG** | 49.85321 | 35.85903 | 49.27767 | 87.57855 | 51.46886 | 48.96574 | 68.95574 | 51.46607 | 63.29183 | 60.79105 | 85.29209 | 99.30333 | 15.32766 | 10.14434 | 8.666524 | 18.25948 | 17.6282 | 13.7251 | 20.76696 | 12.21448 | 13.55306 | 18.02126 | 24.6242 | 28.40507 |
| **SERPING1** | 93.34217 | 114.7489 | 105.595 | 176.3568 | 105.0385 | 129.9818 | 180.1083 | 147.0459 | 98.76307 | 140.8573 | 119.4089 | 180.6995 | 17.88227 | 66.83333 | 18.12091 | 24.51988 | 13.917 | 38.53586 | 17.57204 | 13.11926 | 19.07468 | 16.47658 | 18.02843 | 11.60207 |
| **SH2D1A** | 2.121413 | 5.122719 | 7.039667 | 2.399412 | 4.20154 | 6.232003 | 1.02919 | 1.225383 | 2.086544 | 2.224063 | 0.852921 | 2.441885 | 4.470568 | 2.386905 | 3.151463 | 1.043399 | 2.783401 | 3.167331 | 3.594282 | 1.357165 | 1.00393 | 4.119145 | 3.957461 | 2.8005 |
| **SIGIRR** | 5.303533 | 10.24544 | 5.27975 | 8.397943 | 6.30231 | 9.793148 | 9.262711 | 8.577679 | 6.955146 | 13.34438 | 11.94089 | 8.953579 | 21.71419 | 26.25595 | 26.78744 | 22.95478 | 15.7726 | 21.64343 | 22.36442 | 23.52419 | 24.59629 | 33.98295 | 31.21997 | 30.8055 |
| **SKI** | 46.67109 | 29.71177 | 42.238 | 52.78707 | 39.91463 | 33.83087 | 30.8757 | 20.83151 | 31.29816 | 37.80907 | 34.11684 | 24.41885 | 57.47874 | 57.28571 | 42.54475 | 59.99545 | 40.82321 | 32.72909 | 55.51169 | 55.19138 | 44.17294 | 85.98715 | 74.31232 | 67.61207 |
| **SLAMF1** | 9.546359 | 5.122719 | 7.039667 | 10.79736 | 5.251925 | 2.670858 | 15.43785 | 4.901531 | 14.60581 | 14.08573 | 11.94089 | 12.20943 | 6.386526 | 8.950892 | 3.151463 | 2.086798 | 7.422402 | 1.583666 | 1.996823 | 0.904777 | 1.00393 | 7.208504 | 4.836897 | 3.600643 |
| **SLAMF6** | 10.60707 | 11.26998 | 12.31942 | 9.597649 | 7.352695 | 11.57372 | 17.49623 | 8.577679 | 12.51926 | 8.896252 | 8.529209 | 12.20943 | 7.025179 | 2.983631 | 2.363597 | 4.695296 | 2.783401 | 5.278885 | 3.594282 | 3.619107 | 5.019652 | 5.663824 | 5.276615 | 4.000714 |
| **SLAMF7** | 154.8632 | 121.9207 | 119.6743 | 247.1395 | 147.0539 | 244.8287 | 331.3992 | 167.8774 | 228.1288 | 153.4603 | 228.5828 | 258.0259 | 50.45356 | 28.04613 | 38.60543 | 29.21517 | 40.82321 | 44.87053 | 46.3263 | 36.19107 | 26.60415 | 21.11062 | 41.33348 | 47.6085 |
| **SLC2A1** | 68.94592 | 47.12901 | 35.19833 | 63.58443 | 39.91463 | 55.19774 | 53.51788 | 82.10064 | 45.90396 | 63.75647 | 70.79244 | 66.74486 | 5.747874 | 22.67559 | 11.03012 | 9.912291 | 20.41161 | 10.55777 | 9.185387 | 13.11926 | 10.54127 | 7.208504 | 7.475204 | 11.202 |
| **SMAD3** | 44.54967 | 46.10447 | 47.51775 | 77.9809 | 53.56963 | 47.18517 | 91.59792 | 90.67832 | 69.55146 | 108.9791 | 73.3512 | 96.04749 | 45.98299 | 30.43303 | 37.81756 | 26.08498 | 17.6282 | 31.67331 | 55.91105 | 26.23852 | 51.70241 | 45.82549 | 56.72361 | 36.00643 |
| **SMAD5** | 26.51766 | 33.80995 | 29.91858 | 59.98531 | 33.61232 | 34.72116 | 58.66383 | 39.21225 | 48.68602 | 48.18803 | 45.20481 | 51.27959 | 22.35284 | 21.48214 | 28.36317 | 23.99818 | 29.68961 | 25.86654 | 35.94282 | 27.1433 | 33.1297 | 28.31912 | 34.73771 | 33.20593 |
| **SOCS1** | 39.24614 | 69.66898 | 31.6785 | 99.57561 | 108.1896 | 89.9189 | 30.8757 | 82.10064 | 60.50977 | 56.34293 | 42.64605 | 61.04713 | 4.470568 | 11.3378 | 11.03012 | 7.825493 | 12.0614 | 9.501994 | 9.185387 | 7.238213 | 10.54127 | 20.08083 | 13.63125 | 23.20414 |
| **SOCS3** | 29.69978 | 53.27628 | 29.91858 | 274.7327 | 155.457 | 45.40459 | 36.02165 | 276.9365 | 21.56095 | 19.27521 | 28.14639 | 54.53544 | 43.42838 | 112.7812 | 82.72591 | 397.535 | 113.1916 | 87.6295 | 56.31041 | 164.6693 | 30.61988 | 51.48931 | 72.99317 | 83.61492 |
| **SPP1** | 4.242826 | 3.073631 | 5.27975 | 1.199706 | 2.10077 | 0.890286 | 1.02919 | 1.225383 | 2.086544 | 2.965417 | 2.558763 | 3.255847 | 3.193263 | 4.177083 | 3.939329 | 2.086798 | 1.8556 | 2.111554 | 1.597459 | 1.357165 | 2.509826 | 3.089359 | 4.397179 | 3.200571 |
| **SRC** | 62.58168 | 91.1844 | 61.59708 | 91.17767 | 73.52695 | 109.5052 | 136.8823 | 89.45294 | 111.2823 | 110.4618 | 88.70378 | 83.0241 | 16.60497 | 28.04613 | 30.72677 | 28.69347 | 24.12281 | 28.50598 | 30.35171 | 23.0718 | 27.10612 | 23.17019 | 26.38307 | 27.20486 |
| **STAT1** | 152.7417 | 481.5356 | 146.0731 | 277.1321 | 167.0112 | 365.9076 | 215.1007 | 144.5952 | 229.5198 | 192.7521 | 179.9663 | 207.5602 | 139.2263 | 330.5863 | 100.8468 | 244.1554 | 97.41903 | 433.9244 | 182.909 | 108.1208 | 192.2527 | 94.22544 | 116.5252 | 112.02 |
| **STAT2** | 350.0331 | 383.1794 | 227.0293 | 482.2819 | 287.8055 | 372.1396 | 391.0922 | 365.1641 | 283.77 | 407.7449 | 366.756 | 389.8877 | 205.0075 | 274.494 | 211.9359 | 254.5894 | 158.6538 | 268.6953 | 187.7014 | 226.1942 | 177.6957 | 188.9658 | 217.6603 | 230.4411 |
| **STAT3** | 183.5022 | 195.6879 | 126.714 | 266.3348 | 198.5228 | 234.1453 | 228.4802 | 269.5842 | 185.0069 | 232.0439 | 253.3175 | 216.5138 | 167.9656 | 180.808 | 118.1799 | 186.7684 | 161.4372 | 204.2929 | 171.7268 | 193.1698 | 145.5699 | 153.4381 | 187.3198 | 170.0303 |
| **STAT4** | 15.9106 | 33.80995 | 22.87892 | 16.79589 | 24.15885 | 27.59887 | 43.22598 | 17.15536 | 11.82375 | 11.86167 | 16.2055 | 29.30262 | 11.49575 | 10.14434 | 3.151463 | 15.65099 | 5.566801 | 6.334663 | 7.188564 | 7.690601 | 8.533408 | 7.723397 | 9.673793 | 8.001428 |
| **STAT5A** | 20.15342 | 23.56451 | 21.119 | 21.59471 | 14.70539 | 21.36687 | 26.75894 | 24.50765 | 27.82058 | 25.20605 | 34.96976 | 35.00036 | 45.34434 | 32.22321 | 31.51463 | 34.43217 | 25.97841 | 35.89642 | 51.51804 | 24.88136 | 46.68276 | 39.64677 | 60.68107 | 62.81121 |
| **STAT5B** | 111.3742 | 88.11077 | 59.83717 | 101.975 | 67.22464 | 104.1635 | 112.1817 | 94.35447 | 84.15727 | 114.1686 | 108.321 | 102.5592 | 104.1004 | 68.62351 | 77.21085 | 97.03611 | 62.16262 | 80.76695 | 91.05514 | 77.81079 | 79.3105 | 90.62119 | 88.82301 | 118.4211 |
| **STAT6** | 275.7837 | 239.7432 | 251.6681 | 387.5051 | 292.007 | 320.503 | 378.742 | 356.5864 | 237.1705 | 321.7478 | 287.4344 | 407.7948 | 446.4182 | 315.0714 | 372.6605 | 505.0051 | 326.5857 | 433.9244 | 468.4547 | 406.2447 | 341.3363 | 401.1017 | 543.931 | 539.6963 |
| **SYK** | 606.7241 | 501.0019 | 543.8143 | 634.6446 | 383.3905 | 480.7545 | 645.3022 | 650.6782 | 502.1615 | 590.118 | 603.868 | 765.1241 | 417.0402 | 310.8943 | 349.8124 | 363.6246 | 259.7841 | 378.4961 | 392.5754 | 368.6965 | 365.9326 | 315.6295 | 333.3061 | 409.6731 |
| **TAGAP** | 202.5949 | 184.4179 | 161.9123 | 199.1512 | 224.7824 | 259.0733 | 348.8954 | 220.5689 | 297.6802 | 273.5597 | 292.5519 | 299.5379 | 259.9316 | 230.3363 | 277.3288 | 296.847 | 244.0115 | 247.0518 | 409.7481 | 265.5519 | 432.694 | 433.0251 | 419.4909 | 492.4879 |
| **TAL1** | 4.242826 | 2.049088 | 7.039667 | 1.199706 | 5.251925 | 3.561145 | 3.08757 | 6.126914 | 2.782058 | 2.224063 | 2.558763 | 1.627924 | 4.470568 | 4.773809 | 4.727195 | 4.173596 | 5.566801 | 1.583666 | 1.597459 | 5.42866 | 2.007861 | 1.029786 | 3.078025 | 1.200214 |
| **TAP1** | 58.33886 | 79.91442 | 58.07725 | 99.57561 | 64.07348 | 121.0789 | 92.62711 | 62.49452 | 100.1541 | 68.2046 | 94.67422 | 74.88448 | 51.09221 | 56.09226 | 35.45396 | 64.69074 | 33.40081 | 83.93428 | 51.9174 | 59.26287 | 54.21224 | 32.95316 | 53.20586 | 44.40793 |
| **TAP2** | 75.31016 | 70.69352 | 58.07725 | 56.38619 | 53.56963 | 81.01604 | 71.01412 | 71.0722 | 57.0322 | 57.08428 | 52.02818 | 42.32601 | 55.56278 | 50.72172 | 31.51463 | 39.12746 | 28.76181 | 48.56575 | 43.13138 | 40.71495 | 46.68276 | 30.37869 | 43.09235 | 27.20486 |
| **TAPBP** | 421.1005 | 439.5293 | 369.5825 | 514.6739 | 365.534 | 478.0837 | 514.595 | 573.4791 | 409.6581 | 352.1433 | 446.9306 | 452.5627 | 270.1501 | 249.4315 | 249.7535 | 233.1997 | 189.2712 | 313.5658 | 283.1495 | 254.2422 | 241.9472 | 222.4338 | 224.6958 | 250.4447 |
| **TBK1** | 11.66777 | 19.46633 | 10.5595 | 20.395 | 15.75577 | 32.94059 | 29.84651 | 22.05689 | 18.77889 | 27.43011 | 23.02887 | 28.48866 | 53.64682 | 65.63988 | 38.60543 | 56.34355 | 35.25641 | 44.87053 | 51.9174 | 42.5245 | 58.72993 | 44.28081 | 55.84417 | 59.21057 |
| **TBX21** | 11.66777 | 10.24544 | 7.039667 | 5.998531 | 6.30231 | 4.451431 | 8.233521 | 7.352296 | 7.650661 | 8.154897 | 5.117526 | 7.325656 | 8.941137 | 4.773809 | 6.302927 | 4.173596 | 3.711201 | 5.278885 | 5.19174 | 8.595378 | 3.011791 | 6.178717 | 3.957461 | 8.001428 |
| **TCF4** | 865.5365 | 890.3286 | 733.8853 | 1191.308 | 789.8895 | 984.6565 | 1228.853 | 1013.392 | 976.5025 | 1191.356 | 1067.857 | 1157.454 | 35.76455 | 23.86905 | 33.87823 | 28.17177 | 25.97841 | 40.11953 | 41.53392 | 37.54823 | 46.1808 | 39.64677 | 39.13489 | 41.20736 |
| **TCF7** | 4.242826 | 2.049088 | 3.519833 | 1.199706 | 1.050385 | 3.561145 | 1.02919 | 2.450765 | 2.782058 | 2.965417 | 1.705842 | 4.069809 | 1.277305 | 0.596726 | 0.787866 | 4.695296 | 1.8556 | 1.583666 | 1.597459 | 2.71433 | 2.509826 | 4.119145 | 2.198589 | 2.8005 |
| **TFRC** | 149.5596 | 186.467 | 91.51567 | 135.5668 | 165.9608 | 137.9944 | 208.9256 | 226.6958 | 174.5742 | 189.7867 | 147.5553 | 151.3969 | 55.56278 | 42.96428 | 37.02969 | 49.03976 | 27.83401 | 27.4502 | 36.74155 | 42.97689 | 36.64346 | 46.34038 | 40.45404 | 42.40757 |
| **TGFB1** | 215.3234 | 151.6325 | 165.4322 | 297.5271 | 161.7593 | 232.3647 | 242.8889 | 259.7811 | 199.6127 | 205.3551 | 249.9058 | 253.9561 | 265.0408 | 264.9464 | 227.6932 | 272.8489 | 141.9534 | 235.4383 | 275.1622 | 231.1704 | 248.9747 | 282.1614 | 288.0152 | 294.4526 |
| **TGFBI** | 1324.822 | 1146.465 | 1024.272 | 1917.13 | 1050.385 | 1321.185 | 1287.517 | 1638.337 | 1469.622 | 1648.031 | 1602.638 | 1923.392 | 482.1827 | 356.8422 | 408.9024 | 484.1372 | 289.4737 | 427.5897 | 392.5754 | 451.9359 | 441.7294 | 456.1953 | 459.0655 | 530.4947 |
| **TGFBR1** | 25.45696 | 12.29453 | 21.119 | 32.39207 | 14.70539 | 18.69601 | 10.2919 | 22.05689 | 15.99684 | 15.56844 | 22.17594 | 20.34904 | 20.43688 | 19.09524 | 17.33305 | 19.82458 | 14.8448 | 26.39443 | 26.35807 | 10.40493 | 20.07861 | 24.19998 | 25.06392 | 19.6035 |
| **TGFBR2** | 282.1479 | 349.3694 | 211.19 | 440.2922 | 284.6543 | 382.8231 | 466.2231 | 209.5404 | 364.4497 | 403.2967 | 379.5498 | 396.3994 | 93.88194 | 76.38095 | 93.75603 | 137.7287 | 68.65722 | 100.2988 | 126.998 | 92.73961 | 111.4363 | 109.6722 | 152.1424 | 147.6264 |
| **THY1** | 2.121413 | 2.049088 | 3.519833 | 1.199706 | 5.251925 | 2.670858 | 1.02919 | 2.450765 | 4.868602 | 1.482709 | 2.558763 | 1.627924 | 3.831916 | 0.596726 | 3.151463 | 2.608498 | 5.566801 | 1.055777 | 1.597459 | 1.809553 | 2.509826 | 3.089359 | 2.638307 | 2.000357 |
| **TICAM1** | 45.61038 | 64.54626 | 28.15867 | 35.99119 | 47.26732 | 42.73374 | 78.21845 | 60.04375 | 47.29499 | 60.0497 | 55.43986 | 47.20978 | 49.81491 | 44.75446 | 56.72634 | 66.77754 | 41.75101 | 38.53586 | 61.90152 | 58.35809 | 57.726 | 50.45952 | 57.60304 | 48.00857 |
| **TIGIT** | 7.424946 | 6.147263 | 17.59917 | 8.397943 | 9.453464 | 11.57372 | 8.233521 | 3.676148 | 12.51926 | 6.672189 | 8.529209 | 11.39546 | 10.21844 | 13.7247 | 9.45439 | 9.390592 | 8.350202 | 7.918328 | 5.19174 | 14.92881 | 13.55306 | 9.782969 | 9.673793 | 6.801214 |
| **TIRAP** | 5.303533 | 8.19635 | 1.759917 | 7.198237 | 4.20154 | 11.57372 | 8.233521 | 7.352296 | 6.259631 | 8.896252 | 8.529209 | 3.255847 | 8.941137 | 7.160714 | 14.18158 | 5.738695 | 5.566801 | 7.39044 | 9.984116 | 7.238213 | 8.031443 | 7.208504 | 12.3121 | 10.40186 |
| **TLR1** | 55.15674 | 102.4544 | 38.71817 | 76.78119 | 86.13156 | 75.67432 | 142.0282 | 80.87526 | 69.55146 | 95.63471 | 58.85155 | 116.3965 | 94.52059 | 159.9226 | 127.6343 | 134.5985 | 152.1592 | 120.3586 | 176.9185 | 147.0262 | 141.0522 | 129.2382 | 137.192 | 174.0311 |
| **TLR2** | 33.94261 | 18.44179 | 45.75783 | 14.39647 | 29.41078 | 26.70858 | 18.52542 | 14.70459 | 16.69235 | 20.75792 | 19.61718 | 21.16301 | 95.15924 | 81.75148 | 113.4527 | 80.86343 | 94.63562 | 122.998 | 146.9662 | 95.00155 | 197.7743 | 156.5275 | 109.4898 | 136.4244 |
| **TLR3** | 24.39625 | 17.41724 | 17.59917 | 11.99706 | 13.655 | 16.91544 | 13.37947 | 6.126914 | 20.86544 | 10.37896 | 8.529209 | 16.27924 | 26.18476 | 18.49851 | 21.27238 | 20.34628 | 20.41161 | 24.81076 | 38.73837 | 19.00031 | 40.15721 | 24.19998 | 34.29799 | 34.80621 |
| **TLR4** | 4.242826 | 5.122719 | 5.27975 | 5.998531 | 5.251925 | 7.122289 | 6.175141 | 4.901531 | 2.782058 | 2.965417 | 6.823368 | 4.069809 | 51.09221 | 69.22023 | 49.63555 | 83.47192 | 78.86302 | 79.18328 | 88.25959 | 67.85825 | 71.27906 | 79.29354 | 90.58188 | 108.0193 |
| **TLR5** | 6.364239 | 5.122719 | 1.759917 | 4.798825 | 3.151155 | 3.561145 | 5.14595 | 4.901531 | 4.868602 | 5.18948 | 3.411684 | 4.883771 | 25.54611 | 19.69196 | 18.12091 | 19.82458 | 12.9892 | 23.2271 | 28.35489 | 28.50046 | 26.60415 | 26.25955 | 33.85828 | 30.8055 |
| **TLR7** | 92.28147 | 131.1416 | 116.1545 | 151.163 | 100.837 | 138.8846 | 145.1158 | 118.8621 | 120.324 | 149.0122 | 137.3203 | 153.8388 | 12.1344 | 6.563988 | 14.18158 | 9.912291 | 11.1336 | 15.83666 | 13.97776 | 9.500155 | 22.08647 | 14.41701 | 17.149 | 14.80264 |
| **TLR8** | 5.303533 | 5.122719 | 8.799583 | 2.399412 | 7.352695 | 13.35429 | 5.14595 | 4.901531 | 5.564117 | 5.18948 | 4.264605 | 4.069809 | 104.1004 | 106.814 | 92.1803 | 117.9041 | 95.56343 | 143.5857 | 163.7395 | 110.3828 | 123.4834 | 119.9701 | 101.5748 | 164.0293 |
| **TLR9** | 137.8918 | 94.25803 | 98.55533 | 146.3642 | 117.6431 | 141.5555 | 179.0791 | 139.6936 | 167.619 | 164.5807 | 167.1725 | 186.3972 | 6.386526 | 4.773809 | 9.45439 | 10.43399 | 18.556 | 4.750997 | 5.591105 | 7.238213 | 10.0393 | 10.29786 | 5.716332 | 2.400428 |
| **TMEM173** | 83.79581 | 63.52172 | 72.15658 | 185.9545 | 106.0889 | 113.0663 | 131.7363 | 137.2429 | 143.276 | 173.4769 | 129.644 | 211.6301 | 205.0075 | 135.4568 | 196.1786 | 208.1581 | 211.5385 | 234.3825 | 231.6315 | 200.408 | 301.1791 | 283.7061 | 288.4549 | 328.4586 |
| **TNF** | 11.66777 | 20.49088 | 10.5595 | 9.597649 | 17.85654 | 20.47658 | 19.55461 | 17.15536 | 13.21478 | 18.53386 | 6.823368 | 12.20943 | 23.63015 | 36.99702 | 31.51463 | 20.34628 | 37.11201 | 13.7251 | 39.5371 | 37.54823 | 50.69848 | 67.451 | 46.61009 | 84.81514 |
| **TNFAIP3** | 229.1126 | 233.596 | 121.4343 | 277.1321 | 129.1973 | 136.2138 | 183.1958 | 213.2166 | 159.9684 | 259.474 | 171.4371 | 218.9557 | 70.25179 | 105.0238 | 84.30164 | 185.725 | 102.058 | 165.2291 | 119.41 | 127.5735 | 100.895 | 169.9147 | 142.0289 | 132.8237 |
| **TNFAIP6** | 14.84989 | 6.147263 | 15.83925 | 4.798825 | 11.55423 | 5.341717 | 5.14595 | 4.901531 | 9.04169 | 4.448126 | 8.529209 | 4.883771 | 7.025179 | 10.14434 | 7.090792 | 8.347192 | 9.278002 | 8.446217 | 8.786022 | 12.66687 | 12.54913 | 7.208504 | 10.55323 | 9.601714 |
| **TNFRSF10C** | 1.060707 | 1.024544 | 1.759917 | 1.199706 | 2.10077 | 2.670858 | 1.02919 | 4.901531 | 0.695515 | 0.741354 | 4.264605 | 0.813962 | 2.554611 | 2.983631 | 4.727195 | 1.043399 | 0.9278 | 2.111554 | 2.396188 | 1.809553 | 2.007861 | 3.604252 | 6.15605 | 4.800857 |
| **TNFRSF11A** | 11.66777 | 13.31907 | 14.07933 | 13.19677 | 7.352695 | 17.80572 | 17.49623 | 9.803062 | 16.69235 | 13.34438 | 11.08797 | 6.511694 | 5.747874 | 4.773809 | 4.727195 | 3.130197 | 9.278002 | 5.278885 | 3.194917 | 5.881048 | 10.54127 | 6.69361 | 6.595768 | 4.000714 |
| **TNFRSF13B** | 1.060707 | 3.073631 | 3.519833 | 5.998531 | 1.050385 | 1.780572 | 1.02919 | 1.225383 | 2.086544 | 2.965417 | 2.558763 | 1.627924 | 0.638653 | 3.580357 | 0.787866 | 2.086798 | 1.8556 | 4.223108 | 1.198094 | 3.166718 | 1.505896 | 3.089359 | 3.517743 | 1.200214 |
| **TNFRSF13C** | 8.485652 | 7.171807 | 3.519833 | 9.597649 | 4.20154 | 9.793148 | 15.43785 | 8.577679 | 6.259631 | 17.7925 | 7.676288 | 20.34904 | 3.831916 | 1.193452 | 4.727195 | 1.565099 | 2.783401 | 2.111554 | 2.795553 | 3.166718 | 3.513756 | 1.029786 | 2.198589 | 2.8005 |
| **TNFRSF14** | 64.7031 | 54.30082 | 56.31733 | 77.9809 | 50.41848 | 72.11318 | 52.48869 | 51.46607 | 49.38154 | 53.37751 | 64.82199 | 70.00071 | 32.57128 | 32.22321 | 32.3025 | 44.86616 | 36.18421 | 62.81874 | 50.31995 | 33.92912 | 47.18473 | 37.0723 | 53.64558 | 35.60636 |
| **TNFRSF17** | 70.00663 | 37.90812 | 38.71817 | 50.38766 | 75.62772 | 84.57719 | 135.8531 | 53.91684 | 132.1478 | 133.4438 | 187.6426 | 142.4433 | 5.109221 | 7.75744 | 3.939329 | 6.260394 | 6.494602 | 8.974105 | 6.389834 | 5.42866 | 2.007861 | 6.178717 | 8.35464 | 5.601 |
| **TNFRSF1B** | 57.27815 | 63.52172 | 75.67642 | 81.58002 | 59.87194 | 64.99089 | 89.53954 | 60.04375 | 49.38154 | 45.22261 | 71.64536 | 60.23317 | 87.49541 | 153.3586 | 126.0585 | 159.6401 | 107.6248 | 125.1096 | 134.1865 | 137.9784 | 93.86749 | 119.9701 | 145.9863 | 120.0214 |
| **TNFRSF4** | 5.303533 | 5.122719 | 1.759917 | 3.599119 | 3.151155 | 1.780572 | 1.02919 | 2.450765 | 2.782058 | 3.706772 | 5.117526 | 3.255847 | 1.277305 | 5.370535 | 0.787866 | 3.130197 | 1.8556 | 3.167331 | 2.396188 | 1.357165 | 1.505896 | 1.029786 | 0.879436 | 1.200214 |
| **TNFRSF8** | 5.303533 | 3.073631 | 7.039667 | 1.199706 | 3.151155 | 4.451431 | 3.08757 | 2.450765 | 4.868602 | 4.448126 | 3.411684 | 6.511694 | 5.747874 | 5.967261 | 10.24226 | 9.390592 | 11.1336 | 6.862551 | 8.786022 | 9.047766 | 10.0393 | 12.87233 | 7.914922 | 10.40186 |
| **TNFRSF9** | 1.060707 | 4.098175 | 1.759917 | 2.399412 | 4.20154 | 2.670858 | 1.02919 | 1.225383 | 0.695515 | 1.482709 | 2.558763 | 1.627924 | 5.109221 | 2.983631 | 2.363597 | 1.043399 | 3.711201 | 1.055777 | 1.198094 | 3.619107 | 4.517687 | 3.089359 | 1.758872 | 3.200571 |
| **TNFSF10** | 2.121413 | 20.49088 | 5.27975 | 5.998531 | 7.352695 | 9.793148 | 6.175141 | 12.25383 | 6.955146 | 8.896252 | 7.676288 | 18.72112 | 65.78122 | 122.9256 | 55.15061 | 86.60212 | 62.16262 | 118.7749 | 98.64307 | 74.19168 | 106.4166 | 85.98715 | 83.5464 | 98.41757 |
| **TNFSF11** | 11.66777 | 12.29453 | 15.83925 | 4.798825 | 8.403079 | 9.793148 | 5.14595 | 2.450765 | 7.650661 | 8.896252 | 5.970447 | 3.255847 | 4.470568 | 4.773809 | 6.302927 | 11.47739 | 10.2058 | 4.750997 | 3.993646 | 8.14299 | 6.525547 | 5.148931 | 4.397179 | 6.801214 |
| **TNFSF12** | 22.27484 | 12.29453 | 19.35908 | 28.79295 | 21.0077 | 23.14744 | 26.75894 | 17.15536 | 12.51926 | 18.53386 | 22.17594 | 21.16301 | 68.97448 | 47.14136 | 51.21128 | 54.25675 | 39.89541 | 68.09762 | 72.285 | 46.14361 | 52.20438 | 50.45952 | 82.22724 | 70.0125 |
| **TNFSF13B** | 102.8885 | 140.3625 | 96.79542 | 149.9633 | 94.53464 | 135.3235 | 144.0866 | 142.1444 | 122.4106 | 123.8062 | 117.7031 | 150.5829 | 232.4696 | 310.2976 | 151.2702 | 320.3235 | 143.809 | 244.9403 | 239.6188 | 327.9815 | 201.79 | 256.9317 | 303.8451 | 276.8494 |
| **TNFSF15** | 4.242826 | 3.073631 | 5.27975 | 1.199706 | 3.151155 | 0.890286 | 8.233521 | 4.901531 | 4.173088 | 1.482709 | 0.852921 | 2.441885 | 3.831916 | 1.790178 | 1.575732 | 1.043399 | 2.783401 | 4.750997 | 8.386658 | 3.619107 | 5.019652 | 1.029786 | 3.957461 | 5.601 |
| **TNFSF4** | 26.51766 | 21.51542 | 36.95825 | 32.39207 | 17.85654 | 23.14744 | 41.1676 | 22.05689 | 34.77573 | 78.58356 | 21.32302 | 77.32637 | 8.941137 | 13.12798 | 9.45439 | 17.21608 | 24.12281 | 10.55777 | 14.77649 | 13.57165 | 13.55306 | 6.69361 | 11.87238 | 11.202 |
| **TNFSF8** | 13.78918 | 22.53996 | 17.59917 | 17.99559 | 18.90693 | 35.61145 | 42.19679 | 14.70459 | 25.73404 | 23.72334 | 28.14639 | 25.23281 | 32.57128 | 22.07887 | 21.27238 | 25.04158 | 20.41161 | 35.89642 | 33.54663 | 23.52419 | 28.11005 | 38.61698 | 31.65969 | 37.60671 |
| **TOLLIP** | 60.46027 | 47.12901 | 61.59708 | 68.38325 | 47.26732 | 71.22289 | 95.71468 | 50.24069 | 68.16043 | 83.77304 | 71.64536 | 87.90787 | 40.87377 | 31.02976 | 46.48408 | 44.86616 | 27.83401 | 35.89642 | 57.10914 | 39.81017 | 58.22796 | 57.66803 | 64.19881 | 60.01071 |
| **TP53** | 143.1954 | 109.6262 | 91.51567 | 209.9486 | 128.147 | 200.3144 | 207.8964 | 207.0897 | 169.7056 | 195.7175 | 191.0543 | 208.3742 | 191.5958 | 131.8765 | 139.4523 | 185.725 | 122.4696 | 183.1773 | 214.8582 | 192.7174 | 217.3509 | 229.6423 | 184.6815 | 221.6396 |
| **TRAF1** | 6.364239 | 8.19635 | 8.799583 | 8.397943 | 7.352695 | 10.68343 | 23.67137 | 15.92998 | 15.99684 | 17.7925 | 14.49966 | 15.46527 | 3.193263 | 2.983631 | 4.727195 | 1.043399 | 0.9278 | 0.527889 | 4.393011 | 2.261942 | 1.505896 | 1.544679 | 4.836897 | 3.600643 |
| **TRAF2** | 14.84989 | 32.7854 | 24.63883 | 27.59324 | 24.15885 | 22.25715 | 29.84651 | 23.28227 | 31.99367 | 28.17146 | 33.26392 | 30.93055 | 5.747874 | 8.354166 | 6.302927 | 6.260394 | 14.8448 | 7.39044 | 10.78285 | 7.238213 | 9.537338 | 6.178717 | 11.43266 | 8.4015 |
| **TRAF3** | 85.91723 | 94.25803 | 63.357 | 119.9706 | 63.0231 | 84.57719 | 100.8606 | 83.32603 | 79.28866 | 78.58356 | 81.02749 | 91.16372 | 44.70568 | 49.52827 | 34.6661 | 30.25857 | 36.18421 | 35.36853 | 37.93964 | 49.76272 | 35.13756 | 41.70634 | 35.17743 | 38.40686 |
| **TRAF4** | 303.3621 | 550.18 | 288.6263 | 490.6798 | 366.5843 | 314.271 | 470.3399 | 423.9824 | 388.0971 | 553.0503 | 427.3134 | 440.3533 | 22.35284 | 19.69196 | 17.33305 | 20.34628 | 16.7004 | 14.78088 | 16.37395 | 20.80986 | 22.58843 | 17.50637 | 24.18448 | 28.005 |
| **TRAF5** | 6.364239 | 6.147263 | 7.039667 | 8.397943 | 5.251925 | 4.451431 | 10.2919 | 3.676148 | 9.04169 | 10.37896 | 7.676288 | 15.46527 | 12.77305 | 8.354166 | 10.24226 | 9.912291 | 7.422402 | 14.25299 | 11.98094 | 8.14299 | 15.05896 | 16.47658 | 17.58872 | 18.40328 |
| **TRAF6** | 66.82451 | 82.98805 | 36.95825 | 76.78119 | 51.46886 | 53.41717 | 67.92655 | 72.29758 | 63.29183 | 71.91137 | 69.93952 | 75.69844 | 30.01667 | 39.98065 | 27.5753 | 34.43217 | 32.47301 | 26.39443 | 40.73519 | 41.16734 | 29.61595 | 38.61698 | 40.01433 | 43.60778 |
| **TREX1** | 76.37087 | 71.71807 | 65.11692 | 67.18355 | 63.0231 | 69.44232 | 85.42278 | 84.55141 | 67.46492 | 66.72189 | 75.90996 | 77.32637 | 104.1004 | 81.15475 | 77.21085 | 81.90683 | 51.95681 | 111.9124 | 105.4323 | 77.3584 | 77.30264 | 72.08504 | 94.53934 | 116.8209 |
| **TYK2** | 70.00663 | 69.66898 | 63.357 | 65.98384 | 68.27502 | 89.02862 | 95.71468 | 74.74835 | 77.20212 | 97.85877 | 85.29209 | 89.53579 | 127.0919 | 104.4271 | 147.3309 | 142.424 | 99.27463 | 102.9383 | 144.9694 | 101.7874 | 111.4363 | 119.9701 | 156.5396 | 172.0307 |
| **UBE2L3** | 116.6777 | 63.52172 | 73.9165 | 111.5727 | 84.03079 | 77.4549 | 108.065 | 120.0875 | 93.19896 | 108.9791 | 101.4976 | 125.3501 | 88.13406 | 84.13839 | 67.75646 | 68.86434 | 64.94602 | 74.96017 | 89.05832 | 85.95378 | 82.32229 | 79.29354 | 91.0216 | 100.4179 |
| **VCAM1** | 4.242826 | 2.049088 | 5.27975 | 4.798825 | 4.20154 | 4.451431 | 1.02919 | 1.225383 | 2.086544 | 4.448126 | 2.558763 | 5.697732 | 5.109221 | 2.386905 | 3.151463 | 4.173596 | 1.8556 | 3.69522 | 2.396188 | 1.809553 | 4.015721 | 2.059572 | 3.957461 | 1.600286 |
| **VTN** | 3.18212 | 5.122719 | 8.799583 | 2.399412 | 3.151155 | 0.890286 | 1.02919 | 3.676148 | 2.782058 | 2.965417 | 1.705842 | 0.813962 | 3.831916 | 3.580357 | 1.575732 | 3.130197 | 0.9278 | 2.111554 | 2.396188 | 2.261942 | 3.011791 | 2.574466 | 1.758872 | 5.200928 |
| **XBP1** | 292.755 | 247.9396 | 179.5115 | 551.8648 | 199.5731 | 249.2801 | 297.4359 | 273.2603 | 264.2955 | 209.8033 | 250.7588 | 205.9323 | 58.75604 | 74.59077 | 79.57445 | 84.51532 | 76.07962 | 78.12751 | 69.88881 | 96.8111 | 64.25154 | 72.59993 | 72.99317 | 84.81514 |
| **XCL1** | 7.424946 | 3.073631 | 5.27975 | 4.798825 | 7.352695 | 2.670858 | 4.11676 | 4.901531 | 6.259631 | 2.965417 | 4.264605 | 4.069809 | 1.915958 | 7.75744 | 6.302927 | 4.173596 | 8.350202 | 3.167331 | 1.198094 | 0.904777 | 4.015721 | 1.029786 | 2.198589 | 2.8005 |
| **XCR1** | 21.21413 | 22.53996 | 24.63883 | 14.39647 | 23.10847 | 28.48916 | 10.2919 | 6.126914 | 12.51926 | 21.49928 | 13.64674 | 11.39546 | 12.1344 | 12.53125 | 11.03012 | 12.52079 | 12.0614 | 8.446217 | 12.3803 | 11.30971 | 7.529478 | 18.53615 | 14.95041 | 16.00286 |
| **lu** | 6.364239 | 6.147263 | 1.759917 | 2.399412 | 9.453464 | 2.670858 | 4.11676 | 1.225383 | 2.782058 | 5.18948 | 5.117526 | 2.441885 | 1.277305 | 2.983631 | 3.151463 | 4.695296 | 2.783401 | 3.167331 | 5.99047 | 4.523883 | 3.011791 | 2.059572 | 2.198589 | 4.000714 |
| **ZBTB16** | 8.485652 | 4.098175 | 1.759917 | 1.199706 | 3.151155 | 1.780572 | 1.02919 | 8.577679 | 5.564117 | 1.482709 | 0.852921 | 1.627924 | 3.831916 | 4.773809 | 3.151463 | 4.695296 | 18.556 | 4.750997 | 0.399365 | 4.976272 | 4.015721 | 3.604252 | 6.595768 | 0.800143 |
| **ZEB1** | 27.57837 | 20.49088 | 22.87892 | 38.3906 | 23.10847 | 21.36687 | 38.08003 | 23.28227 | 28.5161 | 41.51584 | 26.44055 | 36.62828 | 4.470568 | 6.563988 | 7.878658 | 6.260394 | 6.494602 | 3.167331 | 6.789199 | 9.500155 | 7.027513 | 10.29786 | 9.673793 | 9.201642 |
| **cGAS** | 68.94592 | 118.8471 | 105.595 | 94.77679 | 109.24 | 70.33261 | 107.0358 | 105.3829 | 105.7182 | 96.37606 | 76.76288 | 91.97768 | 22.99149 | 35.80357 | 30.72677 | 41.21426 | 33.40081 | 27.4502 | 39.13774 | 36.19107 | 45.17687 | 26.77444 | 35.17743 | 29.20521 |
| **sCTLA4** | 5.303533 | 5.122719 | 3.519833 | 5.998531 | 7.352695 | 9.793148 | 13.37947 | 8.577679 | 5.564117 | 4.448126 | 5.117526 | 6.511694 | 5.747874 | 2.983631 | 7.090792 | 4.695296 | 4.639001 | 6.334663 | 3.993646 | 5.42866 | 8.533408 | 6.178717 | 4.836897 | 6.001071 |
| **ABCF1** | 43.48897 | 42.0063 | 29.91858 | 57.5859 | 51.46886 | 70.33261 | 80.27683 | 40.43763 | 60.50977 | 71.91137 | 56.29278 | 55.3494 | 44.70568 | 50.72172 | 52.78701 | 63.64734 | 50.10121 | 54.37252 | 59.10597 | 49.31033 | 40.15721 | 58.69781 | 70.35486 | 62.41114 |
| **ALAS1** | 13.78918 | 18.44179 | 15.83925 | 20.395 | 11.55423 | 30.26973 | 26.75894 | 11.02844 | 20.86544 | 22.24063 | 12.79381 | 18.72112 | 21.07554 | 17.90178 | 22.84811 | 15.12929 | 15.7726 | 19.53188 | 23.56251 | 12.66687 | 21.08254 | 32.95316 | 25.50364 | 36.80657 |
| **EEF1G** | 4439.057 | 4392.219 | 3621.909 | 4874.406 | 3017.756 | 4457.663 | 4573.721 | 5421.093 | 3562.426 | 4936.678 | 4994.705 | 5727.035 | 5109.86 | 3691.945 | 3654.122 | 4129.773 | 2365.891 | 4034.652 | 4833.91 | 4694.434 | 4837.438 | 5016.089 | 5284.969 | 5955.463 |
| **G6PD** | 38.18543 | 39.95721 | 43.99792 | 53.98678 | 25.20924 | 36.50173 | 58.66383 | 53.91684 | 43.81742 | 54.11887 | 40.94021 | 52.09355 | 66.41987 | 62.05952 | 57.51421 | 67.82094 | 49.17341 | 69.1534 | 63.09961 | 59.71526 | 48.69062 | 67.451 | 67.71655 | 64.81157 |
| **GAPDH** | 1056.464 | 941.5557 | 862.3592 | 1220.101 | 864.4668 | 1192.983 | 1103.292 | 1491.291 | 864.5246 | 974.1396 | 1072.975 | 1178.617 | 4971.911 | 4613.886 | 4062.236 | 4933.191 | 3845.732 | 5273.607 | 5652.208 | 5908.191 | 5331.372 | 5270.961 | 5671.921 | 5838.642 |
| **GUSB** | 44.54967 | 47.12901 | 31.6785 | 44.38913 | 23.10847 | 38.28231 | 42.19679 | 41.66301 | 49.38154 | 45.96397 | 28.99931 | 30.11659 | 58.11739 | 37.59375 | 40.18116 | 50.08315 | 22.26721 | 44.87053 | 53.51486 | 47.95316 | 55.71813 | 41.19145 | 36.9363 | 56.01 |
| **HPRT1** | 71.06734 | 53.27628 | 59.83717 | 77.9809 | 46.21694 | 99.71205 | 88.51035 | 75.97373 | 73.02903 | 86.73846 | 75.05704 | 91.16372 | 173.7135 | 120.5387 | 120.5435 | 134.0768 | 126.1808 | 130.3885 | 160.944 | 170.098 | 158.119 | 157.0424 | 144.2275 | 153.6274 |
| **OAZ1** | 1530.599 | 1157.734 | 1096.428 | 1536.824 | 1091.35 | 1300.708 | 1566.427 | 1670.197 | 1539.174 | 1503.467 | 1765.546 | 1738.622 | 1805.471 | 1636.82 | 1293.676 | 1790.994 | 1220.057 | 1752.59 | 1918.148 | 1940.746 | 1834.683 | 1768.658 | 1910.574 | 1998.757 |
| **POLR1B** | 21.21413 | 13.31907 | 8.799583 | 9.597649 | 13.655 | 16.02515 | 30.8757 | 12.25383 | 24.34301 | 15.56844 | 23.02887 | 30.93055 | 12.1344 | 10.74107 | 14.96945 | 9.912291 | 7.422402 | 17.42032 | 19.96823 | 10.85732 | 19.07468 | 19.56594 | 28.14194 | 24.40436 |
| **POLR2A** | 119.8598 | 109.6262 | 102.0752 | 179.9559 | 117.6431 | 129.0915 | 159.5245 | 192.3851 | 137.0164 | 137.8919 | 182.5251 | 167.6761 | 102.8231 | 82.94493 | 95.33177 | 114.7739 | 62.16262 | 82.35061 | 104.6335 | 83.23945 | 76.80067 | 100.4042 | 120.4827 | 105.6189 |
| **PPIA** | 77.43158 | 63.52172 | 75.67642 | 134.3671 | 67.22464 | 87.24804 | 121.4444 | 120.0875 | 103.6317 | 111.2031 | 92.96838 | 113.1407 | 79.83158 | 59.07589 | 63.81713 | 87.64552 | 62.16262 | 78.65539 | 99.84116 | 89.1205 | 90.35373 | 111.2169 | 107.2912 | 125.2224 |
| **RPL19** | 4381.779 | 4599.177 | 4213.241 | 5753.791 | 3908.482 | 4267.142 | 5706.859 | 5550.984 | 4308.713 | 5092.363 | 5500.487 | 4944.004 | 5191.607 | 4670.575 | 4577.5 | 5116.307 | 3042.257 | 4558.318 | 6150.615 | 5089.369 | 5535.17 | 5562.905 | 6101.086 | 6035.477 |
| **SDHA** | 40.30685 | 59.42354 | 51.03758 | 53.98678 | 39.91463 | 42.73374 | 41.1676 | 60.04375 | 45.20845 | 47.44668 | 48.61649 | 48.83771 | 44.06703 | 38.19047 | 44.12049 | 39.64916 | 32.47301 | 32.2012 | 37.93964 | 26.69091 | 30.61988 | 43.25102 | 35.61715 | 38.00678 |
| **TBP** | 28.63908 | 31.76086 | 35.19833 | 49.18795 | 22.05808 | 40.06288 | 53.51788 | 26.95842 | 47.29499 | 55.60157 | 40.08728 | 48.02374 | 26.18476 | 17.90178 | 39.39329 | 28.69347 | 19.48381 | 22.17132 | 41.53392 | 28.95285 | 39.15328 | 40.16166 | 39.13489 | 40.00714 |
| **TUBB** | 180.3201 | 95.28257 | 121.4343 | 202.7503 | 119.7439 | 178.9475 | 271.7062 | 196.0612 | 185.7024 | 194.2348 | 214.0832 | 243.3746 | 80.47023 | 84.13839 | 67.75646 | 70.42944 | 52.88461 | 88.15739 | 91.05514 | 94.54916 | 73.78888 | 82.89779 | 78.7095 | 96.41721 |

**Supplementary table S4:** Relative expression of full list of genes detected using NanoString Technologies. Library scaling normalisation was performed with DESeq2 prior to fitting the model
