# Supplementary figures and images for: Phenotypic and Transcriptomic Analysis of Peripheral Blood Plasmacytoid and Conventional Dendritic Cells in Early Drug Naïve Rheumatoid Arthritis
Source: Front Immunol. 2018 May 9;9:755. doi: 10.3389/fimmu.2018.00755 (PMC5968398; doi:10.3389/fimmu.2018.00755)

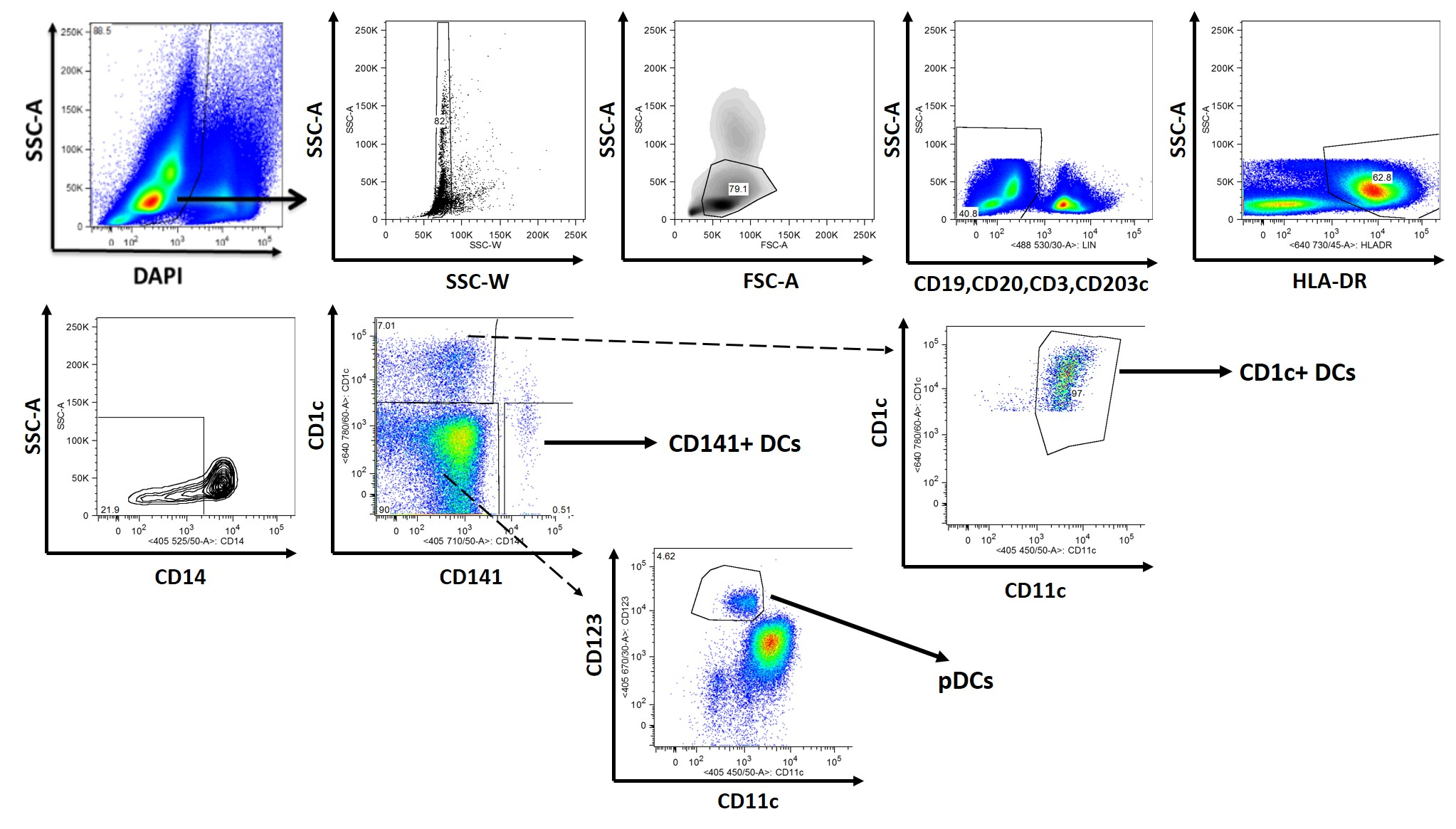

Supplement: Figure S1 — Plasmacytoid DCs (pDCs), CD1c+ dendritic cells (DCs), and CD141+ DCs flow cytometry gating strategy. Gating strategy is shown to identify DCs from isolated PBMCs. pDCs, CD1c+ DCs, and CD141+ DCs were defined as CD19−CD20−CD3−CD203c−HLA-DR+CD14−CD1c−CD141− or dimCD11c− CD123, CD19−CD20−CD3−CD203c−HLA-DR+CD14−CD1c+CD141− or dimCD11c+ and CD19−CD20−CD3−CD203c−HLA-DR+CD14−CD1c+CD141highCD11c−, respectively. [file Image_1.tif]

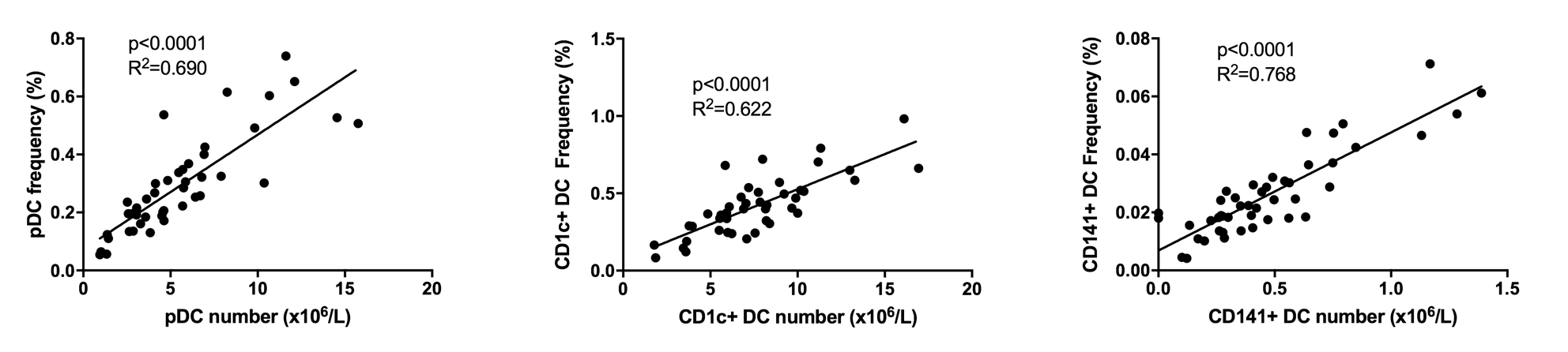

Supplement: Figure S2 — Plasmacytoid DC (pDC), CD1c+ DC, and CD141+ DC number and frequency in early RA whole blood. Plots depict early RA DC number (×106/L) and frequency (%) in the whole blood for (A) pDCs, (B) CD1c+ DCs, and (C) CD141+ DCs. Linear regression. [file Image_2.tiff]
